# Predictive values of trigger tools for identifying adverse events in hospitalized patients using a medical record review: a systematic review

**DOI:** 10.1093/intqhc/mzaf119

**Published:** 2025-11-06

**Authors:** Luis Meave Gutiérrez-Mendoza, Elizabeth Manias, Patricia Nicholson

**Affiliations:** School of Nursing and Midwifery, Centre for Quality and Patient Safety Research, Institute for Health Transformation, Deakin University, Geelong, VIC, Australia; School of Nursing and Midwifery, Faculty of Medicine, Nursing and Health Sciences, Monash University, Melbourne, VIC, Australia; School of Medicine, Faculty of Health, Deakin University, Geelong, VIC, Australia; School of Nursing and Midwifery, Centre for Quality and Patient Safety Research, Institute for Health Transformation, Deakin University, Geelong, VIC, Australia

**Keywords:** Predictive value, Medical records, Prevalence, Adverse event, Hospitals, Patient safety

## Abstract

**Background:**

Efforts to identify the prevalence rate of adverse events have been implemented in hospital settings using different methods. The trigger tool method constitutes one option and involves a retrospective review of paper-based, electronic, or hybrid medical records. The aim of the systematic review was to provide a comprehensive description of the predictive value of trigger tools used to identify adverse events in hospitalized patients.

**Methods:**

A systematic search of MEDLINE, EMBASE, CINAHL, and the Cochrane Library was conducted for studies published between 2000 and October 2024. Eligible studies were peer-reviewed, published in English or Spanish, and reported a trigger tool methodology used to identify the prevalence of adverse events. Two independent reviewers extracted and synthesized the data on study characteristics, methodologies, and outcomes. When reported, tool predictive values were pooled by calculating the arithmetic mean across studies. The risk of bias was assessed using the Joanna Briggs Institute critical appraisal checklist for prevalence studies.

**Results:**

In total, 100 studies from 37 countries were included, 21 high-, 7 upper-middle, 7 lower-middle, and 2 low-income countries. Thirty-four studies reported a predictive value that involved individual triggers (*n* = 20) and the original tool (*n* = 14). The Institute for Healthcare Improvement Global Trigger Tool was the most frequent trigger tool used to identify adverse events in hospitalized patients, with an average positive predictive value of 54.5%, negative predictive value of 80.9%, sensitivity of 86.6%, and specificity of 68.2%. An average positive predictive value of 43.9%, negative predictive value of 37.8%, sensitivity of 84.5%, and specificity of 11.5% was reported for the Harvard Medical Practice Study.

**Conclusion:**

Based on the available evidence, the Institute for Healthcare Improvement Global Trigger Tool demonstrates relatively strong predictive values in identifying adverse events in hospitalized patients, with its flexibility and feasibility further supporting its selection as a suitable tool.

## Introduction

Adverse events in hospitalized patients constitute a significant public health problem in high-, middle-, and low-income countries [1, 2]. These events, which have been identified as one of the leading causes of death by the World Health Organization (WHO), are also the third most common cause of death in the USA [3]. The financial impact is substantial, with an average of 15% of public hospital budgets in high-income countries spent addressing adverse events [4]. The significant financial burden of adverse events in hospitalized patients within the Organisation for Economic Co-operation and Development (OECD) countries, where trillions of dollars are spent on their consequences, highlights the need for a reliable method to identify them [4].

Different methods used to identify adverse events in hospitalized patients include (i) retrospective medical record reviews, (ii) voluntary or mandatory incident reporting systems, (iii) patient safety and health quality indicators, and (iv) patient self-reporting post discharge [5–10]. The prevalence of adverse events can vary depending on which method is implemented [10]. When an adverse event is identified by one method, it may not be identified using a different method [5–10].

The retrospective medical records review method used to identify adverse events has been widely accepted as the ‘gold standard’ [11]. The Harvard Medical Practice Study (HMPS) and the Institute for Healthcare Improvement Global Trigger Tool (IHI-GTT) are two prominent examples that use this approach. The HMPS was published in 1991 by Brennan *et al.* as a feasible option to identify medical errors in hospitalized patients with predefined screening criteria [12]. The time-consuming nature of using the HMPS and the significant staff involvement for medical record reviews necessitated the development of a more efficient method. In 2003, the Institute for Healthcare Improvement developed the Global Trigger Tool as an alternate method, with an updated tool published in 2009 [13]. The IHI-GTT has been officially translated into the local language of several countries [14, 15], while others have adapted, modified, unified triggers according to the local settings, or used explicitly designed triggers for particular areas of interest [16, 17].

Use of a trigger tool involves a two-stage process. In the initial stage, a primary reviewer, typically a nurse, reviews medical records to identify triggers, clues, or predefined screening criteria that suggest the probability that the patient has experienced an adverse event [13]. This process aims to purposefully locate adverse events, followed by an in-depth assessment. Subsequently, in the second stage, a secondary reviewer, usually a physician, validates the presence of an adverse event by reviewing the medical records flagged with positive triggers, clues, or those meeting the screening criteria. This method, adaptable to paper-based, electronic, or hybrid medical records, has been successfully replicated across high-, middle-, and low-income countries and employed to identify the prevalence of adverse events in diverse hospital settings, including public teaching, non-teaching, and private organizations [18–22].

Previous systematic reviews have not addressed the predictive values of trigger tools [10, 11, 23–25]. The positive predictive value (PPV) indicates the probability that a patient has experienced an adverse event during hospitalization if a positive trigger is identified in their medical record. Probabilities are influenced by sensitivity, specificity, and prevalence values. The PPV for the IHI-GTT of 30.4% was reported in one systematic review involving eight studies [26]. The negative predictive value (NPV) of 99.0% was reported in one study [26]. The PPV for the HMPS (33.4%) was reported in a systematic review involving 12 studies [26], while no evidence of the NPV was found. This lack of information about the NPV constitutes an existing gap in the literature to be addressed [11]. Therefore, the aim of our systematic review was to provide a comprehensive description of the predictive value of trigger tools used to identify adverse events in hospitalized patients.

## Methods

### Design, search strategy, and data sources

This systematic review adhered to the Preferred Reporting Items for Systematic Reviews and Meta-Analyses (PRISMA) guideline [27] and was registered in PROSPERO 1 January 2023 (CRD42023384335). Eligible studies were identified by searching CINAHL, Cochrane Library, EMBASE, and MEDLINE, CINAHL. The search was limited to peer-review research articles published in English or Spanish, between 2000 and October 2024, as one author is proficient in both languages. Reference lists of included articles were also screened. The complete search strategy is outlined in Supplementary File S1.

### Eligibility criteria

Only primary research that reported the use of a trigger tool to identify the prevalence of adverse events was included. Research designs including cohort studies, case control studies, cross-sectional studies, and randomized controlled trials conducted in hospital settings in high-, middle-, and low-income countries were included. Articles comprising reviews, secondary research, posters, opinions, published conference abstracts, grey literature, outpatient departments, community settings, and studies conducted in patients younger than 18 were excluded.

### Study selection

**Figure 1:**
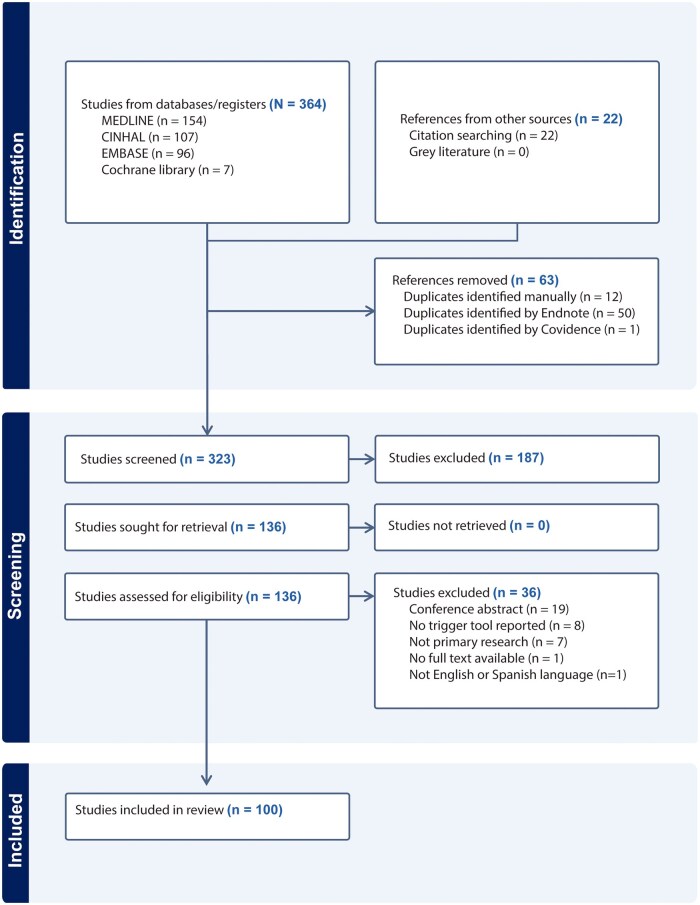
Systematic review flow diagram detailing the search results and the selection process

A search of four databases was conducted by one investigator, with all retrieved articles imported into EndNote^®^ 20. Following removal of duplicates, all references from the library were imported into Covidence^®^. Two independent reviewers screened article titles and abstracts to identify studies that included any information on trigger tools used to identify or measure adverse events in hospitalized patients according to inclusion criteria. The full text of all eligible articles was reviewed by the two independent reviewers. Any disagreement between the two reviewers was resolved by discussion, or by consulting a third reviewer until consensus was reached. The reference lists of included papers were reviewed to include any relevant articles not identified in the original search. One hundred articles were included in the systematic review (Fig. 1).

### Data extraction

A standardized data extraction form was developed using Microsoft Word^®^ and Microsoft Excel^®^ based on information reported in each article (Supplementary File S2, Table 1). The relevant information extracted included: (i) author, year, and country; (ii) aim of the study; (iii) research design; (iv) setting; (v) sample size; (vi) inclusion and exclusion criteria, (vii) type of trigger tool, (viii) stages of the review process and staff involved, (ix) results, and (x) predictive values of the trigger tool.

**Table 1.** List of countries included in the systematic review where a trigger tool method has been used to identify adverse events in hospitalized patients based on their gross national income (GNI) per capita

High-income countries	(*n* = 86)
Australia [30], Austria [31], Belgium [32], Canada [19, 33], Denmark [34], Dutch [35], Finland [36, 37], Germany [38], Ireland [39], Italy [40, 41], Japan [42, 43], Korea [44–46], New Zealand [47], Norway [48–52], Norway & Sweden^a^ [14, 53, 54], Portugal [55, 56, 57], Singapore [58], Spain [59–69], Sweden [15, 70–78], Switzerland [16, 79, 80], UK [17, 81–83], and USA [5, 7, 8, 21, 22, 84–104].	

Upper-middle-income countries	(*n* = 10)
Argentina [105, 106], Brazil [107, 108], China [109, 110], Iran [111], South Africa^b^ [20, 112], Thailand [113], and Turkey [6].	

Lower-middle-income countries	(*n* = 3)
Egypt^b^ [20], Jordan^b^ [20], India [114], Kenia^b^ [20], Morocco^b^ [20], Palestine [116], and Tunisia^b^ [20, 116].	

Low-income countries	(*n* = 1^b^)
Sudan^b^ [20] and Yemen^b^ [20]	

a Three studies were conducted in Norway and Sweden.

b One study was counted for two low-income countries. In addition, this article included four lower-middle and one upper-middle-income countries.

### Data synthesis

A narrative synthesis was undertaken through a structured process that involved organizing and summarizing the relevant studies characteristics [28]. The data were described based on relevant characteristics, such as differences in hospital setting, type of medical records, trigger tool used, number, background, and years of experience of the reviewers and predictive values reported. A meta-analysis of the predictive values across studies was not undertaken due to methodological heterogeneity. Findings were thoroughly examined to draw conclusions addressing the aim of the systematic review. The percentage reported in the results sections was calculated using Microsoft Office Excel 365.

### Quality assessment

The risk of bias was assessed using the Joanna Briggs Institute (JBI) critical appraisal checklist for studies reporting prevalence data [29], which evaluates nine domains such as sample frame, sampling method, sample size, data collection, and statistical analysis. No modifications were made for this review. Two independent reviewers conducted the appraisal, resolving disagreements by consensus or consultation with a third reviewer; 11.0% (*n* = 11) of articles were double-checked, with 100% agreement (Supplementary File S3, Table 2).

**Table 2.** Predictive values of the Harvard Medical Practice Study and the Institute for Healthcare Improvement Global Trigger Tool extracted from the retrieved studies

Harvard Medical Practice Study					
	Sensitivity	Specificity	PPV	NPV	Prevalence of AEs
Aibar (2015) [61]			53.6		3.6%
Brennan (2004) [86]	89.0				4.7%
Kobayashi (2008) [42]	89.3				28.0%
	85.7				25.0%
Valencia Martín (2022) [62]	73.9	11.5	37.9	37.8	11.9%
Unbeck (2013) [70]			40.3		30.0%
Total	**84.4**	**11.5**	**43.9**	**37.8**	**17.2%**

Institute for Healthcare Improvement—Global Trigger Tool					
	Sensitivity	Specificity	PPV	NPV	Prevalence of AEs
Perez-Zapata (2017) [68]	86.0	93.6	89.0	92.0	31.7%
Classen (2011)[7]	94.9	100.0			33.2%
Pierdevara (2016) [57]	97.8	74.8	69.8	98.0	36.0%
Hu (2019) [109]			38.7		68.5%
Mevik (2019) [48]	59.0	92.0	58.0		15.1%
Val (2020) [64]	91.2	4.8	49.1	35.2	50.2%
Unbeck (2013) [70]			30.4		30.0%
Guzman Ruíz (2015) [65]	91.3	32.5	42.5	87.1	35.4%
Müller (2016) [112]			46.8		24.4%
Pérez-Zapata (2022) [66]	86.2	79.5	66.5	92.4	31.5%
Total	**86.6**	**68.2**	**54.5**	**80.9**	**35.6%**

AEs: adverse events; PPV: positive predictive value; NPV: negative predictive value.

Values in bold represent the average sensitivity, specificity, PPV, NPV, and prevalence of AEs.

## Results

The systematic review included 100 articles, including 37 countries (Table 1); 86% of the studies were conducted in 21 high-income countries, 10.0% in seven upper-middle-income countries, 3.0% in three lower-middle-income countries, and 1.0% included eight countries: one upper-middle income, five lower-middle-income, and two low-income countries.

Of the 100 studies included in this systematic review, 66.0% did not report a predictive value, highlighting a significant gap in the literature; 16% of studies reported a predictive value for the trigger tool used in the study, 12.0% reported predictive values for individual triggers, and 6.0% reported predictive values for both the trigger tool and individual triggers used in the studies. Across these 34 studies reviewed, predictive values for individual triggers varied widely, ranging from 0 to 100%. Of these, only 23.5% reported a threshold of >20% for individual triggers, which was interpreted as indicators of medium to good trigger [16, 32, 58, 59, 84, 85, 114, 117]. Notably, only one study explicitly defined a PPV above >20% as an acceptable threshold for assessing the tool’s overall quality [60].

Among the 15 studies that reported the implementation of the HMPS, only 33.3% reported predictive values [42, 61, 62, 70, 86]; two studies reported a PPV of 53.6% [61] and 40.3%, respectively [70], while another reported a PPV of 37.9%, a NPV of 37.8%, a sensitivity of 73.9%, and specificity of 11.5% [62]. Additionally, one study reported a sensitivity of 89.0% for the tool [86], and another study involving two independent reviewers reported a sensitivity of 89.3% and 85.7%, respectively [42].

Of the 75 studies that reported the implementation of the IHI-GTT, 13.3% reported an average PPV of 54.5%, an average NPV of 80.9%, sensitivity of 86.6%, and specificity of 68.2%. The average tool predictive values were calculated by computing the arithmetic mean of values reported across studies that provided predictive values. The predictive values of the HMPS and IHI-GTT are presented in Table 2.

The hospital settings included academic (51.0%), non-academic (8.0%), private (1.0%), or a combination that was described as mixed hospitals (30.0%). In 10.0% of the studies, the type of hospital was not reported (Table 3).

**Table 3.** Characteristics of the 100 included studies

Study	Country	Type of hospital	Number of hospitals	Sample size number of records	Study period number of months	Type of Medical Records	Type of Trigger Tool	Original and Validated Trigger Tool	Modified, Adapted, or Translated	Developed New Triggers
Aibar *et al.* (2015) [61]	Spain	Mixed	41	816	1 week	NR	HMPS	No	Yes	No
Aikawa *et al.* (2021) [43]	Japan	NR	1	50	3	EMR	IHI-GTT	No	Yes	No
Ali *et al.* (2024) [30]	Australia	NR	4	500	36	EMR	Medication-related triggers	No	No	Yes
Asavaroengchai *et al.* (2009) [113]	Thailand	Academic	1	576	1	NR	IHI-GTT	Yes	No	No
Baker *et al.* (2004) [19]	Canada	Mixed	20	3745	12	NR	HMPS	Yes	No	No
Bates *et al.* (2023) [101]	USA	Mixed	11	2809	12	EMR	IHI-GTT	No	Yes	Yes
Bjertnaes *et al.* (2015) [9]	Norway	Mixed	23	400	3	NR	IHI-GTT	No	Yes	No
Brennan *et al.* 1991 (2004) [86]	USA	Mixed	51	30121	12	NR	HMPS	Yes	No	No
Brösterhaus *et al.* (2020) [38]	Germany	Academic	3	120	2	EMR & PBMR	IHI-GTT	No	Yes	No
Brown *et al.* (2019) [103]	USA	NR	1	426	7	EMR	IHI-GTT	No	Yes	No
Carnevali *et al.* (2013) [32]	Belgium	Academic	1	240	12	EMR	IHI-GTT	No	Yes	No
Classen *et al.* (2008) [88]	USA	NR	2	65	NR	PBMR	IHI-GTT	Yes	No	No
Classen *et al.*, (2011) [7]	USA	Academic	3	795	1	EMR	IHI-GTT	Yes	No	No
Cohen *et al.* (2005) [104]	USA	Non-academic	1	580	24	NR	IHI-GTT	No	Yes	No
Connolly *et al.* (2021) [39]	Ireland	Mixed	8	3177	24	PBMR	HMPS	Yes	No	No
Croft *et al.* (2016) [98]	USA	Academic	1	296	11	NR	IHI-GTT	Yes	No	No
Davis *et al.* (2002) [47]	New Zealand	Mixed	13	6579	12	NR	HMPS	Yes	No	No
Deilkas *et al.* (2015) [51]	Norway	Mixed	24	40851	46	NR	IHI-GTT	No	Yes	No
Deilkas *et al.* (2017) [14]	Norway & Sweden	Mixed	86	30127	12	NR	IHI-GTT	No	Yes	No
Deilkas *et al.* (2021) [53]	Norway & Sweden	Mixed	77 to 84	142004	72	EMR	IHI-GTT	No	Yes	No
Dolci *et al.* (2020) [79]	Switzerland	Mixed	2	538	NR	EMR	IHI-GTT	No	Yes	No
Dotta *et al.* (2024) [106]	Argentina	Academic	1	320	6	EMR	IHI-GTT	No	Yes	No
El Saghir *et al.* (2021) [80]	Switzerland	Academic	1	1008	12	EMR	IHI-GTT	No	Yes	Yes
Fajreldines *et al.* (2022) [105]	Argentina	NR	2	830	2	EMR	IHI-GTT	Yes	No	No
Franklin *et al.* (2009) [82]	UK	Academic	1	207	2	EMR	IHI-GTT	No	Yes	No
Franklin *et al.* (2010) [83]	UK	Academic	1	207	2	EMR & PBMR	IHI-GTT	No	Yes	No
Garrrett Jr *et al.* (2013) [91]	USA	Mixed	25	17295	36	EMR	IHI-GTT	Yes	No	No
Gómez-López *et al.*, (2019) [69]	Spain	Academic	1	464	6	NR	IHI-GTT	No	Yes	Yes
Good *et al.* (2011) [96]	USA	Mixed	12	2369	12	NR	IHI-GTT	Yes	No	No
Griffey *et al.* (2020) [89]	USA	Academic	1	1726	13	EMR	Emergency care triggers	No	Yes	Yes
Griffin and Classen, (2008) [93]	USA	NR	11	854	12	NR	IHI-GTT	No	Yes	Yes
Grossmann *et al.* (2019) [16]	Switzerland	Non-academic	1	240	12	EMR	IHI-GTT	No	Yes	Yes
Gunningberg *et al.* (2019), Sweden [74]	Sweden	Private	63	64917	48	EMR	IHI-GTT	No	Yes	No
Guzmán-Ruíz *et al.* (2015) [6^5^]	Spain	Non-academic	1	291	12	NR	IHI-GTT	No	Yes	Yes
Härkänen *et al.* (2015) [36]	Finland	Academic	1	463	12	EMR	IHI-GTT	No	Yes	No
Haukland *et al.* (2017) [52]	Norway	Non-academic	1	6720	48	NR	IHI-GTT	No	Yes	No
Hommel *et al.* (2020) [76]	Sweden	Mixed	24	1998	36	EMR & PBMR	IHI-GTT	No	Yes	No
Hu *et al.* (2019) [109]	China	Academic	1	480	12	EMR	IHI-GTT	No	Yes	No
Hug *et al.*, (2010) [102]	USA	Non-academic	6	1200	18	NR	IHI-GTT	No	Yes	No
Hwang *et al.* (2014) [45]	Korea	Academic	1	629	6	EMR	IHI-GTT	Yes	No	No
Hwang *et al.* (2018) [46]	Korea	Academic	2	1152	7	EMR	IHI-GTT	No	Yes	No
Kennerly *et al.* (2013) [94]	USA	Academic	8	16172	48	EMR	IHI-GTT	No	Yes	No
Kennerly *et al.* (2014) [95]	USA	Non-academic	8	9017	60	EMR	IHI-GTT	No	Yes	No
Kobayashi *et al.* (2008) [42]	Japan	Academic	1	200	12	NR	HMPS	Yes	No	No
Kurutkan *et al.* (2015) [6]	Turkey	Academic	1	229	12	NR	IHI-GTT	Yes	No	No
Letaief *et al.* (2010) [116]	Tunisia	Academic	1	620	12	NR	HMPS	Yes	No	No
Lima-Junior *et al.* (2023) [108]	Brazil	Mixed	2	370	12	NR	HMPS	Yes	No	No
Lipitz-Snyderman *et al.* (2017) [84]	USA	Academic	1	400	12	NR	Oncology triggers	No	Yes	Yes
Magnéli *et al.* (2019) [75]	Sweden	Mixed	24	1998	36	EMR	IHI-GTT	No	Yes	No
Mattsson *et al.* (2014) [34]	Denmark	Academic	1	240	12	NR	IHI-GTT	No	Yes	Yes
Mayor *et al.* (2017) [81]	UK (Welsh)	Mixed	11	4833	36	EMR & PBMR	IHI-GTT & HMPS	Yes	No	No
Menéndez-Fraga *et al.* (2021) [67]	Spain	Academic	1	240	12	EMR	IHI-GTT	No	Yes	No
Merten *et al.* (2013) [35]	Dutch	Mixed	21	7917	14	NR	HMPS	Yes	No	No
Mevik *et al.* (2016) [50]	Norway	Academic	1	1920	12	NR	IHI-GTT	No	Yes	No
Mevik *et al.* (2019) [48]	Norway	Academic	3	1233	10	EMR & PBMR	IHI-GTT	No	Yes	No
Moraes *et al.* (2021) [107]	Brazil	Academic	1	220	12	EMR & PBMR	IHI-GTT	No	Yes	No
Mortaro *et al.*, (2021) [40]	Italy	Non-academic	1	1320	66	EMR	IHI-GTT	No	Yes	No
Mull *et al.* (2015) [5]	USA	Non-academic	1	273	1 to 3	EMR	IHI-GTT	No	Yes	No
Müller *et al.* (2016) [112]	South Africa	Academic	1	160	8	PBMR	IHI-GTT	No	Yes	No
Naessens *et al.* (2009) [99]	USA	Academic	1	235	12	NR	IHI-GTT	Yes	No	No
Naessens *et al.* (2010) [100]	USA	Academic	3	1138	61	NR	IHI-GTT	Yes	No	No
Najjar *et al.* (2013) [115]	Palestine	Mixed	2	640	4	NR	IHI-GTT	No	Yes	Yes
Nilsson *et al.* (2012) [73]	Sweden	Academic	1	128	24	PBMR	IHI-GTT	No	Yes	No
Nilsson *et al.* (2018) [15]	Sweden	Mixed	63	64917	48	NR	IHI-GTT	No	Yes	No
Nilsson *et al.* (2020) [71]	Sweden	NR	NR	2552	6	NR	Mental health triggers	No	Yes	No
Nwulu *et al.* (2013) [17]	United Kingdom	Academic	1	467	12	EMR	IHI-GTT	No	Yes	No
O’Leary *et al.* (2013) [90]	USA	Academic	1	250	12	EMR	Mix from IHI-GTT & HMPS	No	Yes	No
Ock *et al.* (2015) [44]	Korea	NR	1	96	NR	EMR	IHI-GTT & HMPS	No	Yes	No
Otero *et al.* (2021) [59]	Spain	Mixed	12	720	3	NR	Medication-related triggers	No	Yes	Yes
Pandya *et al.* (2020) [114]	India	Academic	1	463	10	NR	IHI-GTT	Yes	No	No
Paulander *et al.* (2024) [54]	Sweden	Academic	5	60	3	NR	IHI-GTT	No	Yes	No
Pérez Zapata *et al.* (2017) [68]	Spain	Academic	1	350	12	EMR	IHI-GTT	No	Yes	No
Pérez Zapata *et al.* (2022) [66]	Spain	Mixed	31	1132	9	NR	IHI-GTT	No	Yes	No
Pettersson *et al.* (2020) [72]	Sweden	Academic	1	163	24	EMR	IHI-GTT	No	Yes	No
Pierdevara *et al.* (2016) [57]	Portugal	Academic	1	90	9	EMR	IHI-GTT	No	Yes	No
Pierdevara *et al.*, (2020) [55]	Portugal	Academic	1	90	9	NR	IHI-GTT	No	Yes	Yes
Resar *et al.* (2006) [92]	USA	Mixed	54	12074	46	NR	IHI-GTT	No	Yes	No
Rutberg *et al.* (2014) [77]	Sweden	Academic	1	960	48	EMR	IHI-GTT	No	Yes	No
Sajith *et al.* (2021) [58]	Singapore	Academic	1	515	6	EMR & PBMR	Mental health triggers	No	Yes	Yes
Samal *et al.* (2022) [8]	USA	Academic	1	88	22	EMR	IHI-GTT	No	Yes	No
Sari et al (2015) [111]	Iran	NR	4	1162	6	NR	HMPS	Yes	No	No
Scarpis *et al.* (2023) [41]	Italy	Academic	1	291	3	PBMR	IHI-GTT	No	Yes	No
Schildmeijer *et al.* (2012) [78]	Sweden	NR	5	50	8	NR	IHI-GTT	No	Yes	No
Schmied *et al.* (2024) [31]	Austria	Academic	1	421	12	NR	IHI-GTT	No	Yes	No
Sekijima *et al.* (2020) [97]	USA	Academic	1	300	4	NR	IHI-GTT	No	Yes	No
Sharek *et al.* (2011) [87]	USA	Mixed	10	2400	72	EMR & PBMR	IHI-GTT	Yes	No	No
Sousa *et al.* (2014) [56]	Portugal	Mixed	3	1669	12	NR	HMPS	Yes	No	No
Storesund *et al.* (2019) [49]	Norway	Mixed	2	700	29	EMR	IHI-GTT	No	Yes	No
Suarez *et al.* (2014) [63]	Spain	Academic	1	1440	72	EMR	IHI-GTT	No	Yes	No
Thomas *et al.* (2000) [21]	USA	Mixed	28	14700	12	NR	HMPS	Yes	No	No
Thomas *et al.* (2002) [22]	USA	NR	NR	500	12	PBMR	HMPS	Yes	No	No
Toribio-Vicente *et al.* (2018) [60]	Spain	Academic	1	233	12	EMR & PBMR	IHI-GTT	No	Yes	No
Unbeck *et al.* (2013) [70]	Sweden	Academic	1	350	12	EMR	IHI-GTT & HMPS	Yes	No	No
Val *et al.* (2020) [64]	Spain	Academic	1	251	12	EMR	IHI-GTT	No	Yes	Yes
Valencia-Martín *et al.* (2022) [62]	Spain	Mixed	34	9975	1	EMR	HMPS	Yes	No	No
Valkonen *et al.* (2023) [37]	Finland	Academic	1	834	60	EMR	IHI-GTT	No	Yes	No
Wilson et al (2012) [20]	Egypt, Jordan, Kenya, Morocco, Tunisia, Sudan, South Africa, and Yemen	Mixed	26	15548	12	NR	HMPS	Yes	No	No
Wong *et al.* (2015) [33]	Canada	Academic	1	141	4	EMR & PBMR	IHI-GTT	No	Yes	Yes
Xu *et al.* (2020) [110]	China	Academic	1	240	12	EMR	IHI-GTT	No	Yes	Yes
Zadvinskis *et al.* (2018) [85]	USA	Academic	1	317	1	EMR	IHI-GTT	No	Yes	No

EMR: Electronic medical records; PBMR: Paper based medical records; IHI-GTT: Institute for Healthcare Improvement—Global Trigger Tools; NR: Not recorded.

Nine hundred and thirty-two hospitals were involved with 552 623 medical records retrospectively reviewed using a trigger tool in the studies retrieved. Although two studies did not mention the number of hospitals involved, the total number of medical records reviewed was reported [22, 71]. The medical records reviewed involved either electronic (42.0%), paper-based (6.0%), or a combination (hybrid) (10.0%). However, in 42.0% of the studies, the type of medical record review was not explicitly reported (Table 3). The study period for the medical record review varied widely between studies, ranging from one month [7, 85, 62, 113] to 72 months [53, 63, 87]. One study was conducted over a 1-week period [61], and in three studies [44, 79, 88], the period was not reported.

Of the included studies, 75.0% reported using the IHI-GTT, 15.0% used the HMPS, 3.0% used the IHI-GTT and HMPS [70, 44, 81], and 6.0% did not use either of the trigger tools mentioned [30, 58, 59, 71, 84, 89], and a subset of triggers from both the IHI-GTT and the HMPS [90] was used in 1.0%. Of the six studies that did not use either of the previously mentioned trigger tools, all involved the development of modifications or alternatives based on the IHI-GTT, comprising mental health triggers (*n* = 2) [58, 71], medication-related event triggers (*n* = 2) [30, 59], emergency department triggers (*n* = 1) [89], and oncology triggers (*n* = 1) [84].

All studies reported a collaborative two-stage review process. One study [70] reported an additional third stage for consensus in case of disagreement to validate an adverse event by secondary reviewers. A comprehensive review process was followed, involving two or more reviewers in 86.0% of the studies, with 8.0% not reporting the number of reviewers. Six studies only involved a single reviewer due to limited resources [22, 64, 65, 72, 82, 83]. In 60.0% of the studies, the review process involved at least one nurse and one physician, fostering a sense of inclusivity. The first stage was conducted with nurses as primary reviewers in 50.0% and physicians in the second stage as secondary reviewers in 40.0% of the studies. Other healthcare staff also participated in the first stage, including pharmacists, medical students, and residents, and in 7.0% of studies, the background of the members involved in the review team were not explicitly reported [7, 17, 64, 85, 91, 92, 66]. In most studies (72.0%), a physician validated the adverse events independently of the first reviewer. Seventy-eight per cent of the reviewers included internal staff where the study was conducted. Both internal and external reviewers were involved in 12.0% of the studies [20, 39, 61, 62, 73, 87, 113, 93–97], 4.0% involved external reviewers [21, 22, 88, 91], and 6.0% did not report this information [7, 19, 86, 30, 64, 47].

The time limit for reviewing medical records was reported in 46.0% of the studies with 80.4% reporting a 20-min time limit as supported by the IHI-GTT. However, a wide range was reported—from 15 min [80] when only the medication module of the IHI-GTT was implemented to 45 min when the full tool was used [73]. It is important to note that one study reported a time limit of 20 min for the first stage with the second stage requiring an additional 6 min using the IHI-GTT method [48]. One study reported the first stage of the HMPS required 30 min with the second stage completed in 32 min [19].

The prevalence of adverse events using the trigger tools varied widely from 1.1% when only one trigger was used (pressure ulcers) [74] to 83.0% when the original medication module trigger tool was used [82]. The prevalence of adverse events ranged from 7.2% [45] to 35.1% [98] when the original IHI-GTT tool was used, which was higher than the prevalence of adverse events reported when the original HMPS tool was used (range: 2.9% [21] to 28.0% [42]).

Quality assessment showed high methodological quality among the included studies, with domain-specific compliance rates as follows: appropriate sample frame (99%), appropriate sampling method (99%), adequate sample size (98%), detailed description of subjects and setting (97%), adequate coverage in data analysis (100%), valid identification methods (100%), standardized and reliable measurement (100%), and appropriate statistical analysis (99%). The response rate was not applicable in this study. Overall, the risk of bias was low.

## Discussion

### Statement of principal findings

This systematic review has highlighted key findings. The IHI-GTT and the HMPS are the most frequently cited trigger tools in the literature for retrospective medical record reviews. Despite the widespread use of trigger tools to identify the prevalence of adverse events in hospitalized patients in high-, middle-, and low-income countries, the predictive values of the tool are only reported in about one in seven studies.

Significant differences in the predictive values and prevalence of adverse events between the IHI-GTT and the HMPS have been identified in the systematic review. The prevalence of adverse events identified with the trigger tools was higher when the IHI-GTT was implemented compared with the HMPS. This can be explained as the IHI-GTT includes three times the number of triggers (53 vs 18 triggers) and is divided into six modules. On the one hand, the modular integration of the IHI-GTT allows the users to use the entire tool, selected modules, or selected triggers. On the other hand, limited studies reported using the full IHI-GTT [6, 7, 34, 45, 87, 91, 96, 98–100, 105, 113, 114]. This explains why the HMPS, which has fewer triggers, had better predictive values reported in a previous systematic review [26] but a lower prevalence of adverse events than the IHI-GTT. In addition, it is evident from this systematic review that the IHI-GTT has been translated into languages other than English [53, 55], constituting an asset of the method that can be easily implemented in different countries and local hospital settings.

In recent years, modifications or alternatives to the original IHI-GTT have been developed. These include automated detection systems using electronic health records, natural language processing, and machine learning algorithms, which aim to identify adverse events with greater efficiency and accuracy while reducing the time required from clinical staff to review medical records [17, 48, 79, 90]. Moreover, adapted versions of the IHI-GTT have been tailored to specific contexts, including mental health [58, 71], medication-related events [30, 59], emergency care [89], and oncology [84]. Recognizing these innovations highlights both the ongoing relevance and the limitations of the original IHI-GTT. However, despite the proliferation of these approaches, evidence regarding their validity and predictive performance, particularly their predictive values remains limited.

The predominance of the IHI-GTT and HMPS in the literature likely reflects the established validation, broad applicability, and the availability of training resources [13], which facilitate the use of these tools to identify adverse events with retrospective medical records review to improve patient safety. In contrast, newer or modified trigger tools may have limited uptake due to a narrower clinical scope, the need for local adaptation, and limited published evidence on the validity and reliability of these tools [30, 84, 58, 59, 81, 89]. Further research should examine the comparative performance of these emerging tools and the potential to enhance adverse event detection in specialized settings.

The retrospective medical records review has been widely standardized as a two-stage process for both the IHI-GTT and the HMPS. This approach is generally standardized to reduce physicians’ time, as only one study reported a single physician conducting both stages [65]. The first stage was not always conducted by two experienced nurses, and the validation in the second stage did not involve a physician, as recommended by the IHI-GTT instructions and methods section [13]. However, other staff such as pharmacists and respiratory therapists also participated in the first stage. Although the IHI-GTT studies did not adhere to the planned methods or staff involvement, reliable results were obtained. This is important because this systematic review provides strong evidence that the IHI-GTT is a feasible and user-friendly tool, producing reliable outcomes when used by various healthcare professionals.

The total number of reviewers and teams involved in the medical records review could be a significant factor that influences the medical record review process. The minimum of three participants recommended by the IHI-GTT was not adhered to in all of the studies reviewed. When only one pharmacist was involved in the review of the medication trigger tool, a prevalence of 83% for adverse drugs events was reported [82]. In Spain, when only one physician [65] conducted both stages, an adverse events prevalence of 35.4% was reported, highlighting that the full review process can be conducted involving one physician with reliable outcomes. The two studies where one physician conducted both stages reported an average adverse event of 16.3% (range 12.8% to 19.0%) with the HMPS [22] and 35.4% with the IHI-GTT [65]. This could be explained as more consistency or less heterogeneity criteria exist when the review team is limited. However, the time involved could be time-consuming when only one reviewer is involved.

The experience of the reviewers in both stages were not reported in 64.0% (*n* = 64) of the studies. In 36% of the studies (*n* = 36), the experienced of the reviewers involved in the process ranged from no experience to more than 30 years of experience [5, 58, 49, 75]. The most frequent reported included nurses with more than 5 years of experiences and physicians with more than 10 years of experience [16, 19, 34, 44, 56, 58, 67, 79, 96, 107]. This is important because the evidence suggests that an experienced reviewer needs to be involved in the process.

### Strengths and limitations

This systematic review had several strengths. First, this review adhered to the PRISMA statement and followed the registered study protocol in PROSPERO. Second, the search strategy included studies from 37 countries. Third, this is the first systematic review to include predictive values of the IHI-GTT and HMPS trigger tools used to identify the prevalence of adverse events in hospitalized patients, offering a fresh perspective on patient safety. Hence, the outcomes presented are generalizable.

We identified the following limitations. First, only articles published in English and Spanish were included; articles published in another language may have been excluded. Second, despite the strong search strategy and manual review of citations, some articles may not have been included. Third, a meta-analysis was not conducted because despite the prevalence of adverse events reported in almost all the studies, no reported data or inconsistent reporting formats were identified, therefore a narrative synthesis was conducted. Fourth, heterogeneity across studies was identified, with some studies conducting a retrospective record review using specific triggers or modules, thereby limiting the ability to synthesize findings meaningfully.

### Interpretation within the context of the wider literature

The two trigger tools reported in the literature to measure the prevalence of adverse events in hospital settings included the HMPS and the IHI-GTT methods. However, the IHI-GTT has several advantages over the HMPS, as presented in this systematic review. First, the original IHI-GTT can be used (including full number of triggers and modules) or only specific triggers or modules [13]. The tool can be translated into official languages or used in its original language [53, 55]. The two-stage retrospective medical review can be conducted by a single experienced and trained healthcare professional, such as a nurse, physician, pharmacist, or other healthcare providers, which is an asset of the IHI-GTT, as it enhances both the reliability and feasibility of the tool [83, 65, 72]. Both methods have been reproduced and implemented in different countries, with varying outcomes regarding the prevalence of adverse events. However, a higher prevalence of adverse events was identified when using the IHI-GTT, which included electronic, paper-based, or hybrid medical records [16, 75, 76, 107, 109].

### Implications for policy, practice, and research

The findings in this systematic review are of significant value for future policy and practice in identifying the prevalence of adverse events across different hospital settings worldwide. The use of a trigger tool to determine the prevalence of adverse events offers a structured approach to improving healthcare outcomes. Currently, the IHI-GTT presents an accessible and feasible option for implementation in hospital settings without requiring substantial economic investment, making it particularly advantageous in resource-limited environments [13]. In future practice and research, the development of digital trigger tools has the potential to reduce the time spent identifying triggers in hospitals equipped with electronic medical records [17, 48, 79]. However, this approach necessitates the supervision of healthcare professionals to validate the findings effectively [85, 87]. Despite advances in digital tools, retrospective medical records reviews will continue to be critical. Moreover, the validation of adverse events will remain dependent on the expertise of physicians or other experienced healthcare professionals [50, 87, 90].

To enhance healthcare process and hospital outcomes globally, recommendations include the implementation of the IHI-GTT in projects aiming to identify the prevalence of adverse events. This approach is recommended across all countries, regardless of their income levels or type of medical records utilized, ensuring a standardized and effective methodology for improving patient safety and healthcare quality.

## Conclusions

The predictive values of trigger tools described in this systematic review provide evidence supporting the IHI-GTT as an appropriate tool for identifying the prevalence of adverse events in hospitalized patients. The IHI-GTT can be used in different countries and hospital settings, and its feasibility and reliability ensure high reliability of the outcomes.

## Supplementary Material

mzaf119_Supplementary_Data

## Data Availability

Data extracted from the systematic review is available as a supplementary file.
