# Supplementary material for: Predictive values of trigger tools for identifying adverse events in hospitalized patients using a medical record review: a systematic review
Source: Int J Qual Health Care. 2025 Nov 6;37(4):mzaf119. doi: 10.1093/intqhc/mzaf119 (PMC12622303; doi:10.1093/intqhc/mzaf119)
Supplement: mzaf119_Supplementary_Data [file mzaf119_supplementary_data.zip › Supplement_Data (10)/6.docx]

**Supplementary File 2.** Data extraction table

**Table 1.** Summary of included studies

| **Author, year and country** | **Aim of the study** | **Research design** | **Setting** | **Sample size (number of records)** | **Patient characteristics and inclusion criteria (age, sex, etc.)** | **Type of Trigger Tool** | **Reviewers: training and experience** | **Results** | **Predictive values of Trigger Tool to identify a true adverse event** | **Predictive values threshold** |
| --- | --- | --- | --- | --- | --- | --- | --- | --- | --- | --- |
| Aibar et al. (2015), Spain (1) | To measure the prevalence of AEs in obstetric patients | Retrospective medical record review | 41 hospitals in Spain (Mixed hospitals) | N=816; Random sample of women from 24 hospitals over 1 week period (June 4 to June 10, 2005) | Women with a hospital stay of at least 24 hours in the obstetric department. | The National Adverse Effects Study in Spanish hospitals (ENEAS). There are 19 triggers based on the HMPS | Two-stage review. First stage: nurses from each participating hospital independently reviewed medical records. Second stage: a team of external trained reviewers (clinicians or surgeons) validated the AEs | % of patients with ≥1 AE was 3.6% (n=29) | PPV = 53.6% (95% CI 45.7-61.5; 82/153) | Not reported |
| Aikawa et al. (2021), Japan (2) | To assess the intensive care trigger tool in a Japanese setting | Retrospective medical record review (electronic medical records) | One intensive care unit (Academic hospital) | N=50; Random sample of medical records over a 3-month period (April to June 2018) | Patients aged 18 or older, with a hospital stay of at least 48 hours.  Males n=32 and females n=18 | The IHI Intensive Care Unit Trigger Tool (Japanese version). The tool includes 22 triggers in two modules (18 for care and four for procedure) | Two-stage review. Two teams independently reviewed medical records. First stage: two intensive care nurses independently reviewed medical records (20-minute time limit). Second stage: one intensivist validated the AEs | % of patients with ≥1 AE not reported  Team I 63 vs Team II 51 AEs per 1000 patient days  Team I 42 vs Team II 34 AEs per 100 admissions | Not reported | Not reported |
| Ali et al. (2024), Australia (3) | To identify the factors related to ADEs in hospitalised patients | Retrospective medical record review (Electronic medical records) | Four public hospitals | N=500; Random sample medical records over 36 months (July 2018 to June 2021) | Patients aged 65 or older, with an unplanned admission. Planned admission and incomplete medical records excluded. Males n=198 and females n=302 | Medication trigger tool based on previously published studies and own developed trigger tool | Two-stage review. First stage: One pharmacist reviewed medical records. Second stage: Three pharmacist and a geriatrician validated the ADEs | % of patients with ≥1 AE was  18.2% (n=91) | Not reported | Not reported |
| Asavaroengchai et al. (2009), Thailand (4) | To measure the incidence of AEs | Retrospective medical record review | One academic hospital | N=576; Random sample of medical records over one month period (January 2008) | Hospital stay of at least 24 hours, completed, closed medical records, patients discharged > 30 days from five departments (obstetrics, gynecology, surgery, internal medicine, paediatrics, and orthopedics),  Males n=221 and females n=355. (<1 year n=108, 1-14 years n=46, 15-29 years n=89, 30-44 years n=107, 45-59 years n=89, and >60 n=137) | IHI-GTT (53 triggers) | Two-stage review. First stage: two registered nurses reviewed medical records. Second stage: a physician consultant validated the AEs | % of patients with ≥1 AE = 24.0%  50.4 AEs per 1000 patient days  41 AEs per 100 patients | Not reported | Not reported |
| Baker et al. (2004), Canada (5) | To measure the incidence of AEs in hospitalised patients | Retrospective medical record review | 20 hospitals including 5 teaching hospitals, 5 large and 10 small community hospitals (Mixed hospitals) | N=3,745; Random sample of medical records over 12 months (January to December 2000) | Patients aged 18 or older, with a hospital stay of at least 24 hours; Obstetrics, rehabilitation, and psychiatry patients excluded. Males and females | HMPS-18 triggers | Two-stage review. First stage: nurses or health record professionals review medical records. Second stage: Physicians validated the AEs | % of patients with ≥1 AE was  6.8% (n=255) | Not reported | Not reported |
| Bates et al. (2023), USA (6) | To measure the prevalence of AEs in 11 hospitals | Retrospective medical record review. (electronic medical records) | 11 hospitals (Mixed hospitals) | N=2,809; Random sample of medical records over 12 months (January to December 2018) | Patients 18 years and older; hospice admission, rehabilitation psychiatric care, and hospital stay that did not include two midnights excluded.  Males n=1,225, females n=1,561, sex not recorded n=22 | 66 triggers (51 from the original IHI-GTT).  Four and three new triggers were created and disaggregated in the care module respectively, two new triggers in the medication module, and six new triggers in the perinatal module | Two-stage review. First stage: nine nurses independently reviewed medical records. Second stage: one of eight physicians validated the AEs | % of patients with ≥1 AE was 23.6%  34.8 AEs per 100 admissions (95% CI 29.2-40.5) | Not reported | Not reported |
| Bjertnaes et al. (2015), Norway (7) | To compare the prevalence of AEs reported by patients and detected during a medical record review | A cross-sectional survey and retrospective medical record review and cross-sectional survey | Mixed hospitals. All Norwegian hospitals (19 hospital trusts and four private hospitals) | N=400; Random inpatients discharged over three months (March to May 2022) | Paediatric, rehabilitation and psychiatric excluded. Sex not recorded | The Patient-Reported Incident in Hospital Instrument is a validated national survey comprised of 73 closed-ended items. Most experience items had a five-point response format ranging from 1 (not at all) to 5 (to a considerable extent\) | Two-stage review, according to the GTT manual (Norwegian version). Forty-seven GTT teams participated in the medical record review. Specific information about the participants in the first and second stages not recorded | The survey response rate was 46.4%  % of patients reporting ≥1 AE was 4.9% range (1.3% to 24.5%).  15.96 AEs per 100 admissions with the GTT (4.35 to 29.17). | Not reported | Not reported |
| Brennan et al. 1991 (2004), USA (8) | To measure AEs in hospitalised patients using the Harvard Medical Practice Study | Retrospective medical record review | 51 acute care hospitals in New York | N=30,121; Random sample of medical records from 2,671,863 discharges (January to December 1984) | Psychiatric patients excluded. Sex not recorded | The Harvard Medical Practice Study (18 screening criteria) | Two-stage review. First stage: trained nurses and medical records analysts reviewed medical records. Second stage: Two physicians (internists or surgeons) validated the AEs | % of patients with ≥1 AE was 4.2% (n=1,278) | Sensitivity = 89% | Not reported |
| Brösterhaus et al. (2020), Germany (9) | To measure the incidence of AEs using the IHI-GTT | Retrospective medical record review (both electronic and paper based medical records) | Three academic hospitals | N=120; Random sample of medical records (n=40) per hospital over two months (January to July 2017) | Patients aged 18 or older, with a hospital stay of at least 24 hours, discharged 30 days or more before review commenced. Sex not recorded | IHI-GTT (German version: 48 triggers) was used in two surgical departments and (59 triggers [eleven are specific for neurosurgery) in one neurosurgery department. | Two-stage review. First stage: two primary reviewers (two medical doctoral students, two surgeons, one anaesthetist, and one nurse) independently reviewed medical records in their respective hospitals (20-minute time limit). Second stage: a physician (one surgeon, one surgeon and one neurosurgeon) with primary reviewers validated the AEs | % of patients with ≥1 AE was 32.5% (n=39); 18.9% (surgery), 35.9% (surgery), and 45.3% (neurosurgery)  25.5 (surgery), 36.3 (surgery), and 72.1 (neurosurgery) AEs per 1000 patient days  25 (surgery), 47.5 (surgery) and 60 (neurosurgery) AEs per 100 admissions | Not reported | Not reported |
| Brown et al. (2019), USA (10) | To measure the prevalence of ADEs using three IHI-GTT triggers | Retrospective medical record review (electronic medical records) | One hospital. Details not reported | N=426 over 7 months period (January to August 2017)  INR >6 trigger (n=77), plasma glucose ≤ 50 mg/dL trigger (n=148), and naloxone trigger (n=201). | Patients aged 18 or older, INR > 6 receiving warfarin anticoagulation therapy, plasma glucose ≤ 50 mg/dl, patient receiving opioid medications, with administration of naloxone; pregnant women, and prisoners, excluded. Sex not recorded | IHI-GTT, three triggers of the medication module selected: international normalised ratio (INR) >6, plasma blood glucose ≤50 mg/dL, and naloxone administration | Three 4^th^-year student pharmacists reviewed medical records. Data were collected until 100 adverse events were identified or until August 1, 2017. More information about the review process not recorded | % of patients with ≥1 ADEs related to INR >6 was 16.9% (n=13)  % of patients with ≥1 ADEs related to plasma glucose ≤ 50 mg/dL was 67.6% (n=100)  % of patients with ≥1 ADEs related to naloxone was 49.8% (n=100) | PPV for INR > 6 trigger = 35%, plasma glucose ≤50 mg/dL trigger = 70.4%, and naloxone = 53% | Not reported |
| Carnevali et al. (2013), Belgium (11) | To measure the ADEs using the adverse drug event (IHI-ADE) trigger tool and IHI-GTT medication module | Retrospective medical records review (electronic medical records) | One academic hospital | N=240; Random sample of medical records (n=20) over 12 months (February 2010 to January 2011) | Patients aged 18 or older, with a hospital stay of at least 24 hours; Incomplete medical records (no discharge letter, no nursing chart, or no treatment documented) excluded. Sex not recorded | Two trigger tools:. The IHI-ADE trigger tool (n=19 triggers) was adapted to the Belgian hospital (‘‘partial thromboplastin time (PTT) >100 seconds” was removed’; ‘oversedation, lethargy, and falls’ was divided into two triggers (n=20). Second, the IHI-GTT medication module (n=11) because M2 (PTT > 100 seconds) excluded | Two-stage review. First stage: two primary record reviewers (one clinical pharmacist and one nurse) independently reviewed medical records (20-minute time limit). Second stage: three physicians validated the ADEs. | % of patients with ≥1 ADE was 25.8%. (Only 77.0% of ADEs were identified with the IHI-GTT)  23 ADEs per 1000 patient days  26 ADEs per 100 admissions | The PPV of the 20 triggers varied from 0 to 67%, with half of them having less than 20% (falls, vitamin, serum glucose >50 mg/dL, oversedation-lethargy, emergency of confused state, antiemetic, antidiarrheals, and transfer to a higher level of care. Three triggers were not identified (flumazenil, digoxin level >2.6 nmol/L, and naloxone) | >20% (acceptable trigger threshold) |
| Classen et al. (2008), USA (12) | To measure the agreement of reviewers identifying AEs using the IHI-GTT | Retrospective medical record review | Two hospital s | N=65; Sample of medical records selected (n=15) for training and (n=50) for testing | Two skilled physician reviewers, selected according to the IHI-GTT methodology, examined the medical records for training and testing purposes. Sex not recorded | IHI-GTT (53 triggers) | Two-stage review. First stage: four clinicians (2 pharmacists, a respiratory therapist, and a nurse) independently reviewed medical records (20-minute time limit). Second stage: 2 physicians (a pulmonologist and a family physician) validated the AEs | Inter-rater in the training session ranged from 38.5% to 76.9% (22 AEs in 15 medical records)  In the testing session inter-rater ranged from 66.7% to 93.9% (49 AEs in 50 medical records) | Not reported | Not reported |
| Classen et al. (2011), USA (13) | To compare the rate of AEs detected using the IHI-GTT, voluntary reporting system, and patient safety indicators | Retrospective medical record review (electronic medical records) | Three tertiary care hospitals (Academic hospitals) | N=795; Random sample of medical records over 1 month period (October 1 to October 31, 2004) | Patients aged 18 or older, with a hospital stay of at least 24 hours, and complete discharge summary. Males n=296 and females n= 499 | IHI-GTT (53 triggers) | Two-stage review. First stage: four non-physicians (reviewers' background not recorded). Second stage: two physicians validated the AEs | % of patients with ≥1 AE was 33.2% (n=264)  91 AEs per 1000 patient days  49 AEs per 100 admissions | IHI-GTT had a sensitivity = 94.9%, specificity = 100%  Voluntary reporting system sensitivity = 0% and specificity = 100%, and patient safety indicators sensitivity=8.5% and specificity=98.5% | Not reported |
| Cohen et al. (2005), USA (14) | To measure the incidence of ADEs before and after a medication safety program intervention | Retrospective medical record review | One non-academic hospital | N=580; Random sample of medical records (n=10 to n=20 every month) over 24 months (January 2001 to December 2003) | Patients discharged from the hospital; Adverse drug reactions excluded. Sex not recorded | The IHI-Adverse Drugs Events trigger tool (24 triggers) | Two-stage review. First stage: two reviewers (a clinical pharmacist and a nurse manager) reviewed medical records. Second stage: the same reviewers validated the AEs. In case of disagreement, a physician validated the AEs | % of patients with ≥1 ADE was 31.0%  2.04 to 0.65 ADEs per 1000 doses of medication before and after the medication safety program  5.07 to 1.30 ADEs per 100 patients before and after the medication safety program | Not reported | Not reported |
| Connolly et at. (2021), Ireland (15) | To measure the prevalence of AEs in Irish hospitals | Retrospective medical record review (Paper based medical records) | Eight public hospitals (Mixed hospitals) | N=1,603; Random sample of medical records over 12 months period (January to December 2015) | Patients aged 18 or older, with a hospital stay of at least 24 hours. Psychiatric, obstetrics and patients transferred from an external hospital excluded. Males n=770 and females n=835 | HMPS 18 triggers | Two-stage review. First stage: Ten nurses independently reviewed medical records. Second stage: Three physicians validated the AEs | % of patients with ≥1 AE was  14.8% (n=238) | Not reported | Not reported |
| Croft et al. (2016), USA (16) | To identify the prevalence of AEs in patients at hospital arrival, during hospitalisation, and at discharge | Retrospective medical record review | One academic hospital | N=296; Random sample of medical records over 11 months (January to November 2010) | Patients 18 or older, with hospital stay of 3 days or more, admitted and hospitalised at the medical and surgical departments. Males n=161 and females n=135 | IHI-GTT | Two-stage review. First stage: one reviewer and two physicians review medical records (20-minute time limit). Second stage: a physician validated the AEs | % of patients with ≥1 AE was  35.1% (n=104) during hospitalisation, 39.9% (n=118) before admission, and 8.1% (n=24) after discharge | Not reported | Not reported |
| Davis et al. (2002), New Zealand (17) | To measure the prevalence of AEs in hospitalised patients | Retrospective medical record review | 13 general hospitals (Mixed hospitals) | N=6,579; Systematic sample of medical records over 12 months (January to December 1998) | Patients admitted to any of 20 general hospitals; Psychiatric, rehabilitation and day patients excluded. Males n= 2,967 and females n=3,612 | HMPS-18 triggers | Two-stage review. First stage: nurses review medical records. Second stage: Experienced physicians validated the AEs | % of patients with ≥1 AE was  12.9% (n=62)  80.4% AEs occurred in the hospital and 19.6 outside the hospital | Not reported | Not reported |
| Deilkas et al. (2015), Norway (18) | To measure AEs in Norway | Retrospective medical record review | Mixed hospitals. 2011 and 2012: 19, public hospitals; 2010 and 2013: 18 public hospitals 2010, 2012, and 2013: five private hospitals: 2011: four hospitals included | N=40,851; Random sample of medical records (n=240 per year per hospital) over 46 months (March 2010 to December 2013) | Patients aged 18 or older, with a hospital stay of at least 24 hours; Paediatric, psychiatric and rehabilitation excluded. Sex not recorded according to ethics regulations for personal protection | IHI-GTT (44 of the 53 original triggers) (Norwegian version). Three triggers were modified (a drug to treat allergic reactions, denomination for blood glucose updated, and specific monitoring for anticoagulation therapy) | Two-stage review. At least one GTT team in each hospital. First stage: two nurses reviewed medical records. Second stage: one physician validated the AEs | % of patients with ≥1AE was 15.9% in 2010, 16.1% in 2011, 13.7% in 2012, and 13.0% in 2013 | Not reported | Not reported |
| Deilkas et al. (2017), Norway and Sweden (19) | To compare the prevalence of AEs in hospitalised patients in Norway and Sweden | Retrospective medical record review | Mixed hospitals. Acute care hospitals (n=21) and small non-acute care hospitals (n=2) in Norway and acute care hospitals in Sweden (n=63) | N=30,127 (n=10,986 in Norway and n= 19,141 in Sweden); Random sample of medical records (minimum bimonthly n=10) in Norway and (minimum monthly n=90) in Sweden over 12 months (January -December 2013) | Patients aged 18 or older, with a hospital stay of at least 24 hours; Psychiatric and rehabilitation patients excluded. Sex - not recorded | IHI - GTT, Swedish and Norwegian version (44 of the 53 original triggers) | 45 GTT teams participated in Norway.  In Sweden, each hospital had one or more than one review team. Two-stage review. First stage: one nurse reviewed medical records. Second stage: one or more nurses and one or more physicians validated the AEs | % of patients with ≥1 AE was 13.0% (95% CI 11.7% to 14.3%) in Norway and 14.4% (95% CI 12.6% to 16.3%) in Sweden. | Not reported | Not reported |
| Deilkas et al. (2021), Norway and Sweden (20) | To measure the prevalence of AEs that contributed to death using the GTT in two countries | Retrospective medical record review (electronic medical records) | Mixed hospitals. (55-60) acute care hospitals in Sweden (n=55-60) and acute care hospitals in Norway (n=21-24) | N= 142,004 (n=88,637 in Sweden and n=53,367 in Norway); Random sample of medical records at least (n=10) bimonthly over 72 months (From 2013 to 2018) | Patients aged 18 or older, with a hospital stay of at least 24 hours, and all coding complete; Psychiatric or rehabilitation patients excluded. Sex not recorded | The IHI-GTT Norwegian (n=23 triggers) and Swedish (n=27 triggers) versions with 19 identical triggers | Two-stage review. First stage: two trained registered nurses independently reviewed medical records. Second stage: a physician validated the AEs | % of patients with ≥1 AE was 13.2% in Norway and 13.1% in Sweden  0.23% of hospital admissions in Norway and 0.26% in Sweden AEs contributed to deaths | Not reported | Not reported |
| Dolci et al. (2020), Switzerland (21) | To measure the prevalence of patient falls by implementing an algorithm, the traditional GTT medical record review, and voluntary reporting | Retrospective medical record review (electronic medical records) | Two mixed hospitals (one academic hospital and one rural hospital) | N=538; Random sample of medical records in three phases. First phase, development data set (n=120). The second phase testing the data set (n=120). Third phase, a validation study (n=298) | Patients aged 18 or older, with a hospital stay of at least 24 hours, closed and complete medical records. All types of accidents leading to falls as the cause for hospitalisation excluded. Sex not recorded | The IHI-GTT fall trigger | Two-stage review. First stage: two nurses (with five years of clinical experience) reviewed medical records (20-minute time limit). Second stage: a physician validated the AEs | First phase: % of patients with ≥1 fall = 8.3% (n=20). The algorithm detected 95.0% (n=19), the GTT 90.0% (n=18), and the voluntary report 67.0% (n=14)  Third phase: % of patients with ≥1 fall was 8.3% (n=20). The algorithm detected 100% (n=15), the GTT 93.0% (n=14), and the voluntary report 33.0% (n=5)  The algorithm required seconds to identify the trigger, the medical record review took 11 minutes | First phase: (algorithm, the traditional GTT medical record review, and voluntary report) sensitivity 100%, 82%, and 64%, specificity 91%, 100%, and 100%, PPV 52%, 100%, and 100%, and NPV 100%, 98%, and 96%).  Second phase: sensibility 80%, 100%, and 70% specificity 92%, 100%, and 100%, PPV 47%, 100%, and 100%, and NPV 98%, 100%, and 97%.  Third phase: sensitivity 100%, 93%, and 33%, specificity, 94%, 100%, and 100%, PPV 47%, 100%, 100%, and NPP 100%, 99%, and 97% | Not reported |
| Dotta et al. (2024), Argentina (22) | To measure the prevalence of AEs in an internal medicine department | Retrospective medical record review (Electronic medical records) | One academic hospital | N=320; Random sample medical records over 6 months. (January to July 2022) | Patients aged 18 or older, with a hospital stay of at least 48 hours. Psychiatric excluded. Males n=182 and females n=138 | IHI-GTT (28 triggers from the cares and medication module) | Two-stage review. First stage: Four residents reviewed medical records. Second stage: three physicians validated the AEs | % of patients with ≥1 AE was  29.7% (n=95)  65.1 AEs per 1000 admission days  50.3 AEs per 100 admissions | Not reported | Not reported |
| El Saghir et al. (2021), Switzerland (23) | To measure ADEs using an electronic trigger tool and compared with traditional medical records review | Retrospective medical record review (electronic medical records) | One academic hospital | N=1008; Random sample of medical records (n=84) over 12 months (January to December 2018) | Patients aged 18 or older, with a hospital stay of at least 24 hours, written informed consent, and complete medical records; Patients in the intensive care unit and emergency department excluded. Males n=545 and females n=463 | Three trigger tools. The IHI-GTT (13 triggers) medication module adapted to specific hospital conditions. Tool A was modified for digitally extractable data only (n=10 triggers), and Tool B (n=11 triggers: same ten electronic triggers plus one manual trigger (M1 – *C. difficile*-positive stool) | Two-stage review. First stage: a pharmacy graduate and a medical doctor in training reviewed medical records (15-minute average time limit). Second stage: a clinical pharmacologist and toxicologist validated the ADEs | % of patients with ≥1 AE was 19.0% (n=189) | IHI-GTT: Sensitivity = 100% Specificity=100%  Tool A: Sensitivity=41%, specificity=86%.  Tool B:  Sensitivity=43%, specificity=86% | Not reported |
| Fajreldines et al. (2022), Argentina (24) | To measure the prevalence of AEs in two hospitals in Argentina | Retrospective medical record review (electronic medical records) | Two high-complex hospitals (Mixed hospitals) | N=830; Random sample of medical records over two months period (August to September 2017) | Patients aged 18 or older, with a hospital stay of at least 48 hours. Males n=372 and females n=458 | IHI-GTT | Two-stage review. First stage: one physician from each hospital independently reviewed medical records. Second stage: the same physicians validated the AEs | % of patients with ≥1 AE was 32.0% (n=266)  64.3 AEs per 1000 admission days  32 AEs per 100 admissions | Not reported | Not reported |
| Franklin et al. (2009), UK (25) | To measure medication errors using four methods | Prospective study with retrospective medical record review (electronic medical records) | One academic hospital (surgical ward) | N=207; All patients on the medical ward were included over two 4-week periods. (April 2003 and November/December 2003) n=93 medical records pre-intervention and n=114 post-intervention. The intervention was the implementation of a computerised physician order entry | Inpatients who were on the medical ward during the data collection period. Sex not recorded | IHI-GTT for identification of adverse drugs events (23 triggers) | Two-stage review. First stage: a pharmacist reviewed medical records. Second stage: the research pharmacist validated the prescription errors | 1) The prospective detection of medication errors was 36.0% for the pre-and 24.0% for the post-intervention group.2) A retrospective medical record review identified 69.0% for the pre-intervention group and 83.0% for the post-intervention group. 3) The trigger tool identified 0.0% for the pre-and 2.0% for the post-intervention group, and 4) voluntary reporting identified 1.0% for the pre-and 1.0% for the post-intervention group | Not reported | Not reported |
| Franklin et al. (2010), UK (26) | To implement, an adapted version of the IHI-GTT ADE trigger tool and compare the prevalence of detecting ADEs with retrospective medical record review | Retrospective medical record review (electronic prescribing system) | One surgical ward (Academic hospital) | N=207; All patients on the medical ward were included over two 4-week periods. (April 2003 and November/December 2003) n=93 medical records pre-intervention and n=114 post-intervention. The intervention was the implementation of a computerised physician order entry | Inpatients admitted to the medical ward during the data collection period. Sex not recorded | IHI-GTT for identification of adverse drugs events (23 triggers) | Two-stage review. First stage: a pharmacist reviewed medical records. Second stage: the research pharmacist validated the prescription errors | % of patients with ≥1 ADE was 3.4% (n=7) with the trigger tool and 2.4% with medical record review  0.7 ADEs per 100 patient days with the trigger tool and 0.5 per 100 patient days with medical record review  The trigger tool took 4 minutes vs 44-minute for the medical record review | Sensitivity = 40% compared to medical record review.  Overall PPV = 4% for all ADEs. PPV for individual triggers ranged from 0 to 100%. Five triggers PPV = 100%, one trigger PPV = 67%, one PPV = 22%, one PPV = 1%, six triggers PPV = 0%, and nine triggers not identified. | Not reported |
| Garrrett Jr et al. (2013), USA (27) | To measure the prevalence of AEs using the IHI-GTT | Retrospective medical record review (electronic medical records) | 25 hospitals of the Adventist Health System (Mixed hospitals) | N= 17,295; Random sample of medical records (n=24) over 36 months (January 2009 to December 2011) | Patients aged 18 years or older, with a hospital stay of at least 24 hours; Psychiatric and rehabilitation patients excluded. Sex not recorded | IHI-GTT | Two stage review. First stage: four analysts review medical records. Second stage: A senior physician validated the AEs | % of patients with ≥1 AE was 26.0%  85 AEs per 1000 patient days  38 AEs per 100 admissions | Not reported | Not reported |
| Gómez-López et al. (2019), España (28) | To measure the prevalence of AEs before and after extracorporeal anticoagulation, vascular lines, and hypotension treatment improvement actions in a dialysis unit | Retrospective medical record review | One haemodialysis unit (Academic hospital) | N=464; All haemodialysis sessions over six months were reviewed. Three months (March, May, and July) were reviewed in 2016 (n=208) and 2017 (n=258) | All session conducted in the haemodialysis hospital unit. Sex not recorded | A haemodialysis trigger tool was developed with 14 triggers using the hospital methodology (MIDEA) based on the IHI-GTT. | Two-stage review of nurse notes. First stage: all haemodialysis’s notes were reviewed by two trained nurses. Second stage: two nurses validated the AEs | % of patients with ≥1 AE was 38.9% (n=81) in 2016 before the intervention and 35.3% (n=91) in 2017 after the intervention | Not reported | Not reported |
| Good et al. (2011), USA (29) | To measure the prevalence of AEs | Retrospective medical record review | 12 mixed hospitals | N=2,369 random sample of medical records (n=10-35) over 12 months (July 2006 to June 2007) | Patient aged 18 or older, with a  hospital stay ≥ three days, medical record closed and completed; Psychiatric or addictive diseases patients excluded | IHI-GTT trigger tool (53 triggers) | Two-stages review. First stage: two external nurse auditors reviewed medical records. (20-minute time limit) Second stage: the Office of Patient Safety team validated the AEs | % of patients with ≥1 AE was 39.8% for all admissions and 25.0% for current hospitalisation  68.1 AEs per 1000 patient days for all admissions and 41.6 AEs for current hospitalisation  50.8 AEs per 100 admissions for all admissions and 31.1 AEs for current hospitalisation | Not reported | Not reported |
| Griffey et al. (2020), USA (30) | To measure the incidence of AEs in the emergency department using electronic triggers | Retrospective medical record review (electronic medical records) | One academic hospital | N=1,726; Records selected for review over 13 months (October 2014 to October 2015 | Patients aged 18 or older; patients who left without being seen excluded.  Males n=918 and females n=808 | A new emergency trigger tool was initially developed with 97 triggers (the IHI-GTT only has two specific triggers for the emergency module) | Two-stage review. First stage: three trained nurses independently reviewed medical records (20-minute time limit). Second stage: a physician validated the AEs | % of patients with ≥1 AE was 20.0% (n=346)  21.7 AEs per 100 admissions | Sensitivity >70% for 80 triggers;  Specificity >92% for all triggers.  NPV = 99.4% | Not reported |
| Griffin and Classen. (2008), USA (31) | To identify AEs using a trigger tool in different hospital surgical settings | Retrospective medical record review | 11 hospitals. Hospital types not recorded | N=854; Random sample of medical records (n=20) per hospital with a mean of 4 months per hospital (from 1 to 8 months) over 12 months (October 2003 to October 2004 | Patients aged 18 or older, with a hospital stay of at least 24 hours in orthopaedics or surgical units. Sex not recorded. | A collaborative of the IHI developed and implemented 24 surgical trigger tool triggers | Two-stage review. First stage: Each hospital had its review team. Teams consisted of 3-5 people (surgical nurses, surgeons, anaesthetists, quality improvement staff) with a 20-minute time limit. Second stage: The IHI- project director and a physician validated the AEs | % of patients with ≥1 AE was 14.6% (n=125)  16 AEs per 100 admissions | Not reported | Not reported |
| Grossmann et.al. (2019), Switzerland (32) | To describe the prevalence of adverse events (AEs) in hospitalised patients | Retrospective medical record review (electronic medical records) | One academic hospital | N=240; Random sample of medical records (n=20) over 12 months (September 2016 to August 2017) | Patients aged 18 or older, with a hospital stay of at least 24 hours, discharge summary, and all medical information complete within 30 days of hospital discharge.  Males n=126 and females n=114 | Institute for Healthcare Improvement, Global Trigger Tool (IHI-GTT). Using care module (15 triggers), medication module (12 triggers), surgical module (2 triggers), and three self-developed triggers, in total, 32 triggers (29 of the original IHI-GTT 53 triggers). | Two-stage review. First stage: two trained nurses independently reviewed medical records (20-minute time limit). Second stage: one senior physician validated the AEs | % of patients with ≥1 AE was 60.0% (n=144)  95.7 AEs per 1000 patient days | Positive predictive values (PPV) ranged from 0% (acute dialysis, naloxone or flumazenil) to 100% (readmission within 30 days, stroke, and Clostridium difficile-positive stool) with a median of 41.3 (IQR 23.9-65.5). | 6.3% to 50% (Low to medium predictive values for the triggers) |
| Gunningberg et al. (2019), Sweden (33) | To measure the prevalence of pressure wounds in acute care hospitals | Retrospective medical record review (electronic medical records) | 63 mixed acute care hospitals | N=64,917; Random sample of medical records  Over 48 months (From January 2013 to December 2016) | Patients aged 18 or older, with a hospital stay of at least 24 hours. Males n=30,418 and females n=34,499 | The IHI-GTT (Swedish version) | Two-stage review. First stage: registered nurses reviewed medical records. Second stage: a team including registered nurses and a physician validated the AEs | % of patients with ≥1 pressure injuries (categories 2-4) = 1.0% | Not reported | Not reported |
| Guzmán-Ruíz et al. (2015), Spain (34) | To measure the prevalence of AEs with the IHI-GTT in an internal medicine department | Retrospective medical record review | One non-academic hospital | N=291; Random sample of medical records over 12 months (January to December 2013) | Patients aged 18 or older, with a hospital stay of at least 24 hours; Psychiatric excluded. Males n=153 and females n=138 | IHI-GTT (29 of the 53 original triggers and developed 12 new triggers) | Two-stage review. First stage: one physician reviewed medical records. Second stage: The same physician validated the AEs | % of patients with ≥1 AE was  35.4% (n=103)  63 AEs per 1000 admission days  52 AEs per 100 admissions | Tool Sensitivity=91.3%, Specificity=32.5%, PPV=42.5%, and NPV=87.1% | Not reported |
| Härkänen et al. (2015), Finland (35) | To measure the prevalence of ADEs integrating both the IHI-GTT and IHI-ADEs triggers | Retrospective medical record review (electronic medical records) | One academic hospital | N= 463; Random sample of medical records (n=40) over 12 months (January to December 2011) | Patients aged 18 or older, with a hospital stay of at least 24 hours; Psychiatric and rehabilitation patients excluded. Males n=238 and females n=225 | Multi-professional experts modified the IHI-GTT medication module and IHI-ADEs triggers (22 triggers) | Two-stage review. First stage: two nurses independently reviewed medical records. Second stage: a team (a nurse, a clinician physician, and a clinician pharmacist) validated the AEs | % of patients with ≥1 ADEs was 27.0% (n=125)  61.3 ADEs per 1000 patient days | Not reported | Not reported |
| Haukland et al. (2017), Norway (36) | To measure the rate of AEs in cancer and non-cancer patients during hospitalisation | Retrospective medical record review | One non-academic (public health trust hospital) | N=6,720; Random sample of medical records (n=140) over 48 months (January 2010 to December 2013). Twelve per cent (n=812) of patients had cancer as a primary or secondary diagnosis | Patients aged 18 or older, with a hospital stay of at least 24 hours; Psychiatric or rehabilitation patients excluded. Males n=448 (cancer patients) and n=2,266 (non-cancer patients). Females n=364 (cancer patients), and n=3,642 (non-cancer patients) | IHI-GTT (Norwegian version; 58 triggers). Modification included five triggers (not in English version): three triggers in the surgical module and two triggers in the perinatal module | Two-stage review. Seven different teams in the hospital trust. First stage: two nurses independently review medical records. Second stage: a physician validated the AEs | % of cancer patients with ≥1 AE was 24.2%, and 17.0% for non-cancer patients  37.1 AEs per 1000 patient days (cancer patients) and 36 AEs per 1000 patient days (non-cancer patients) | Not reported | Not reported |
| Hommel et al. (2020), Sweden (37) | To measure the incidence of nursing AEs in patients following a hip arthroplasty | Multicentre cohort study with retrospective medical record review (electronic medical records) | 24 mixed hospitals (six academic hospitals, five county council hospitals, seven local hospitals and six private hospitals) | N=1,998; Random sample of medical records over 36 months (January 2009 to December 2011). Patients were followed for up to 90 days postoperatively | Patients aged 18 or older who had an elective (degenerative joint disease) or acute (fractures) hemi or total hip arthroplasty surgery. Males n=748 and females n=1,250 | The IHI-GTT (38 triggers: Swedish version) with five modules. The perinatal module excluded | Two-stage review. Ten reviewers were involved (registered nurses, medical students and physicians). First stage: reviewers independently reviewed medical records (no time limit). Second stage: the same reviewer validated the AEs. Medical specialists were available for consultation | % of patients with ≥1 AE was 36.4% (n=728) | Not reported | Not reported |
| Hu et al. (2019), China (38) | To measure the AEs in geriatric patients using the IHI-GTT | Retrospective medical record review (electronic medical records) | One academic hospital | N=480; Random sample of medical records (n=20) fortnightly over 12 months (January 2015 to December 2015) | Patients aged 60 or older, with a hospital stay of at least 24 hours; Psychiatry, rehabilitation, and ambulatory patients excluded. Males n=292 and females n=188 | IHI-GTT version. Four modules (43 triggers) of the IHI-GTT were used, including care, medication, surgery, and intensive care unit. perinatal and emergency modules excluded | Two-stage review. First stage: two trained clinical pharmacists reviewed medical records. Second stage: one physician validated the AEs (30-minute time limit) | % of patients with ≥1 AE was 68.5% (n=329)  22.43 AEs per 1000 patient days  127 AEs per 100 admissions | Tool had a PPV = 38.78%. Twenty two triggers had a PPV> 50% and six (C9, C13, S3, S5, S7, and S8) < 10%. Three triggers (M1, M8, and S10) did not identify AEs. Eight AEs were identified without triggers | <10% (Low predictive value for the triggers) |
| Hug et al. (2010), USA (39) | To measure the adverse drugs events (ADEs) in hospitals using a trigger tool | Retrospective medical record review cohort study. | Multicentre: six, non-academic, community hospitals | N=1,200; Random sample medical records (n=200) from each hospital over 18 month period (January 2005 to August 2006) | Patients aged 18 or older, with hospitalisation in one of the six hospitals. Psychiatric and neonatal services excluded. Males n=481 and females n=719 | Rozich et al. 2003 IHI-medication trigger tool (18 of the 24 triggers), Six triggers related to drugs levels toxicity (T14-T19) and one trigger (T24) to be customized by the institution excluded. The trigger “Platelet count <50,000x10^6^/µl” included | Two-stage review. First stage: trained nurses reviewed medical records and extracted data. Second stage: each ADE was independently reviewed by two physicians | % of patients with ≥1 ADEs was 11.7% (n=141)  15 ADEs per 100 admissions | Not reported | Not reported |
| Hwang et al. (2014), Korea (40) | To measure the prevalence of AEs using the GTT | Retrospective medical record review (electronic medical records) | One academic hospital | N=629; Random sample of medical records (n=30 per week) over six months (January to June 2011) | Patients aged 18 or older, with a hospital stay of at least 24 hours; Psychiatric patients excluded. Males n=298 and females n=331 | The IHI-GTT (53 triggers) | Two-stage review. First stage: two quality improvement specialists independently reviewed medical records (20-minute time limit). Second stage: two physicians validated the AEs | % of patients with ≥1 AE was 7.2% (n=45)  12.4 AEs per 1000 patient days  7.79 AEs per 100 admissions | Eight triggers had PPV ≥ 50% (healthcare-associated infection, any procedure complication, medication: other, return to surgery, the occurrence of any operative complication, and intubation/reintubation). 15 triggers were not identified | Not reported |
| Hwang et al. (2018), Korea (41) | To measure the incidence of AEs in two traditional medicine hospitals | Retrospective medical record review (electronic medical records) | Two traditional medicine academic hospitals | N=1,152; Random sample of medical records (n=784) in hospital A and (n=458) in hospital B over seven months (February to August 2012). Interrater reliability measured using 90 records from hospitals A and B (excluded from the final sample) | Patients aged 18 or older, with a hospital stay of at least 24 hours; Psychiatric patients excluded. Males n=460 and females n=692 | The IHI-GTT (34 triggers) including four modules: general care, medication, intensive care, and emergency department. Surgical and perinatal modules excluded | Two-stage review. First stage: three physicians and a quality improvement specialist reviewed medical records (20-minute time limit). Second stage: two traditional Korean medicine professors validated the AEs | % of patients with ≥1 AE was 10.6% (n=122)  7.39 AEs per 1000 patient days  14.5 AEs per 100 admissions | Not reported | Not reported |
| Kennerly et al. (2013), USA (42) | To measure the prevalence of AEs using the IHI-GTT | Retrospective medical record review | 8 non-academic hospitals | N=16,172; Random sample of medical records (n=10 to 35 depending on hospital size) over 48 months (January 2007 to December 2010) | Patients aged 18 or older, with a hospital stay of at least three days, discharge summary and all coding completed; Patients admitted for hospice care, psychiatric or addictive diseases or rehabilitation excluded. Sex not recorded | The IHI-GTT (53 triggers) was implemented and adapted in 2006 by Baylor Health Care System | Two-stage review. First stage: four trained nurses from an external company reviewed medical records (20-minute time limit). Second stage: the project manager, two nurses, and a consultant physician validated the AEs | % of medical records with ≥1 AE was 17.1% (n=2,425)  6.4 and 27.1 AEs per 100 discharges with a length of stay < 3 days and ≥ three days, respectively  23.2 AEs per 100 admissions ≥ 3 days and 5.5 < 3 days in 2008 | Not reported | Not reported |
| Kennerly et al. (2014), USA (43) | To measure the prevalence of AEs using the IHI-GTT | Retrospective medical record review (electronic medical records) | 8 non-academic hospitals | N=9,017; Random sample of medical records (n=10 to 35 depending on hospital size) over 60 months (2007 to 2011) | Patients aged 18 or older, with a hospital stay of at least 3 days, discharge summary and all coding completed; Patients admitted for hospice care, psychiatric or addictive diseases or rehabilitation excluded. Sex not recorded | The IHI-GTT (53 triggers) was implemented and adapted in 2006 by Baylor Health Care System | Two-stage review. First stage: professional nurses reviewed medical records. (20-minute time limit). Second stage: the patient safety office validated the AEs | % of patients with ≥1 AE was 32.1% (n=2,894)  61.4 AEs per 1000 patient days  38.1 AEs per 100 admissions | Not reported | Not reported |
| Kobayashi et al. (2008), Japan (44) | To compare whether voluntary reported AEs can be identified using a retrospective medical record review | Retrospective medical record review | One academic hospital (acute care) | N=200; Random sample of medical records (n=100) from accident report AEs and (n=100) from patients discharged over 12 months (January to December 2002) | Patients aged 0-92 years included. Sex not recorded | The HMPS (18 triggers) | Two-stage review. First stage: Review A: one nurse leader reviewed medical records. Review B: a group of trained nurses reviewed the same medical records. Second stage: a physician review team validated the AEs | % of patients with ≥1 AE was 28.0% (n=28).  Review A identified 89.3% (n=25) AEs, and Review B identified 85.7% (n=24) | Not reported | Not reported |
| Kurutkan et al. (2015), Turkey (45) | To measure the prevalence of AEs using the IHI-GTT | Retrospective medical record review | One academic hospital | N = 229; Random sample of medical records (n=20) over 12 months (September 2012 to August 2013) | Patients aged 18 or older, with a hospital stay of at least 24 hours; Psychiatric patients excluded. Males n=118 and females n=111 | IHI -GTT (53 triggers) | Two-stage review. One review team (three nurses, two pharmacists, one quality management director, and two secretaries). First stage: each medical record was reviewed by three team members (20 minutes time limit). Second stage: two members of the same team validated the AEs | % of patients with ≥1 AE was 13.9% (n=32)  80.72 AEs per 1000 patient days  29.39 AEs per 100 admissions | Not reported.  The GTT was reported to be 19 times more sensitive than the voluntary notification system | Not reported |
| Letaief et al. (2010), Tunisia (46) | To measure the prevalence of AEs in hospitalised patients | Retrospective medical record review | One academic hospital | N=620; Random sample of medical records over 12 months (January to December 2005) | Hospitalised patients with no age or length stay restriction. Males n=289 and females n=331 | HMPS-18 triggers | Two-stage review. First stage: a medical student reviewed medical records. Second stage: Two expert physicians validated the AEs | % of patients with ≥1 AE was  10.0% (n=62) | Not reported | Not reported |
| Lima-Junior et al. (2023), Brazil (47) | To measure the prevalence of preventable AEs | Retrospective medical record review | Two hospitals (mixed hospitals) | N=370; Random sample o medical records over 12 months. (January to December 2015) | Patients aged 18 or older. Psychiatric, obstetrics and palliative care excluded. Sex not recorded | HMPS 19 triggers | Two-stage review. First stage: two nurses reviewed medical records. Second stage: two physicians validated the AEs | % of patients with ≥1 AE was  15.7% (n=58) | Not reported | Not reported |
| Lipitz-Snyderman et al. (2017), USA (48) | To measure the prevalence of AEs with a new developed trigger tool for oncology patients | Retrospective medical records review | One cancer academic hospital | N=400; Stratified random sample of patients with breast (n=128), colorectal (n=136), and lung cancer (n=136) over 12 months (January to December 2012) | Patients 18 or older, with breast, colorectal or lung cancer. Males= 128 and females=272 | Developed a new Oncology trigger tool (49 triggers) | Two-stage review. First stage: Five trained nurses independently review medical records (60-minute time limit). Second stage: Two physicians validated the AEs | % of patients with ≥1 AE was not recorded.  Patients presented 1.98 (n=790) triggers | Sensitivity=92%, and PPV=48%. PPV for individual triggers ranged from 6% (1 trigger) to 100% (5 triggers)(49) | <7% low predictive value for the triggers and >47% to 88% high predictive value for the triggers |
| Magnéli et al. (2019), Sweden (50) | To measure AEs after hip arthroplasty using the IHI-GTT | Cohort study with retrospective medical record review (electronic medical records) | 24 mixed hospitals | N=1,998; Calculated sample medical records over 36 months (January 2009 to December 2011) | Patients aged 18 or older, with either a hemi or total hip arthroplasty, acute and elective surgery from the admission date up to 90 days after surgery; AEs unrelated to the admission and AEs originating from the care of another AE, excluded. Males n=748 and females n=1,250 | IHI-GTT (Swedish version: 38 triggers) divided into 5 modules: general care (18), laboratory (5), surgical (7), medication (3), and intensive care (5) | Two-stage review. First stage: five registered nurses, one operating room nurse, two medical students, one resident orthopaedic surgeon, and four physicians reviewed medical records. Second stage: three physicians or two expert registered nurses validated the AEs | % of patients with ≥1 AE was 58.6% (n=1171) | Sensitivity = 5.7% and 14.8% for 30 and 90 days respectively Specificity = 95.2% and 92.1% for 30 and 90 days respectively | Not reported |
| Mattsson et al. (2014), Denmark (51) | To identify the prevalence of AEs by adding an oncology module to the GTT | Retrospective medical record review | One academic hospital | N=240; Random sample medical records over 12 months (January to December 2010) | Patients aged 18 or older, with a hospital stay of at least 24 hours. Incomplete or unavailable medical records excluded. | IHI-GTT 49 triggers (15 from the cares, 12 from the medication, 16 from surgical, 4 from intensive care, and 2 from emergency department). In addition, 17 new triggers were included from an appended oncology module | Two-stage review. First stage: two groups of four nurses independently reviewed medical records (20-minute time limit). Second stage: A Physician validated the AEs | % of patients with ≥1 AE was  21.0% (n=50)  37.4 AEs per 1000 admission days  23.3 AEs per 100 admissions | Not reported | Not reported |
| Mayor et al. (2017), Welsh (52) | To identify the prevalence of AEs with the IHI-GTT | Retrospective medical record review (both electronic and paper based medical records) | Eleven mixed hospitals | N=4,833; Random sample of medical records with the HMPS (n=4,388) and IHI-GTT (n=4,833) over 36 months (January 2011 to December 2013) | Patients aged 18 or older, with a hospital stay of at least 24 hours; Psychiatric and obstetrics patients excluded. Sex not recorded | IHI-GTT (54 triggers) and the HMPS (18 screening criteria) | Two-stage review. The HMPS First stage: clinical research nurses (n=24) and assistants (n=2) reviewed medical records (no-time limit). Second stage: Experienced physicians (n=12) validated the AEs  The IHI-GTT method implemented according to the UK protocol with (20-minute time limit). The total number of reviewers not recorded. | % of patients with ≥1 AE was  10.3% (n=450) with the HMPS and 9.0% (n=430) with the IHI-GTT | Not reported | Not reported |
| Menéndez-Fraga et al. (2021), Spain (53) | To measure the prevalence of AEs using the IHI-Nursing Facility Trigger Tool | Retrospective medical record review (electronic medical records) | One academic hospital | N=240; Random sample of medical records (n=20) over 12 months (January to December 2017) | Patients aged 18 or older, with a hospital stay of at least 24 hours, discharge summary, and all medical information complete. Sex not recorded | The IHI-Skilled Nursing Facility Trigger Tool has three modules: general care, medication, and resident care | Two-stage review. One nurse and one physician with more than 13 years’ experience using the GTT. First stage: one nurse reviewed medical records. Second stage: One physician validated the AEs. | % of patients with ≥1 AE was  44.6% (n=107)  49.8 AEs per 1000 admission days  57.1 AEs per 100 admissions | Not reported | Not reported |
| Merten et al. (2013), Dutch (54) | To measure the prevalence of AEs in hospitalised older patients | Retrospective medical record review | 21 Dutch hospitals. (Mixed hospitals) | N= 7,917; Random stratified representative sample of Dutch hospitals over 14 months period (August 2005 to October 2006) | Patients with a hospital stay of at least 24 hours; Psychiatry, obstetrics, and children under the age of 1 year excluded. n= 4,744 records of patients 65 years and older, and n=3,173 of patients younger than 65. Sex not recorded | The Harvard Medical Practice Study method (18 triggers) | Three-stage review. First stage:  66 trained nurses independently reviewed medical records. Second stage: at least two of the 55 trained physicians validated each of the AEs. Third stage: Physicians´ disagreements were resolved by a third physician | % of patients with ≥1 AE was 6.9% for patients 65 or older and 4.8% for patients younger than 65 | Not reported | Not reported |
| Mevik et al. (2016), Norway (55) | To measure the prevalence of AEs using the IHI-GTT in a similar hospital large and small sample | Retrospective medical record review | One academic hospital | N=1,920; Random sample of medical records (n=10 and n= 70) fortnightly over 12 months (January to December 2010). Small sample (n=240). Large sample (n=1680) | Patients aged 18 or older, with a hospital stay of at least 24 hours; Psychiatric or rehabilitation patients excluded. Large sample: males n= 638 and females n=1,042 Small sample: Males n= 98 and females n=142 | The IHI-GTT (Norwegian version) | Two-stage review. Large sample: First stage: a nurse or one of two physicians independently reviewed medical records. Second stage: in consensus, the team of one nurse and two physicians validated the AEs. Small sample: First stage: two nurses independently reviewed medical records. Second stage: a physician validated the AEs. | % of patients with ≥1 AE was 20.7% (n=347) and 12.5% (n=30) for the large and small sample respectively  39.3 and 27.2  AEs per 1000 patient days for the large and small sample respectively  26.6 and 18.8 AEs per 100 admissions for the large and small sample respectively | Not reported | Not reported |
| Mevik et al. (2019), Norway (56) | To measure the incidence of AEs using the original IHI-GTT and modified electronic version of the IHI-GTT | Retrospective medical record review and electronic identification of AEs (hybrid medical records: electronic and hand-written) | One medium size academic hospital | N=1,233; Random sample of medical records over 10 months (March to December 2013) | Patients aged 18 or older, with a hospital stay of at least 24 hours; Psychiatric and rehabilitation patients, excluded. Males n=517 and females n=716 | IHI-GTT (Norwegian version: 57 triggers). The automatic trigger identification system excluded 15 triggers (nine for handwritten and scanned into the electronic health records, three labelled ‘other’, and three rarely identified in a previous manual review | Two-stage review. First stage: two nurses independently reviewed medical records (20-minute time limit). Second stage: a physician validated the AEs (No time constraint but an average of 6 minutes per record) | % of patients with ≥1 AE was 15.1% (n=186) with manual IHI-GTT and 15.3% (n=189) with the automatically triggered records  35 AEs per 1000 patient days with manual IHI-GTT and 34.7 with the modified electronic version of the IHI-GTT | The modified electronic version of the IHI-GTT sensitivity = 59%, specificity = 92%, PPV = 58% | Not reported |
| Moraes et al. (2021), Brazil (57) | To measure the prevalence of AEs involving medical students and nurses as primary reviewers using the IHI-GTT | Retrospective medical record review (both electronic and paper-based medical records) | One general public hospital (Academic hospital) | N= 220; Random sample of medical records over one month period (October 4, to November 2, 2016) | Patients aged 18 or older, with a hospital stay of at least 24 hours. Males n=80 and females n=140 | IHI-GTT, Portuguese version | Two-stage review. First stage: two 4th-5th year-medical students reviewed 220 medical records and two experienced nurses reviewed 100 medical records (20 minutes time limit). Second stage: two senior physicians (specialist in Internal Medicine) validated the AEs | % of patients with ≥1 AE was 40.9%  76.1 AEs per 1000 patient days  90.5 AEs per 100 admissions | Not reported | Not reported |
| Mortaro et al. (2021), Italy (58) | To measure the prevalence of AEs using the GTT (Italian version) | Retrospective medical record review | One non-academic (secondary acute care hospital) | N=1,320; Random sample of medical records (n=10 fortnightly) over 66 months (March 2009 to August 2014) | Patients aged 18 or older, with a hospital stay of at least 48 hours, and administrative data completed (including discharge summary); Paediatrics, psychiatry, rehabilitation, and long-term care patients excluded. Males n=523 and females n=797 | IHI-GTT (53 triggers [Italian version]) including original six modules (Four triggers changed to include the brand name of the medication (S8, M8, M7, and P1) | Two-stage review. First stage: two postgraduate students in public health independently reviewed 20 medical records. Second stage: a physician (patient safety officer of the hospital) validated the AEs | % of patients with ≥1 AE was 20.2% (n=267)  30.6 AEs per 1000 patient days  27.7 AEs per 100 admissions | The ten most frequent triggers had a PPV that ranged from 0 to 90% (C1=30.3%, C6=81.1%, C8=77.2%, C10=0%, C11=82%, C13=31.6%, C14=80.4%, M12=53.5%, P4=90%, and E2=1.7%). Six triggers did not identify AEs (M3, M8, M9, S5, P1, and P3) | Not reported |
| Mull et al. (2015), USA (59) | To compare the AEs identified using the IHI-GTT with a voluntary reporting system, patient safety indicators, and the surgical quality improvement program | Retrospective medical record review (electronic medical records) | One large Veterans Health Administration facility (Non-academic) | N= 273; Random sample of medical records (n=20 and n=15) over one and three month period, respectively (July 1 to October 27, 2012) | Patients aged 18 or older, with a hospital stay of at least 24 hours. Discharged at least 30 days prior medical record review. Males n=261 and females n=12 | IHI-GTT (46 of 53 triggers). No obstetric services were offered therefore, the perinatal module was excluded except for the P3 ‘Platelet count less than 50,000’ | Two-stage review. First stage: one nurse reviewed the medical records (20-minute time limit). Second stage: one physician validated the AEs | % of patients with ≥1 AE was 21.6% (n=59)  52 AEs per 1000 patient days  38 AEs per 100 admissions | Of the 109 AEs, 12.0% (n=13) were detected. Voluntary reporting system (n=1), patient safety indicators (n=5), and surgical quality improvement program (n=9). Three AEs were detected by both patient safety indicators and the surgical quality improvement program | Not reported |
| Müller et al. (2016), South Africa (60) | To identify the incidence of AEs in hospitalised patients | Retrospective medical record review (paper based medical records) | One academic hospital | N=160; Random sample of medical records over 8 months (July 2014 to March 2015) | Patients in the internal medicine ward, with a hospital stay of at least 24 hours. Males n=80 and females n=80 | IHI-GTT 28 triggers (15 from the cares and 13 from the medication module) | Two-stage review. First stage: a pharmacist reviewed medical records. Second stage: a clinical pharmacist validated the AEs. Attending physicians were consulted only for clarifying information on medical records (The mean review time was 18.5-minute with a range from 4 to 43 minutes) | % of patients with ≥1 AE was  24.4% (n=39)  25.8 AEs per 1000 admission days  36.9 AEs per 100 admissions | Not reported | Not reported |
| Naessens et al. (2009), USA (61) | To compare the AEs identified using the IHI-GTT with a voluntary reporting system and patient safety indicators | Retrospective medical record review | One academic hospital | N=235; Random sample of medical records (n=20) over 12 months (January to December 2005) | Not recorded inclusion or exclusion criteria for the population, only stated that followed the IHI-GTT method. Sex not recorded | The IHI-GTT | Two-stage review. First stage: Qualified nurses review medical records. Second stage: a physician validated the AEs | % of patients with ≥1 AE was  27.7% (n=65) | Of the 65 AEs identified with the IHI-GTT, only 4.7% were identified by another method. Voluntary report 3.8% (n=9) and Patent safety indicators 0.9% (n=2) | Not reported |
| Naessens et al. (2010), USA (62) | To assess the IHI-GTT inter-rater reliability in different hospitals settings | Retrospective medical record review | Three academic hospitals | N=1,138; Random sample of medical records (n=20) over 61 months (August 2004 to March 2008) | Patients 18 or older, complete medic records, including psychiatric, physical rehabilitation, obstetric, general medical, speciality and surgical unit. Paediatric patients excluded. Sex not recorded | The IHI-GTT (55 triggers) | Two-stage review. First stage: two registered nurses independently review medical records (20-minute time limit). Second stage: a Physician validated the AEs | % of patients with ≥1 AE was  27.0% (n=307)  Hospital A 23.1%, hospital B 27.2%, and hospital C 37.9% | PPV or yield for individual triggers vary from 0% to 82.3% for “mechanical ventilation greater than 24 hours post-op” trigger | Not reported |
| Najjar et al. (2013), Palestin (63) | To measure the prevalence of AEs with the IHI-GTT | Retrospective medical record review | Two mixed hospitals | N=640; Random sample of medical records (n=320) from each hospital over 4 months (May to August 2009) | Patients aged 18 or older, with a hospital stay of at least 24 hours; Not allowed to review deceased patients’ medical records. Males n=260 and females n=380 | IHI-GTT (53 original triggers, three new triggers included, and three triggers adapted to local settings) | Two-stage review. First stage: Two experienced nurses independently reviewed medical records. Second stage: a physician validated the AEs | % of patients with ≥1 AE was  14.2% (n=91) | Not reported | Not reported |
| Nilsson et al. (2012), Sweden (64) | To measure the incidence of AEs in patients who died in the intensive care unit (ICU) | Retrospective medical record review | One academic (middle-sized hospital) | N=128, medical records over 24 months (January 2007 to December 2008) | Patients aged 18 or older received care in the ICU during the 2007 or 2008; patients who died in the ICU or 96 hours after discharge and cared for at the hospital, and all documentation was available for review. Males n=73 and females n=55 | IHI-GTT  (Swedish version). | Two-stage review. First stage: two intensive care nurses independently reviewed medical records with a time limit of 45 minutes. Second stage: two consultant intensivists validated the AEs | % of patients with ≥1 AE was 19.5% (n=17)  32 AEs per 100 ICU admissions | Not reported | Not reported |
| Nilsson et al. (2018), Sweden (65) | To measure the incidence of AEs over a 4-year period in hospitalised patients using the IHI-GTT | Retrospective medical record review | Mixed hospitals. (63 acute care hospitals in 2013 and 2014, 62 in 2015, and 59 in 2016) | N = 64,917; Random sample of medical records (n=40) university hospitals, (n=30) central county council hospitals, (n=20) county hospitals over 48 months (from 2013 to 2016) | Patients aged 18 or older, with a hospital stay of at least 24 hours; Paediatric and psychiatric patients excluded. Sex recorded as 46.8% men (9,326) in 2013, 46% (8,569) in 2014, 47.1% (6,486) in 2015, and 48% (6,043) in 2016. Males n=30,424 and females n=34,493 | IHI-GTT Swedish version (44 of the 53 original triggers). Ten new triggers were created according to the country setting. | Each hospital had a review team. Two-stage review: First stage: one or two nurses reviewed medical records. Second stage: The team, including at least one physician at the senior level, validated the AEs | % of patients with ≥1 AE was in 2013,13.1% (n=2,610);  In 2014, 11.6%, (n=2,161); In 2015, 10.9% (n=1,501), and in 2016, 11.4% (n=1,435) | Not reported | Not reported |
| Nilsson et al. (2020), Sweden (66) | To measure the prevalence of AEs in psychiatric patients | Cohort study with retrospective medical record review | Psychiatric acute care departments in all Swedish regions | N=2,552; Random sample of medical records over six months (January to June 2017). Hospital care, and corresponding outpatient care reviewed as a continuum | Patients aged 18 or older and discharged from a psychiatric hospital. Males n=1,266 and females n=1,286 | A new trigger tool developed with 36 triggers divided into five modules: treatment (15), drugs (4), coercive treatment (4), medicine (7), and continuity and transition (6) | Two-stages review. Each hospital had its own review team (one or two nurses, other mental healthcare professionals, and at least one physician). First stage: nurses independently reviewed medical records. Second stage: the team, including a physician, validated the AEs | % of patients with ≥1 AE was 17.2% (n=438) | Not reported | Not reported |
| Nwulu et al. (2013), United Kingdom (67) | To identify AEs for over-anticoagulation and opioid overdose using two IHI-GTT triggers | Retrospective medical record review (electronic medical records) | One secondary care hospital (Academic hospital) | N=54,244; Over12 months (January to December 2010) | Patient medical records with a test result of INR ≥ 6 and a preceding prescription for warfarin, and with prescription and administration of naloxone, with a preceding opioid prescription. Sex not recorded | Two triggers of the IHI-GTT medication module (INR ≥ 6 and naloxone use) | Two stage review. First stage: triggers were identified electronically Second stage: review of medical records to identify evidence of over-anticoagulation or/and opioid overdose. Details about the reviewers was not reported | n=14 AEs identified for INR ≥ 6 trigger and n=61 AEs for naloxone trigger | INR ≥ 6 trigger, PPV = 38% (14/37); naloxone trigger PPV = 91% (61/67) | Not reported |
| O´Leary et al. (2013), USA (68) | To compare the incidence of AEs detected using a traditional trigger tool and electronic triggers | Retrospective medical record review (electronic medical records) | One academic hospital | N=250; Random sample of medical records over 12 months (September 2009 to August 2010) | Patients admitted to general medical services; patients admitted under observation status and those cared by either of the two medical record reviewers excluded. Males n=115 and females n=135 | Fifty-one triggers were created based on 33 screening criteria from the HMPS, a study on AEs in Utah and Colorado, and the IHI-GTT | Two-stage review. First stage: two physicians independently reviewed medical records. Second stage: a third physician validated the AEs | % of patients with ≥1 AE was 22.0% (n=54) with traditional trigger tool and 21.0% (n=53) with electronic triggers | PPV for individual triggers raged from 0% to 100%.  Five triggers PPV = 100%, eleven triggers PPV ≥ 50%, 18 triggers PPV ≥8% and < 50%, eight triggers PPV=0%, and eight trigger not identified | Not reported |
| Ock et al. (2015), Korea (69) | To measure the consistency in identifying AEs using medical record review | Retrospective medical record review (Paper based medical records) | One acute-care hospital (type of hospital not reported) | N=96; Random sample of medical records, discharged on three selected dates in 2007. Not recorded specific dates | Psychiatric patients excluded. Sex not recorded | The IHI-GTT (41 triggers) included 13 from the HMPS, 22 triggers from the IHI-GTT, and six from both methods | Two-stage review. First stage: two nurses (with more than five years of experience) independently reviewed medical records. Second stage: two physicians (more than ten years of experience) validated the AEs | % of patients with ≥1 AE was 8.3% (n=8)  The nurses agreed to the necessity for a second stage review in 84.0% of the medical records | Not reported | Not reported |
| Otero et al. (2021), Spain (70) | To measure the adverse drug events (ADEs) using a trigger tool in older patients | Retrospective medical record review | 12 Spanish hospitals. The coordinating hospital was academic | N=720; Random sample of medical records (n=5) from each hospital weekly over 12 weeks (March to June 2017) | Patients aged 65 and over, with a hospital stay of at least 48 hours, with multimorbidity; hospitalisation not in the internal medicine or geriatrics unit, were receiving palliative care or transferred from another clinical unit excluded. Males n= 301 and females n= 419 | Trigger-Chron tool (32 triggers in 5 modules) and high-alert medication. Seven triggers for care, 7 for antidotes/drug treatments, 2 for medication concentrations, 15 for abnormal laboratory values, and one emergency department trigger | Two-stage review. First stage: a clinical pharmacist reviewed medical records. Second stage: the clinical pharmacist validated the ADEs. Treating physician responsible for the resolved discrepancies | % of patients with ≥1 AE was 19.3% (n=139) for high-alert medication | Mean PPV = 29.12% with 25 triggers and PPV = 22.75% with 32 triggers. Fifteen triggers had a PPV >=20%. Six triggers identified 69.8% of ADEs. Seven triggers did not identify ADEs | PPV >20% for individual triggers as good triggers |
| Pandya et al. (2020), India (71) | To measure the prevalence of adverse drugs reactions using the IHI-GTT | Prospective surveillance and medical record review | One academic hospital | N=463; Random sample of medical records over 10 months (August 2018 to May 2019) | Patients aged 18 or older, admitted to the emergency department; outpatient department, and medical record not located excluded. Males n=285 and females n=178 | IHI-GTT using 51 triggers | Two-stage review. First stage: three clinical pharmacologists reviewed medical records (20-minute time limit). Second stage: the clinical pharmacologist consulted a physician to validate AEs | % of patients with ≥1 adverse drug reaction = 13.4% (n=62) | Sensitivity = 85.48%, specificity = 54.86%, PPV = 22.65%, NPV = 96.07%.  PPV of 20 triggers ranged from 30 to 100% | PPV >30% for individual triggers as good triggers |
| Paulande et al. (2024), Sweden (72) | To measure the prevalence of AEs in deceased patients who underwent high-risk surgery | Retrospective medical record review | Five academic hospitals | N=60; over 4 months (November 2015 to February 2016) | Patients ages 18 years or older, with a hospital stay of at least 24 hours. Males n=28 and females n=32 | IHI-GTT (Swedish version) | Two-stage review. First stage: at least one physician reviewed medical records. Second stage: Two or three physicians validated the AEs in consensus | % of patients with ≥1 AE was  95% (n=57) | Not reported | Not reported |
| Pérez-Zapata et al. (2017), Spain (73) | To compare the AEs detected using the IHI-GTT and the hospital national registry | Retrospective medical record review (electronic medical records) | One tertiary hospital. Surgical Department (Academic hospital) | N=350; Random sample of medical records over 12-month period (January to December 2012) | Patients aged 18 or older, with a completed clinical record, with urgent or elective admission to the general surgery department. Psychiatric, rehabilitation, patients transferred from another hospital, and transplant patients excluded. Sex not recorded | IHI-GTT (39 of 53 triggers), care (15 triggers), medication (13 triggers), and surgical (11 triggers) modules that could be detected using the hospital informatics system were selected | Two-stage review. First stage: two trained residents of general surgery independently reviewed medical records. Second stage: a senior physician validated the AEs | % of patients with ≥1 AE was 31.7% (n=111) with IHI-GTT and 11.4% (n=40) with hospital national registry. The IHI-GTT detected 89.9% of AEs in comparison to 28.4% detected using the hospital national registry | Sensitivity = 86%, specificity = 93.6%, negative predictive value = 92%, and positive predictive value = 89%. | Not reported |
| Pérez-Zapata et al. (2022), Spain (74) | To measure the prevalence of AEs with the IHI-GTT and validate the predictive value of the tool | Retrospective medical record review | 31 hospitals (Mixed hospitals) | N=1,132; Random sample of medical records over 9 months. (September 2017 to May 2018) | Patients aged 18 or older, hospitalised who underwent surgery in the surgery department. Psychiatric, transplanted and referred patients excluded. Males n=577 and females n=555 | IHI-GTT (40 triggers in 3 modules cares n=12, medication n=11, surgical n=9, and other triggers literature bases n=6)) | Two-stage review. First stage: at least one reviewer, reviewed medical records. Second stage: at least one reviewer validated the AEs. Two reviewers at least in each hospital were reported | % of patients with ≥1 AE was  31.5% (n=357) | PPV=66.52, NPV=92.48, Sensitivity=86.27%, and Specificity=79.55% | Not reported |
| Pettersson et al. (2020), Sweden (75) | To identify the incidence of AEs in patients with femur neck fractures up to 90 days after discharge | Retrospective medical record review (electronic medical records) | One academic hospital | N=163; selected patients over 24 months. Specific dates not recorded | Patients that were autonomous before fracture and with not mental disease that agree to participate in the study; Alcohol or drug abuse patients, with different femur fractures, not able to communicate in the Swedish language excluded. Males n=52 and females n=111 | IHI-GTT (38 triggers) | Two stage review. First stage: a research nurse review medical records (no time limit). Second stage: The same research nurse validated the AEs with option to consult an expert RN in the GTT method, one orthopaedics, and one internal medicine physicians | % of patients with ≥1 AE was  38.0% (n=62) | Not reported | Not reported |
| Pierdevara et al. (2016), Portugal (76) | To measure the prevalence of AEs in an internal medicine department | Retrospective medical record review (electronic medical records) | One academic hospital | N=90; Random sample of medical records over 9 months (January 1 to September 30, 2014) | Patients aged 18 or older, with a hospital stay of at least 24 hours, discharged at least two months before the study commenced, and complete clinical records with a discharge summary. Psychiatric patients excluded. Males n=45 and females n=45 | IHI-GTT (Portuguese version) | Two-stage review. First stage: two nurses independently reviewed medical records (20-minute time limit). Second stage: one physician validated the AEs | % of patients with ≥1 AE was 31.1% (n=28)  62.63 AEs per 1000 patient days  137.8 AEs per 100 admissions | Not reported | Not reported |
| Pierdevara et al. (2020), Portugal (77) | To measure the prevalence of AEs using the IHI-GTT | Retrospective medical records review. A cross-sectional study. | One academic hospital. | N=90; Random sample of medical records over 9 months (August 2017 to April 2018). Medical records included 176 admissions (ranging from 1 to 5 admissions) | Patients aged 18 or older, with a hospital stay of at least 24 hours, complete medical records and administrative procedures, and discharged at least 2 months before medical record review; Psychiatric patients and with illegible medical records excluded. Males n=43 and females n=47 | IHI-GTT (40 triggers): 37 original triggers with three new triggers (two in general care and one in medication modules [C15 ‘sleep disturbance’, C16 ‘skin lesions/maceration’, and M13 ’pain/blood pressure’). The type of infection and complication was included in the respective trigger: C11, ‘healthcare-associated infection’, and C14 ’ complications of a procedure’. Trigger M7, ‘diphenhydramine administration (Benadryl)’, was amended to include “diphenhydramine, cetirizine dihydrochloride, hydroxyzine and clemastine administration’ | Two-stage review. First stage: two nurses independently review medical records (20-minute time limit). Second stage: one physician validated the AEs | % of patients with ≥1 AE was 36.0%; when considering that 46 patients had between two and five admissions, it increases to 66.7%  42.1 AEs per 1000 patient days  157.8 AEs per 100 admissions | The values of the tool were: Sensitivity = 97.8%, specificity = 74.8%, PPV = 69.8%, and NPV = 0.98% | Not reported |
| Resar et al. (2006), USA (78) | To measure the prevalence of AEs in the Intensive Care Unit | Retrospective medical record review | 54 hospitals and 62 ICUs involved (Mixed hospitals) | N=12,074; Random sample of medical records (80 to 500 per hospital) over 46 months (2001 and October 2004) | Patients aged 18 or older with a hospital ICU stay > 48 hours. Sex not recorded | The IHI-Intensive Care Unit trigger tool (23 triggers) | Two-stages review. First stage: two nonphysician reviewers (background information not recorded) independently reviewed medical records (20-minute time limit). Second stage: a physician validated the AEs | % of patients with ≥1 AE was 11.3% (range, 3.2 to 27.3) | Not reported | Not reported |
| Rutberg et al. (2014), Sweden (79) | To measure the prevalence of AEs using the IHI-GTT | Retrospective medical record review (electronic medical records) | One academic hospital | N=960; Random sample of medical records (n=20) over 48 months (January 2009 to December 2012) | Patients aged 18 or older; Paediatric, psychiatric and obstetric patients excluded. Males n=487 and females n=473 | The IHI-GTT (Swedish version) | Two-stage review. First stage: three experienced nurses reviewed medical records (20-minute time limit). Second stage: one of two physicians (senior anaesthesiologist and senior surgeon) with the nurses validated the AEs | % of patients with ≥1 AE was 20.5% (n=197)  33.2 AEs per 1000 patient days  28.2 AEs per 100 admissions | Not reported | Not reported |
| Sajith et al. (2021), Singapore (80) | To measure AEs using a trigger tool in a mental health setting | Retrospective medical record review (both, paper based and electronic medical records) | One academic psychiatric hospital | N= 515; Patients discharged from the psychiatric hospital over six months  (January to June 2014) | Patients aged 21-90 years, with a hospital stay >24 hours and < 90 days and discharged at least one month before the review; Incomplete records and patients admitted to a hospital excluded. Males n=305 and females n=210 | The Mental Health Trigger Tool was developed (25 triggers) with four modules: general care (n=3), laboratory (n=4), medication (n=12), and behaviour (n=6) | Two-stage review. First stage: two pharmacists or nurses independently reviewed medical records (20-minute time limit). Second stage: a physician validated the AEs | % of patients with ≥1 AE was 19.0% (n=98)  13.9 AEs per 1000 patient days | Sensitivity = 98.6% specificity = 100%.  PPV = 33.8%, ranging from 0% to 100% for each trigger | PPV≥20% in 16 triggers and <20% in 8 triggers. |
| Samal et al. (2022), USA (81) | To compare the incidence of AEs using the IHI-GTT and the voluntary reporting system in patients with cancer | Retrospective medical record review (electronic medical records) | One academic hospital (6 oncology units) | N=88; Random sample of medical records (n=4) over 22 months (July 2013 to May 2015) | Patients aged 18 or older, with a hospital stay of at least 24 hours, and admitted to one of the six oncology units. Males n=56 and females n=32 | The IHI-GTT (30 triggers) was modified. Two triggers from the intensive care module were retained, the surgical, perinatal, and emergency department triggers were eliminated | Two-stage review. First stage: one nurse reviewed medical records. Second stage: two physicians validated the AEs. In case of disagreement a third physician resolved the conflict | % of patients with ≥1 AE was 54.5% (n=48) with the modified IHI-GTT and 16.0.% (n=14) with the voluntary reporting system | Not reported | Not reported |
| Sari et al. (2015), Iran (82) | To measure the prevalence of AEs in hospitalised patients | Retrospective medical record review | Four hospitals (Three large and one medium hospitals) | N=1,162; Random sample of medical records over 6 months (April to September 2012) | Patients with a hospital stay of at least 24 hours; Psychiatric excluded. Sex not recorded | HMPS-18 triggers | Two-stage review. First stage: nurses review medical records. Second stage: Two physicians validated the AEs | % of patients with ≥1 AE was  11.0% (n=128)  7.3% (n=85) during hospitalisation and 3.7% (n=43) before hospital admission | Not reported | Not reported |
| Scarpis et al. (2023), Italy (83) | To measure the prevalence of AEs | Retrospective medical record review | One academic hospital (Paper based medical records) | N=291; Random sample of medical records over three months. (July to September 2020) | Patients aged 18 or older, with a hospital stay of at least 24 hours. Psychiatric and rehabilitation patients excluded.  Males n= 92 and females =199 | IHI-GTT 53 triggers (Italian version) | Two-stage review. First stage: a physician reviewed medical records. Second stage: a second physician validated the AEs | % of patients with ≥1 AE was  16.2% (n=47)  25.8 AEs per 1000 admission days  19.2 AEs per 100 admissions | Not reported | Not reported |
| Schildmeijer et al. (2012), Sweden (84) | To measure consistency in the identification of AEs between five GTT teams | Retrospective medical record review | Five hospitals (Not reported if academic, non-academic or mixed) | N=50; Random sample of medical records over eight months (October 2009 to May 2010) | Patients aged 18 or older with a hospital stay of at least 24 hours, surgical, orthopaedic, gynaecology and obstetrics, medical, psychiatry and geriatric clinics, included. Males n=19 and females n=31 | The IHI-GTT (53 triggers: Swedish version) with six different modules (general care, surgical, medication, intensive care, perinatal and emergency department) | Two-stages review. Five teams (one physician and two registered nurses). First stage: two registered nurses independently reviewed medical records. Second stage: a physician validated the AEs | % of patients with ≥1 AE ranged from 14.0% (n=7) to 32.0% (n=16), according to the teams with a mean 20% (n=10)  44.1 AEs per 1000 patient days (range from 27.2 to 99.7) | Not reported | Not reported |
| Schmied et al. (2024), Austria (85) | To measure the prevalence of AEs with the IHI-GTT and the Austrian Inpatient Quality Indicators | Retrospective medical record review | One academic hospital | N=421; Random and non-random sample medical records over 12 months (January to December 2019) | Patients aged 18 or older, with a hospital stay of at least 24 hours. Sex not recorded | IHI-GTT (German version) | Two-stage review. First stage: Two nurses independently reviewed medical records. Second stage: a physician validated the AEs | % of patients with ≥1 AE was  20.2% (n=85) | Not reported | Not reported |
| Sekijima et al. (2020), USA (86) | To measure the incidence of AEs in patients with and without accurate indication of hospitalisation | Cohort study with retrospective medical record review | One Academic hospital | N=300; Systematically sampled of medical records over four months. (February to June 1, 2018). Patients admitted without definite medical acuity (n=150) and (n=150) for the medically appropriate admission | Presentation to the emergency department and admission to the general medicine department. Males n=203 and females n= 97 | The IHI-GTT (28 triggers) in two modules 5 (general care module [15], and medication module [13]) | Two-stage review. First stage: two investigators independently reviewed medical records. Second stage: the same team conducted an in-depth analysis to confirm the AEs. A third investigator was involved to resolve conflicts | % of patients with ≥1 AE was 28.3% (n=85)  73.6 AEs per 1000 patient days  46.3 AEs per 100 admissions | Not reported | Not reported |
| Sharek et al. (2011), USA (87) | To measure the prevalence of AEs using the IHI-GTT with an internal and external review teams | Retrospective medical record review (both electronic and paper based medical records) | Ten acute care hospitals (Mixed hospitals) | N=2,400; Random sample of medical records (n=240) per hospital over 72 months  (January 2002 to December 2007) | Patients 18 or older, with a hospital stay of at least 24 hours; Paediatrics, psychiatric and rehabilitation patients excluded. Males n=857 and females n=1,484 | IHI-GTT | Two stage review. First stage: In each hospital a team of two to four nurses and/or pharmacists review medical records for internal review. Eight reviewers for external review. (20-minute time limit). Second stage; two physicians per hospital independently validated the AEs for internal review and 2 physicians for external review | % of patients with ≥1 AE with the external team was  18.1% (n=429) and 25.1% (n=588) with the internal team | The experienced review team identified 30% of AE, the internal review team 18.8%, and the external review team 15.8% in 202 selected medical records | Not reported |
| Sousa et al. (2014), Portugal (88) | To measure the incidence of AEs in Portuguese hospitals | Cohort study with retrospective medical record review | Three mixed public hospitals | N=1,669; Random sample of medical records over 12 months (January to December 2009) | Patients aged 18 or older, with a hospital stay of at least 24 hours; Psychiatric patients excluded. Sex not recorded | The Harvard Medical Practice Study (18 screening criteria) | Two-stage review. First stage, two nurses in each hospital (with at least five years of experience in clinical audits) reviewed medical records. Second stage: five physicians (one cardiologist, one neurologist, two surgeons, and one internal medicine with at least five years of experience in clinical codes and audits) validated the AEs | % of patients with ≥1 AE was 11.1% (n=186) | Not reported | Not reported |
| Storesund et al. (2019), Norway (89) | To compare the AEs detected by the IHI-GTT and International Classification of Diseases 10^th^ Revision (ICD-10) in surgical patients | Retrospective medical record review (electronic medical records) | Two mixed hospitals. One tertiary teaching hospital and one community hospital | N=700; Random sample of medical records over 29 months (November 2012 to March 2015) | Patients aged 18 or older for surgical admission, with a hospital stay of at least 24 hours; rehabilitation admissions, ambulatory patients, donor surgery and patients who declined to participate, excluded. Males n=309 and females n=391 | IHI-GTT Norwegian version (55 triggers) | Two-stage review. One team in each hospital. First stage: registered nurses (7-35 years of experience) reviewed the medical records. Second stage: one senior anaesthetist and one surgeon validated the AEs | % of patients with ≥1 AE was 30.3% (n=212) with the IHI-GTT  % of patients with ≥1 AEs was 47.4% (n=332) with the ICD-10 (decreased to 20.1% (n=141) when AEs present on admission were excluded) | The agreement between IHI-GTT and ICD-10 was 83.3%  Ninety four AEs identified with the IHI-GTT were not detected with the ICD-10 and 23 AEs detected with ICD-10 were not detected with the IHI-GTT | Not reported |
| Suarez et al. (2014), Spain (90) | To measure the prevalence of AEs using the IHI-GTT in a geriatric hospital | Retrospective medical record review (electronic medical records) | One geriatric academic hospital | N=1,440; Random sample of medical records (n=10, fortnightly) over 72 months (January 2007 to December 2012) | Patients with a hospital stay of at least 24 hours and  outpatients with a significant ambulatory surgery. Sex not recorded | The IHI-GTT (39 triggers) Three modules were used: general care, medication, and surgery | Two-stage review. First stage: two nurses independently reviewed medical records (20-minute time limit). Second stage: a physician validated the AEs | % of patients with ≥1 AE was 23.3% (n=335)  24.5 AEs per 1000 patient days  29.4 AEs per 100 admissions | Not reported | Not reported |
| Thomas et al. (2000), USA (91) | To measure the incidence of AEs in Utah and Colorado | Retrospective medical record review | 28 Mixed hospitals (13 in Utah and 15 in Colorado) | N=14,700; Random sample of medical records from 1992 (n=4,943 in Utah and n=9,757 in Colorado) | Psychiatric, rehabilitation and drug/alcohol hospitals excluded. Males n=5,623 and females n=9,077 | HMPS (18 triggers) | Two-stage review. First stage: a trained nurse reviewed medical records. Second stage: a physician (family practitioners or general internists) validated the AEs or negligent AEs | % of patients with ≥1 AE was 2.9% | Not reported | Not reported |
| Thomas et al. (2002), USA (92) | To measure reliability of physicians in detecting AEs and medical negligence | Retrospective medical record review | Hospitalisations in Utah and Colorado in 1992 | N=500; Random sample of medical records: Non-AEs= (n=400); non-negligent AEs=(n=50) and negligent AEs= (n=50) (167 from Utah and 333 from Colorado hospitals) | Not recorded | The Harvard Medical Practice Study (19 triggers) | Two-stage review. First stage: nurses reviewed medical records. Second stage: Three independent physicians reviewed the 500 medical records and validated the AEs | % of patients with ≥1 AE not recorded.  % of patients with AE =12.8% (n=64) for reviewer 1, 19.0% (n=95) for reviewer 2, and 17.2% (n=86) for reviewer 3 | Not reported. Inter-rater agreement: 7.58% agreement between 3 reviewers, 19.16% between 2 reviewers, and 37.68% agreement for 1 reviewer. | Not reported |
| Toribio-Vicente et al. (2018), Spain (93) | To measure the prevalence of AE in internal medicine and general surgery departments | Retrospective medical record review | One academic hospital | N=233; Random sample of medical records (n=118) internal medicine and general surgery (n=115) over 12 months (January to December 2016) | Hospital stays of at least 24 hours and all medical information complete; ambulatory and short-stay unit patients excluded. Males n=112 and females n=121 | The IHI-GTT. Not recorded the specific triggers or modules used | Two-stage review. First stage: two physicians independently review medical records (no time limit). Second stage: the same physicians validated AEs. A third physician participated as a consultant, and resolved conflicts | % of patients with ≥1 AE was  15.1% (n=13) in internal medicine and 60.7% (n=34) in general surgery  13.1 AEs per 1000 patient day in internal medicine and 39.1 AEs in general surgery | Sensitivity = 80% internal medicine and 90% general surgery,  Specificity = 30% internal medicine and 70% general surgery,  PPV = 20% internal medicine and 60% general surgery, and  NPV = 90% internal medicine and surgery | PPV 20%-60% for the tool. |
| Unbeck et al. (2013), Sweden (94) | To identify AEs with the HMPS and IHI-GTT method | Retrospective medical record review (electronic medical records) | One academic hospital | N=350; Random sample of medical records over 12 months (January to December 2009) | Patients 18 or older, discharged from the orthopaedics department with no restriction on hospital stay; Psychiatric and rehabilitation patients excluded. Males n= and females n=201 | IHI-GTT (53 triggers) and the HMPS (18 screening criteria) | Each method had a review team. Three-stage review. First stage one senior registered nurse review medical records following the HMPS (1-35-minute time limit) and IHI-GTT (20-minute time limit). Second stage; two physicians validated the AEs following the HMPS (1 to 31-minute) and the IHI-GTT (1 to 30-minute). Third stage; medical records with AEs in both methods were compared and analysed | % of patients with ≥1 AE was  30.0% (n=105)  The HMPS identified 29.7% (n=104) and the IHI-GTT 28.0% (n=98) AEs | The PPV=40.3% for the HMPS and PPV=30.4% for the IHI-GTT. Individual PPV for triggers ranged from 0% to 100% | Not reported |
| Val et al. (2020), Spain (95) | To measure the prevalence of AEs with the GTT in two surgical Departments | Retrospective medical record review (electronic medical records) | One academic hospital | N=251; medical records over 12 months (May 2014 to April 2015) | Patients aged 18 or older, with a hospital stay of at least 24 hours, complete medical records, underwent thyroid or parathyroid surgery. All other surgeries excluded. Males n=50 and females n=201 | The IHI-GTT (45 triggers). Thirty-six original GTT triggers in four modules and nine additional triggers included | Two-stage review. One reviewer conducted both stages. Two senior surgeons were consulted to resolve conflicts | % of patients with ≥1 AE was  50.2% (n=126) | Sensitivity = 91.27%, Specificity = 4.8%, PPV = 49.15%, and NPV = 35.29% | Not reported |
| Valencia-Martín et al. (2022), Spain (96) | To measure the prevalence of AEs | Retrospective medical record review | 34 mixed hospitals | N=9975; Random sample of medical records over one month. (May 2019) | All patients hospitalised in a specific day in May 2019 (date not registered), with a hospital stay of at least 24 hours; Patients in emergency units excluded. Males n=4,989 and females n=4,986 | The HMPS | Two-stage review. Each hospital had own review team. First stage: a healthcare worker reviewed medical records. Second stage: a trained physician validated the AEs | % of patients with ≥1 AE was  11.9% (n=1187) | Sensitivity=73.9%, Specificity=11.5%, PPV=37.9%, and NPV=37.8% | Not reported |
| Valkonen et al. (2023), Finland (97) | To measure the prevalence of ADEs in a single hospital in Finland | Retrospective medical record review (electronic Health records) | One academic hospital | N=834; Random sample medical record over 60 months (From January 2017 to December 2021) | Patients aged 18 years or older, with a hospital stay of at least 24 hours. Psychiatric or rehabilitation excluded. Males n=427 and females n=407 | IHI-GTT (Kuopio University Hospital version) | Two-stage review. First stage: two nurses independently reviewed medical records. Second stage: One physician with two nurses validated the AEs in consensus | % of patients with ≥1 AE was  6.4% (n=53) | PPV for Blood glucose <3-5 mmol/L=100%, other medication-related triggers=96% and oversedation/hypotension=47% | Not reported |
| Wilson et al (2012), Egypt, Jordan, Kenya, Morocco, Tunisia, Sudan, South Africa and Yemen (98) | To measure the prevalence of AEs in middle-income countries | Retrospective medical record review | 26 public or private including: teaching, obstetric and paediatric (Mixed hospitals)  Egypt (n=3), Jordan (n=5), Kenya (n=2), Morocco (n=2), South Africa (n=2), Sudan (n=6), Tunisia (n=2), and Yemen (n=4) | N=15,548; Random sample of medical records over 12 months. Egypt (n=1,358), Jordan (n=3,769), Kenya (n=1,938), Morocco (n=954), South Africa (n=931), Sudan (n=3,977), Tunisia (n=930), and Yemen (n=1,661)  (January to December 2005) | Patients admitted to any of the 26 hospitals including medical, surgical, paediatric and obstetric admissions. Males varied from 34% to 53% and females varied from 47% to 66% | HMPS-18 triggers | Two-stage review. First stage: nurses or junior doctors review medical records. Second stage: A senior physician validated the AEs | % of patients with ≥1 AE was  8.2% (n=1,277)  Egypt 6.0% (n=81), Jordan 2.5% (n=93), Kenya 14.5% (n=281), Morocco 14.8% (n=146), South Africa 8.2% (n=76), Sudan 5.5% (n=218), Tunisia 8.3% (n=77), and Yemen 18.3% (n=305) | Not reported | Not reported |
| Wong et al. (2015), Canada (99) | To measure the incidence of AEs using the IHI-GTT and with a real-time observer in a medical ward | Prospective clinical surveillance (electronic medical records) | One academic hospital | N=141; medical records over 4 months (November 2010 to February 2011) | All patients admitted to a general medicine unit during the study period. Males n=55 and females n=86 | IHI-GTT 57 triggers (29 original triggers and 28 new triggers added) | Two-stage process. First stage: An advanced practice nurse reviewed medical records and asked front- line staff in near-real-time in case of positive triggers. Second stage: A team (an advanced practice nurse, one pharmacist, and one geriatric medicine physician) reviewed the cases to identify preventable and potential AEs | % of patients with ≥1 AE was 12.0% (n=17)  31 AEs per 1000 patient days | Not reported | Not reported |
| Xu et al. (2020), China (100) | To measure the prevalence of AEs using the IHI-GTT in a Chinese general hospital | Retrospective medical record review (electronic medical records) | One tertiary general hospital (Academic hospital) | N=240; Random sample of medical records (n=20) over 12 months (January to December 2014) | Patients aged 18 or older, with a hospital stay of at least 24 hours. Neonatology, paediatrics, rehabilitation and elderly care excluded. Psychiatric hospital care not offered. Males n=91 and females n=149 | 51 triggers (43 from the original IHI-GTT and eight new triggers). Some triggers were omitted or modified in five modules (excluding the emergency module) | Two-stage review. First stage: two pharmacists independently reviewed medical records (20-minute time limit). Second stage: The head of the pharmacy department, four pharmacists, and one senior physician validated the AEs | % of patients with ≥1 AE was 22.5% (n=54)  32.1 AEs per 1000 patient days  29.2 AEs per 100 admissions | Not reported | Not reported |
| Zadvinskis et al. (2018), USA (49) | To measure the AEs using a modified IHI-GTT | Retrospective medical record review (electronic medical records) | One academic hospital, 32 nursing units/departments | N=317; Random sample of medical records (n=10) per unit/department over 1 month (January 1-31, 2013) | Patients aged 18 or older, with a hospital stay of at least 24 hours, complete medical record, with completed discharge and coding summary;  Psychiatric, addictive disease, or rehabilitation admissions excluded. Males n=135 and females n=182 | IHI-GTT modification for unit-level care (according to the care provided in each unit). Total number of triggers modified not recorded. | Two-stages review. First stage: one reviewer examined 317 medical records. Second stage: 32 records were validated to detect AEs by a second reviewer. Both reviewers discussed 23 complicated cases to resolve conflict | % of patients with ≥1 AE was 20.0%  69 AEs per 1000 patient days  21 AEs per 100 admissions | Six triggers had a PPV greater than 75%. 1) Hospital-acquired infection 100%, 2) injury, repair or removal of organ 100%, 3) oversedation/hypotension 100%, 4) any procedure complication 90%, 5) antiemetic use 84%, and 6) surgical complications 75%. Diphenhydramine 13% was not significantly predictive of detecting AEs | Individual triggers with PPV<13% not very predictive and >75% very predictive |

*Academic hospital = academic hospital, teaching hospital, and university hospital

*ADEs = Adverse drug events

*AEs = Adverse events

*EMR = Electronic medical records

*HMPS = Harvard Medical Practice Study

*IHI-ADE = Institute for Healthcare Improvement Adverse drug event trigger tool

*IHI-GTT = Institute for Healthcare Improvement Global Trigger Tool method.

* HMPS = Harvard Medical Practice -study

*ICU = Intensive care unit

*Medical record review = Medical record review and medical chart review

*Mixed Hospitals = Include academic and non-academic hospitals

**References**

1. Aibar L, Rabanaque MJ, Aibar C, Aranaz JM, Mozas J. Patient safety and adverse events related with obstetric care. Archives of gynecology and obstetrics. 2015;291(4):825-30.

2. Aikawa G, Sakuramoto H, Ouchi A, Ono C, Hoshino T, Kido T, et al. Development of the Japanese version of the Intensive Care Unit Trigger Tool to detect adverse events in critically ill patients. Acute Medicine and Surgery. 2021;8(1).

3. Ali S, Peterson GM, Curtain CM, Wilson A, Salahudeen MS. Adverse Drug Event–Related Hospital Admissions among Australian Aged Care Residents: A Cross-Sectional Study. Journal of the American Medical Directors Association. 2024;25(7):N.PAG-N.PAG.

4. Asavaroengchai S, Sriratanaban J, Hiransuthikul N, Supachutikul A. Identifying adverse events in hospitalized patients using Global Trigger Tool in Thailand. Asian Biomedicine. 2009;3(5):545-50.

5. Baker GR, Norton PG, Flintoft V, Blais R, Brown A, Cox J, et al. The Canadian Adverse Events Study: the incidence of adverse events among hospital patients in Canada. Cmaj. 2004;170(11):1678-86.

6. Bates DW, Levine DM, Salmasian H, Syrowatka A, Shahian DM, Lipsitz S, et al. The Safety of Inpatient Health Care. New England Journal of Medicine. 2023;388(2):142-53.

7. Bjertnaes O, Deilkås ET, Skudal KE, Iversen HH, Bjerkan AM. The association between patient-reported incidents in hospitals and estimated rates of patient harm. International Journal for Quality in Health Care. 2015;27(1):26-30.

8. Brennan TA, Leape LL, Laird NM, Hebert L, Localio AR, Lawthers AG, et al. Incidence of adverse events and negligence in hospitalized patients: results of the Harvard Medical Practice Study I. 1991. Quality & safety in health care. 2004;13(2):145-51; discussion 51-52.

9. Brosterhaus M, Hammer A, Kalina S, Grau S, Roeth AA, Ashmawy H, et al. Applying the Global Trigger Tool in German Hospitals: a Pilot in Surgery and Neurosurgery. Journal of patient safety. 2020;16(4):e340‐e51.

10. Brown SK, Peterson J, Schiedel SH, Janes KV. Evaluation of Trigger Tool Methodology Related to Adverse Drug Events in Hospitalized Patients. Patient Safety (2689-0143). 2019;1(2):14-23.

11. Carnevali L, Krug B, Amant F, Van Pee D, Gérard V, de Béthune X, et al. Performance of the adverse drug event trigger tool and the global trigger tool for identifying adverse drug events: experience in a Belgian hospital. The Annals of pharmacotherapy. 2013;47(11):1414-9.

12. Classen DC, Lloyd RC, Provost L, Griffin FA, Resar R. Development and evaluation of the institute for healthcare improvement global trigger tool. Journal of Patient Safety. 2008;4(3):169-77.

13. Classen DC, Resar R, Griffin F, Federico F, Frankel T, Kimmel N, et al. ‘Global trigger tool’shows that adverse events in hospitals may be ten times greater than previously measured. Health affairs. 2011;30(4):581-9.

14. Cohen MM, Kimmel NL, Benage MK, Cox MJ, Sanders N, Spence D, et al. Medication safety program reduces adverse drug events in a community hospital. Quality & Safety in Health Care. 2005:169-74.

15. Connolly W, Rafter N, Conroy RM, Stuart C, Hickey A, Williams DJ. The Irish National Adverse Event Study-2 (INAES-2): longitudinal trends in adverse event rates in the Irish healthcare system. BMJ Quality & Safety. 2021;30(7):547-58.

16. Croft LD, Liquori ME, Ladd J, Day HR, Pineles L, Lamos EM, et al. Frequency of adverse events before, during, and after hospital admission. Southern Medical Journal. 2016;109(10):631-5.

17. Davis P, Lay-Yee R, Briant R, Ali W, Scott A, Schug S. Adverse events in New Zealand public hospitals I: occurrence and impact. The New Zealand Medical Journal (Online). 2002;115(1167).

18. Deilkås ET, Bukholm G, Lindstrøm JC, Haugen M. Monitoring adverse events in Norwegian hospitals from 2010 to 2013. BMJ open. 2015;5(12):e008576.

19. Deilkås ET, Risberg MB, Haugen M, Lindstrøm JC, Nylén U, Rutberg H, et al. Exploring similarities and differences in hospital adverse event rates between Norway and Sweden using Global Trigger Tool. BMJ Open. 2017;7(3).

20. Deilkås ET, Haugen M, Risberg MB, Narbuvold H, Flesland Ø, Nylén U, et al. Longitudinal rates of hospital adverse events that contributed to death in Norway and Sweden from 2013 to 2018. Journal of Patient Safety and Risk Management. 2021;26(4):153-60.

21. Dolci E, Schärer B, Grossmann N, Musy SN, Zúñiga F, Bachnick S, et al. Automated fall detection algorithm with global trigger tool, incident reports, manual chart review, and patient-reported falls: Algorithm development and validation with a retrospective diagnostic accuracy study. Journal of Medical Internet Research. 2020;22(9).

22. Dotta AT, Duarte Sotelo LE, Biaggioni MA, Martín SV, de Tapia JB, Encina R, et al. Detección de eventos adversos en pacientes internados en clínica médica utilizando la herramienta Global Trigger Tool. Medicina (Buenos Aires). 2024;84(1):87-95.

23. El Saghir A, Dimitriou G, Scholer M, Istampoulouoglou I, Heinrich P, Baumgartl K, et al. Development and Implementation of an e-Trigger Tool for Adverse Drug Events in a Swiss University Hospital. Drug, Healthcare and Patient Safety. 2021;13:251-63.

24. Fajreldines A, Pellizzari M, Valerio M, Rodriguez V. Eventos adversos asociados al cuidado de la salud en adultos internados en dos hospitales de alta complejidad de Argentina. MEDICINA (Buenos Aires). 2022;82(3).

25. Franklin BD, Birch S, Savage I, Wong I, Woloshynowych M, Jacklin A, et al. Methodological variability in detecting prescribing errors and consequences for the evaluation of interventions. Pharmacoepidemiology and drug safety. 2009;18(11):992-9.

26. Franklin BD, Birch S, Schachter M, Barber N. Testing a trigger tool as a method of detecting harm from medication errors in a UK hospital: A pilot study. International Journal of Pharmacy Practice. 2010;18(5):305-11.

27. Garrett PR, Jr., Sammer C, Nelson A, Paisley KA, Jones C, Shapiro E, et al. Developing and implementing a standardized process for global trigger tool application across a large health system. Joint Commission journal on quality and patient safety. 2013;39(7):292-7.

28. Gómez López VE, Muñoz Macías C, Casas Cuesta R, Álvarez-Lara MA, Crespo Montero R. Análisis de las medidas correctoras para la disminución de los eventos adversos en una unidad de hemodiálisis hospitalaria. Enfermería Nefrológica. 2019;22(1):27-33.

29. Good VS, Saldaña M, Gilder R, Nicewander D, Kennerly DA. Large-scale deployment of the Global Trigger Tool across a large hospital system: Refinements for the characterisation of adverse events to support patient safety learning opportunities. BMJ Quality and Safety. 2011;20(1):25-30.

30. Griffey RT, Schneider RM, Todorov AA. The Emergency Department Trigger Tool: A Novel Approach to Screening for Quality and Safety Events. Annals of Emergency Medicine. 2020;76(2):230-40.

31. Griffin FA, Classen DC. Detection of adverse events in surgical patients using the Trigger Tool approach. Quality & Safety in Health Care. 2008;17(4):253-8.

32. Grossmann N, Gratwohl F, Musy SN, Nielen NM, Donzé J, Simon M. Describing adverse events in medical inpatients using the Global Trigger Tool. Swiss medical weekly. 2019;149:w20149.

33. Gunningberg L, Sving E, Hommel A, Ålenius C, Wiger P, Bååth C. Tracking pressure injuries as adverse events: National use of the Global Trigger Tool over a 4‐year period. Journal of Evaluation in Clinical Practice. 2019;25(1):21-7.

34. Guzmán-Ruiz O, Ruiz-López P, Gómez-Cámara A, Ramírez-Martín M. Detección de eventos adversos en pacientes adultos hospitalizados mediante el método Global TriggerTool. Revista de calidad asistencial. 2015;30(4):166-74.

35. Härkänen M, Kervinen M, Ahonen J, Voutilainen A, Turunen H, Vehviläinen ‐ Julkunen K. Patient-specific risk factors of adverse drug events in adult inpatients - evidence detected using the Global Trigger Tool method. Journal of Clinical Nursing (John Wiley & Sons, Inc). 2015;24(3-4):582-91.

36. Haukland EC, von Plessen C, Nieder C, Vonen B. Adverse events in hospitalised cancer patients: a comparison to a general hospital population. Acta oncologica (Stockholm, Sweden). 2017;56(9):1218-23.

37. Hommel A, Magneli M, Samuelsson B, Schildmeijer K, Sjostrand D, Goransson KE, et al. Exploring the incidence and nature of nursing-sensitive orthopaedic adverse events: a multicenter cohort study using Global Trigger Tool. International journal of nursing studies. 2020;102:103473‐.

38. Hu Q, Wu B, Zhan M, Jia W, Huang Y, Xu T. Adverse events identified by the global trigger tool at a university hospital: A retrospective medical record review. Journal of Evidence-Based Medicine. 2019;12(2):91-7.

39. Hug BL, Witkowski DJ, Sox CM, Keohane CA, Seger DL, Yoon C, et al. Adverse drug event rates in six community hospitals and the potential impact of computerized physician order entry for prevention. Journal of general internal medicine. 2010;25(1):31-8.

40. Hwang JI, Chin HJ, Chang YS. Characteristics associated with the occurrence of adverse events: a retrospective medical record review using the Global Trigger Tool in a fully digitalized tertiary teaching hospital in K orea. Journal of Evaluation in clinical practice. 2014;20(1):27-35.

41. Hwang J-I, Kim J, Park J-W. Adverse Events in Korean Traditional Medicine Hospitals: A Retrospective Medical Record Review. Journal of patient safety. 2018;14(3):157-63.

42. Kennerly DA, Saldaña M, Kudyakov R, da Graca B, Nicewander D, Compton J. Description and evaluation of adaptations to the global trigger tool to enhance value to adverse event reduction efforts. Journal of patient safety. 2013;9(2):87-95.

43. Kennerly DA, Kudyakov R, da Graca B, Saldaña M, Compton J, Nicewander D, et al. Characterization of adverse events detected in a large health care delivery system using an enhanced global trigger tool over a five‐year interval. Health services research. 2014;49(5):1407-25.

44. Kobayashi M, Ikeda S, Kitazawa N, Sakai H. Validity of retrospective review of medical records as a means of identifying adverse events: comparison between medical records and accident reports. Journal of evaluation in clinical practice. 2008;14(1):126-30.

45. Kurutkan MN, Usta E, Orhan F, Simsekler M. Application of the IHI Global Trigger Tool in measuring the adverse event rate in a Turkish healthcare setting. International Journal of Risk & Safety in Medicine. 2015;27(1):11-21.

46. Letaief M, El Mhamdi S, El-Asady R, Siddiqi S, Abdullatif A. Adverse events in a Tunisian hospital: results of a retrospective cohort study. International journal for quality in health care. 2010;22(5):380-5.

47. Lima Júnior AJd, Zanetti ACB, Dias BM, Bernardes A, Gastaldi FM, Gabriel CS. Occurrence and preventability of adverse events in hospitals: a retrospective study. Revista Brasileira de Enfermagem. 2023;76:e20220025.

48. Lipitz-Snyderman A, Classen D, Pfister D, Killen A, Atoria CL, Fortier E, et al. Performance of a trigger tool for identifying adverse events in oncology. Journal of Oncology Practice. 2017;13(3):e223-e30.

49. Zadvinskis IM, Salsberry PJ, Chipps EM, Patterson ES, Szalacha LA, Crea KA. An Exploration of Contributing Factors to Patient Safety. Journal of Nursing Care Quality. 2018;33(2):108-15.

50. Magnéli M, Unbeck M, Rogmark C, Rolfson O, Hommel A, Samuelsson B, et al. Validation of adverse events after hip arthroplasty: a Swedish multi-centre cohort study. BMJ open. 2019;9(3):e023773.

51. Mattsson TO, Knudsen JL, Brixen K, Herrstedt J. Does adding an appended oncology module to the Global Trigger Tool increase its value? International Journal for Quality in Health Care. 2014;26(5):553-60.

52. Mayor S, Baines E, Vincent C, Lankshear A, Edwards A, Aylward M, et al. Measuring harm and informing quality improvement in the Welsh NHS: the longitudinal Welsh national adverse events study. 2017.

53. Menéndez-Fraga M, Alonso J, Cimadevilla B, Cueto B, Vazquez F. Does Skilled Nursing Facility Trigger Tool used with Global Trigger Tool increase its value for adverse events evaluation? Journal of healthcare quality research. 2021;36(2):75-80.

54. Merten H, Zegers M, de Bruijne MC, Wagner C. Scale, nature, preventability and causes of adverse events in hospitalised older patients. Age and ageing. 2013;42(1):87-93.

55. Mevik K, Griffin FA, Hansen TE, Deilkås ET, Vonen B. Does increasing the size of bi-weekly samples of records influence results when using the Global Trigger Tool? An observational study of retrospective record reviews of two different sample sizes. BMJ open. 2016;6(4):e010700.

56. Mevik K, Hansen TE, Deilkås EC, Ringdal AM, Vonen B. Is a modified Global Trigger Tool method using automatic trigger identification valid when measuring adverse events? International journal for quality in health care : journal of the International Society for Quality in Health Care. 2019;31(7):535-40.

57. Moraes SM, Ferrari TCA, Figueiredo NMP, Almeida TNC, Sampaio CCL, Andrade YCP, et al. Assessment of the reliability of the IHI Global Trigger Tool: new perspectives from a Brazilian study. International journal for quality in health care : journal of the international society for quality in health care. 2021;33(1).

58. Mortaro A, Moretti F, Pascu D, Tessari L, Tardivo S, Pancheri S, et al. Adverse Events Detection Through Global Trigger Tool Methodology: results From a 5-Year Study in an Italian Hospital and Opportunities to Improve Interrater Reliability. Journal of patient safety. 2021;17(6):451‐7.

59. Mull HJ, Brennan CW, Folkes T, Hermos J, Chan J, Rosen AK, et al. Identifying Previously Undetected Harm: Piloting the Institute for Healthcare Improvement's Global Trigger Tool in the Veterans Health Administration. Quality Management in Health Care. 2015;24(3):140-6.

60. Müller MM, Gous A, Schellack N. Measuring adverse events using a trigger tool in a paper based patient information system at a teaching hospital in South Africa. European journal of clinical pharmacy: atención farmacéutica. 2016;18(2):103-12.

61. Naessens JM, Campbell CR, Huddleston JM, Berg BP, Lefante JJ, Williams AR, et al. A comparison of hospital adverse events identified by three widely used detection methods. International Journal for Quality in Health Care. 2009;21(4):301-7.

62. Naessens JM, O'Byrne TJ, Johnson MG, Vansuch MB, McGlone CM, Huddleston JM. Measuring hospital adverse events: assessing inter-rater reliability and trigger performance of the Global Trigger Tool. International Journal for Quality in Health Care. 2010;22(4):266-74.

63. Najjar S, Hamdan M, Euwema MC, Vleugels A, Sermeus W, Massoud R, et al. The Global Trigger Tool shows that one out of seven patients suffers harm in Palestinian hospitals: challenges for launching a strategic safety plan. International journal for quality in health care. 2013;25(6):640-7.

64. Nilsson L, Pihl A, Tågsjö M, Ericsson E. Adverse events are common on the intensive care unit: results from a structured record review. Acta anaesthesiologica Scandinavica. 2012;56(8):959-65.

65. Nilsson L, Borgstedt-Risberg M, Soop M, Nylén U, Ålenius C, Rutberg H. Incidence of adverse events in Sweden during 2013-2016: a cohort study describing the implementation of a national trigger tool. BMJ open. 2018;8(3):e020833.

66. Nilsson L, Borgstedt-Risberg M, Brunner C, Nyberg U, Nylén U, Ålenius C, et al. Adverse events in psychiatry: A national cohort study in Sweden with a unique psychiatric trigger tool. BMC Psychiatry. 2020;20(1).

67. Nwulu U, Nirantharakumar K, Odesanya R, McDowell S, Coleman J. Improvement in the detection of adverse drug events by the use of electronic health and prescription records: An evaluation of two trigger tools. European Journal of Clinical Pharmacology. 2013;69(2):255-9.

68. O'Leary KJ, Devisetty VK, Patel AR, Malkenson D, Sama P, Thompson WK, et al. Comparison of traditional trigger tool to data warehouse based screening for identifying hospital adverse events. BMJ Quality and Safety. 2013;22(2):130-8.

69. Ock M, Lee S-i, Jo M-W, Lee JY, Kim S-H. Assessing Reliability of Medical Record Reviews for the Detection of Hospital Adverse Events. Journal of preventive medicine and public health = Yebang Uihakhoe chi. 2015;48(5):239-48.

70. Otero MJ, Toscano Guzmán MD, Galván-Banqueri M, Martinez-Sotelo J, Santos-Rubio MD. Utility of a trigger tool (TRIGGER-CHRON) to detect adverse events associated with high-alert medications in patients with multimorbidity. European Journal of Hospital Pharmacy. 2021;28(e1):E41-E6.

71. Pandya AD, Patel K, Rana D, Gupta SD, Malhotra SD, Patel P. Global Trigger Tool: Proficient Adverse Drug Reaction Autodetection Method in Critical Care Patient Units. Indian Journal of Critical Care Medicine. 2020;24(3):172-8.

72. Paulander J, Ahlstrand R, Bartha E, Nilsson L, Rakosi K, Sandblom G, et al. Events preceding death after high-risk surgery analyzed by Global Trigger Tool and reflective-thematic approach. Acta Anaesthesiologica Scandinavica. 2024;68(10):1481-6.

73. Pérez Zapata AI, Gutiérrez Samaniego M, Rodríguez Cuéllar E, Gómez de la Cámara A, Ruiz López P. [Comparison of the "Trigger" tool with the minimum basic data set for detecting adverse events in general surgery]. Revista de calidad asistencial : organo de la Sociedad Espanola de Calidad Asistencial. 2017;32(4):209-14.

74. Pérez Zapata AI, Rodríguez Cuéllar E, de la Fuente Bartolomé M, Martín-Arriscado Arroba C, García Morales MT, Loinaz Segurola C, et al. Predictive Power of the" Trigger Tool" for the detection of adverse events in general surgery: a multicenter observational validation study. Patient safety in surgery. 2022;16(1):7.

75. Pettersson PK, Sköldenberg O, Samuelsson B, Stark A, Muren O, Unbeck M. The identification of adverse events in hip fracture patients using the global trigger tool: A prospective observational cohort study. International Journal of Orthopaedic and Trauma Nursing. 2020;38:100779.

76. Pierdevara L, Ventura IM, Eiras M, Brito Gracias AM, Soares da Silva C. An experience with the Global Trigger Tool for the study of adverse events in a medical ward. Revista de Enfermagem Referência. 2016;4(9):97-105.

77. Pierdevara L, Porcel-Gálvez AM, Maria A, da Silva F, Trigo SB, Eiras M. Translation, cross-cultural adaptation, and measurement properties of the portuguese version of the global trigger tool for adverse events. Therapeutics and Clinical Risk Management. 2020;16:1175-83.

78. Resar RK, Rozich JD, Simmonds T, Haraden CR. Methods, tools, and strategies. A trigger tool to identify adverse events in the intensive care unit. Joint Commission Journal on Quality & Patient Safety. 2006;32(10):585-90.

79. Rutberg H, Risberg MB, Sjödahl R, Nordqvist P, Valter L, Nilsson L. Characterisations of adverse events detected in a university hospital: a 4-year study using the Global Trigger Tool method. BMJ open. 2014;4(5):e004879.

80. Sajith SG, Fung DSS, Chua HC. The Mental Health Trigger Tool: Development and Testing of a Specialized Trigger Tool for Mental Health Settings. Journal of Patient Safety. 2021;17(4):e360-e6.

81. Samal L, Khasnabish S, Foskett C, Zigmont K, Faxvaag A, Chang F, et al. Comparison of a Voluntary Safety Reporting System to a Global Trigger Tool for Identifying Adverse Events in an Oncology Population. Journal of Patient Safety. 2022;18(6):611-6.

82. Sari AA, Doshmangir L, Torabi F, Rashidian A, Sedaghat M, Ghomi R, et al. The incidence, nature and consequences of adverse events in Iranian hospitals. Archives of Iranian medicine. 2015;18(12):0-.

83. Scarpis E, Cautero P, Tullio A, Mellace F, Farneti F, Londero C, et al. Are adverse events related to the completeness of clinical records? Results from a retrospective records review using the Global Trigger Tool. International Journal for Quality in Health Care. 2023;35(4):mzad094.

84. Schildmeijer K, Nilsson L, Årestedt K, Perk J. Assessment of adverse events in medical care: Lack of consistency between experienced teams using the global trigger tool. BMJ Quality and Safety. 2012;21(4):307-14.

85. Schmied M, Buchberger W, Perkhofer D, Kvitsaridze I, Brunner W, Kapferer O, et al. Detection of Adverse Events With the Austrian Inpatient Quality Indicators. Journal of Patient Safety. 2023:10.1097.

86. Sekijima A, Sunga C, Bann M. Adverse Events Experienced by Patients Hospitalized without Definite Medical Acuity: A Retrospective Cohort Study. Journal of hospital medicine. 2020;15(1):42-5.

87. Sharek PJ, Parry G, Goldmann D, Bones K, Hackbarth A, Resar R, et al. Performance characteristics of a methodology to quantify adverse events over time in hospitalized patients. Health Services Research. 2011;46(2):654-78.

88. Sousa P, Uva AS, Serranheira F, Nunes C, Leite ES. Estimating the incidence of adverse events in Portuguese hospitals: a contribution to improving quality and patient safety. BMC health services research. 2014;14:311.

89. Storesund A, Haugen AS, Hjortås M, Nortvedt MW, Flaatten H, Eide GE, et al. Accuracy of surgical complication rate estimation using ICD-10 codes. British Journal of Surgery. 2019;106(3):236-44.

90. Suarez C, Menendez MD, Alonso J, Castaño N, Alonso M, Vazquez F. Detection of Adverse Events in an Acute Geriatric Hospital over a 6-Year Period Using the Global Trigger Tool. Journal of the American Geriatrics Society. 2014;62(5):896-900.

91. Thomas EJ, Studdert DM, Burstin HR, Orav EJ, Zeena T, Williams EJ, et al. Incidence and types of adverse events and negligent care in Utah and Colorado. Medical care. 2000:261-71.

92. Thomas EJ, Lipsitz SR, Studdert DM, Brennan TA. The reliability of medical record review for estimating adverse event rates. Annals of internal medicine. 2002;136(11):812-6.

93. Toribio-Vicente MJ, Chalco-Orrego JP, Díaz-Redondo A, Llorente-Parrado C, Plá-Mestre R. Detección de eventos adversos mediante trigger tools en 2 unidades de hospitalización de un hospital terciario en España. Journal of Healthcare Quality Research. 2018;33(4):199-205.

94. Unbeck M, Schildmeijer K, Henriksson P, Jürgensen U, Muren O, Nilsson L, et al. Is detection of adverse events affected by record review methodology? an evaluation of the “Harvard Medical Practice Study” method and the “Global Trigger Tool”. Patient safety in surgery. 2013;7(1):1-12.

95. Val RK, López PR, Zapata AP, de la Cámara AG, de la Cruz Vigo F. Detección de eventos adversos en la cirugía tiroidea y paratiroidea utilizando la herramienta trigger y el Conjunto Mínimo de Datos Básicos (CMBD). Journal of Healthcare Quality Research. 2020;35(6):348-54.

96. Valencia‐Martín JL, Vicente‐Guijarro J, San Jose‐Saras D, Moreno‐Nunez P, Pardo‐Hernández A, Aranaz‐Andrés JM, et al. Prevalence, characteristics, and impact of Adverse Events in 34 Madrid hospitals. The ESHMAD study. European Journal of Clinical Investigation. 2022:e13851.

97. Valkonen V, Haatainen K, Saano S, Tiihonen M. Evaluation of Global trigger tool as a medication safety tool for adverse drug event detection—a cross-sectional study in a tertiary hospital. European Journal of Clinical Pharmacology. 2023;79(5):617-25.

98. Wilson RM, Michel P, Olsen S, Gibberd R, Vincent C, El-Assady R, et al. Patient safety in developing countries: retrospective estimation of scale and nature of harm to patients in hospital. Bmj. 2012;344.

99. Wong BM, Dyal S, Etchells EE, Knowles S, Gerard L, Diamantouros A, et al. Application of a trigger tool in near real time to inform quality improvement activities: a prospective study in a general medicine ward. BMJ quality & safety. 2015;24(4):272-81.

100. Xu X-D, Yuan Y-J, Zhao L-M, Li Y, Zhang H-Z, Wu H. Adverse Events at Baseline in a Chinese General Hospital: A Pilot Study of the Global Trigger Tool. Journal of Patient Safety. 2020;16(4):269-73.
