# Supplementary material for: Predictive values of trigger tools for identifying adverse events in hospitalized patients using a medical record review: a systematic review
Source: Int J Qual Health Care. 2025 Nov 6;37(4):mzaf119. doi: 10.1093/intqhc/mzaf119 (PMC12622303; doi:10.1093/intqhc/mzaf119)
Supplement: mzaf119_Supplementary_Data [file mzaf119_supplementary_data.zip › Supplement_Data (10)/Supplementary File 1 - Search strategy.docx]

**Supplementary file 1**. Search strategy and terms for each database

Published data 1 January 2000 to 31 October 2024

Language: English and Spanish

Document type: article

Peer Reviewed Published Date: 2000-2024 Narrowed by Language: English and Spanish

| **Database: CINAHL**  **Date: 2024-10-31** | **Search Terms** | **Number of records** |
| --- | --- | --- |
| #1 | TI hospital* OR AB hospital* | 1,800,193 |
| #2 | (MH "Hospitals+") | 331,559 |
| #3 | #1 OR #2 | 1,913,079 |
| #4 | TX “harvard medical practice*” OR AB “harvard medical practice*” | 1,351 |
| #5 | TX hmps OR AB hmps | 6,938 |
| #6 | TX "trigger tool*" OR AB "trigger tool*" | 1,466 |
| #7 | TX gtt OR AB gtt | 34,758 |
| #8 | #4 OR #5 OR #6 OR #7 | 44,092 |
| #9 | TI "adverse event*" OR AB "adverse event*" | 254,492 |
| #10 | TI mistake* OR AB mistake* | 28,738 |
| #11 | TI "medical error*" OR AB "medical error*" | 5,888 |
| #12 | TI "sentinel event*" OR AB "sentinel event*" | 1,152 |
| #13 | TI "adverse effect*" OR AB "adverse effect*" | 216,740 |
| #14 | TI "patient safety" OR AB "patient safety" | 41,361 |
| #15 | TI incident* OR AB incident* | 195,530 |
| #16 | (MH "Health Care Errors+") OR (MH "Treatment Errors+") OR (MH "Medication Errors+") | 124,592 |
| #17 | (MH "Patient Safety+") | 26,949 |
| #18 | #9 OR #10 OR #11 OR #12 OR #13 OR #14 OR #15 OR #16 OR #17 | 840,605 |
| #19 | TI "medical record* review*" OR AB "medical record* review*" | 7,932 |
| #20 | TI mrr OR AB mrr | 1,611 |
| #21 | TI "medical chart* review*" OR AB "medical chart* review*" | 1,945 |
| #22 | (MH "Medical Records+") | 164,294 |
| #23 | #19 OR #20 OR #21 OR #22 | 174,792 |
| #24 | #3 AND #8 AND #18 AND #23 | 107 |

MH = Exact Subject Heading

TI= Title

AB = Abstract

Published data 1 January 2000 to 31 October 2024

Language: English and Spanish

Document type: article

Peer Reviewed Published Date: 2000-2024 Narrowed by Language: English and Spanish

| **Database: MEDLINE**  **Date: 2024-10-31** | **Search Terms** | **Number of records** |
| --- | --- | --- |
| #1 | TI hospital* OR AB hospital* | 1,800,193 |
| #2 | (MH "Hospitals+") | 331,559 |
| #3 | #1 OR #2 | 1,913,079 |
| #4 | TX “harvard medical practice*” OR AB “harvard medical practice*” | 1,351 |
| #5 | TX hmps OR AB hmps | 6,938 |
| #6 | TX "trigger tool*" OR AB "trigger tool*" | 1,466 |
| #7 | TX gtt OR AB gtt | 34,758 |
| #8 | #4 OR #5 OR #6 OR #7 | 44,092 |
| #9 | TI "adverse event*" OR AB "adverse event*" | 254,492 |
| #10 | TI mistake* OR AB mistake* | 28,738 |
| #11 | TI "medical error*" OR AB "medical error*" | 5,888 |
| #12 | TI "sentinel event*" OR AB "sentinel event*" | 1,152 |
| #13 | TI "adverse effect*" OR AB "adverse effect*" | 216,740 |
| #14 | TI "patient safety" OR AB "patient safety" | 41,361 |
| #15 | TI incident* OR AB incident* | 195,530 |
| #16 | (MH "Health Care Errors+") OR (MH "Treatment Errors+") OR (MH "Medication Errors+") | 124,592 |
| #17 | (MH "Patient Safety+") | 26,949 |
| #18 | #9 OR #10 OR #11 OR #12 OR #13 OR #14 OR #15 OR #16 OR #17 | 840,605 |
| #19 | TI "medical record* review*" OR AB "medical record* review*" | 7,932 |
| #20 | TI mrr OR AB mrr | 1,611 |
| #21 | TI "medical chart* review*" OR AB "medical chart* review*" | 1,945 |
| #22 | (MH "Medical Records+") | 164,294 |
| #23 | #19 OR #20 OR #21 OR #22 | 174,792 |
| #24 | #3 AND #8 AND #18 AND #23 | 154 |

MH = Exact Subject Heading

TI= Title

AB = Abstract

Published data 1 January 2000 to 31 October 2024

Language: English and Spanish

Document type: article

Peer Reviewed Published Date: 2000-2024 Narrowed by Language: English and Spanish

| **Database: Cochrane**  **Date: 2024-10-31** | **Search Terms** | **Number of records** |
| --- | --- | --- |
| #1 | (hospital*):ti,ab,kw | 242,242 |
| #2 | MeSH descriptor: [Hospitalization] explode all trees | 20,930 |
| #3 | #1 OR #2 | 247,680 |
| #4 | (Harvard NEXT Medical NEXT Practice*):ti,ab,kw AND (Harvard NEXT Medical NEXT Practice*) | 2 |
| #5 | (hmps):ti,ab,kw AND (hmps) | 13 |
| #6 | (trigger NEXT tool*):ti,ab,kw AND (trigger NEXT tool*) | 39 |
| #7 | (gtt):ti,ab,kw AND (gtt) | 361 |
| #8 | #4 OR #5 OR #6 OR #7 | 409 |
| #9 | (adverse NEXT event*):ti,ab,kw AND (adverse NEXT event*) | 162,457 |
| #10 | (mistake*):ti,ab,kw AND (mistake*) | 1,116 |
| #11 | ("medical NEXT error"):ti,ab,kw AND ("medical NEXT error") | 0 |
| #12 | (sentinel NEXT event*):ti,ab,kw AND (sentinel NEXT event*) | 46 |
| #13 | (adverse NEXT effect*):ti,ab,kw AND (adverse NEXT effect*) | 223,966 |
| #14 | (patient NEXT safety):ti,ab,kw AND (patient NEXT safety) | 9,256 |
| #15 | (incident*):ti,ab,kw AND (incident*) | 9,643 |
| #16 | MeSH descriptor: [Medical Errors] explode all trees | 4,074 |
| #17 | MeSH descriptor: [Patient Safety] explode all trees | 1,092 |
| #18 | #9 OR #10 OR #11 OR #12 OR #13 OR #14 OR #15 OR #16 OR #17 | 363,007 |
| #19 | (medical NEXT record* NEXT review*):ti,ab,kw AND (medical NEXT record* NEXT review*) | 2,701 |
| #20 | (mrr):ti,ab,kw AND (mrr) | 108 |
| #21 | (medical NEXT chart* NEXT review):ti,ab,kw AND (medical NEXT chart* NEXT review) | 130 |
| #22 | MeSH descriptor: [Medical Records] explode all trees | 3,932 |
| #23 | #19 OR #20 OR #21 OR #22 | 6,737 |
| #24 | #3 AND #8 AND #18 AND #23 | 7 |

ti=title

ab=abstract

kw=keywords

Published data 1 January 2000 to 31 October 2024

Language: English and Spanish

Document type: article

Peer Reviewed Published Date: 2000-2024 Narrowed by Language: English and Spanish

| **Database: EMBASE**  **Date: 2024-10-31** | **Search Terms** | **Number of records** |
| --- | --- | --- |
| #1 | hospital*:ab,ti AND [01-01-2000]/sd NOT [31-10-2024]/sd AND [2000-2024]/py | 2,395,016 |
| #2 | 'hospital'/exp AND [embase]/lim | 1,312,164 |
| #3 | #1 OR #2 | 2,991,367 |
| #4 | 'harvard medical practice*':ab,ti | 41 |
| #5 | hmps:ab,ti | 422 |
| #6 | 'trigger tool*':ab,ti | 699 |
| #7 | gtt:ab,ti | 4,214 |
| #8 | #4 OR #5 OR #6 OR #7 | 5,268 |
| #9 | 'adverse event*':ab,ti | 446,403 |
| #10 | mistake*:ab,ti | 48,734 |
| #11 | 'medical error*':ab,ti | 7,642 |
| #12 | 'sentinel event*':ab,ti | 1,687 |
| #13 | 'adverse effect*':ab,ti | 294,094 |
| #14 | 'patient safety':ab,ti | 59,233 |
| #15 | incident*:ab,ti | 271,295 |
| #16 | ('medical error'/exp OR 'patient safety'/exp) AND [embase]/lim | 325,484 |
| #17 | #9 OR #10 OR #11 OR #12 OR #13 OR #14 OR #15 OR #16 | 1,354,360 |
| #18 | 'medical record* review*':ab,ti | 12,928 |
| #19 | mrr:ab,ti | 1,781 |
| #20 | 'medical chart* review*':ab,ti | 3,651 |
| #21 | 'medical record'/exp AND [embase]/lim | 304,322 |
| #22 | #18 OR #19 OR #20 OR #21 | 319,712 |
| #23 | #3 AND #8 AND #17 AND #22 AND ([english]/lim OR [spanish]/lim) AND [embase]/lim AND [01-01-2000]/sd NOT [31-10-2024]/sd AND [2000-2024]/py | 96 |

ti=title

ab=abstract

(hospital*:ab,ti AND [01-01-2000]/sd NOT [01-11-2024]/sd AND [2000-2024]/py OR ('hospital'/exp AND [embase]/lim)) AND ('harvard medical practice*':ab,ti OR hmps:ab,ti OR 'trigger tool*':ab,ti OR gtt:ab,ti) AND ('adverse event*':ab,ti OR mistake*:ab,ti OR 'medical error*':ab,ti OR 'sentinel event*':ab,ti OR 'adverse effect*':ab,ti OR 'patient safety':ab,ti OR incident*:ab,ti OR (('medical error'/exp OR 'patient safety'/exp) AND [embase]/lim)) AND ('medical record* review*':ab,ti OR mrr:ab,ti OR 'medical chart* review*':ab,ti OR ('medical record'/exp AND [embase]/lim)) AND ([english]/lim OR [spanish]/lim) AND [embase]/lim AND [2000-2024]/py
