# Supplementary material for: Predictive values of trigger tools for identifying adverse events in hospitalized patients using a medical record review: a systematic review
Source: Int J Qual Health Care. 2025 Nov 6;37(4):mzaf119. doi: 10.1093/intqhc/mzaf119 (PMC12622303; doi:10.1093/intqhc/mzaf119)
Supplement: mzaf119_Supplementary_Data [file mzaf119_supplementary_data.zip › Supplement_Data (10)/Supplementary File 3, Table 2. Risk of bias assessment.docx]

**Supplementary File 3.** Risk of bias assessment for each included study

**Table 2.** Studies assessed using the Joanna Briggs Institute (JBI) checklist for prevalence studies

| **Author, year and country** | **1. Was the sample frame appropriate to address the target population? (Yes, No, Unclear, Not applicable)** | **2. Were study participants sampled in an appropriate way?**  **(Yes, No, Unclear, Not applicable)** | **3. Was the sample adequate? (Yes, No, Unclear, Not applicable)** | **4. Were the study subjects and the setting described in detail?**  **(Yes, No, Unclear, Not applicable)** | **5. Was the data analysis conducted with sufficient coverage of the identified sample? Was the condition measured in a standard, reliable way for all participants?** | **6. Were valid methods used for the identification of the condition? Was the condition measured in a standard, reliable way for all participants?** | **7. Was the condition measured in a standard, reliable way for all participants? (Yes, No, Unclear, Not applicable)** | **8. Was there appropriate statistical analysis?**  **(Yes, No, Unclear, Not applicable)** | **9. Was the response rate adequate, and if not, was the low response rate managed appropriately? (Yes, No, Unclear, Not applicable)** | **Overall appraisal: Include, Exclude, Seek further info.**  **Comments (Including reason for exclusion)** |
| --- | --- | --- | --- | --- | --- | --- | --- | --- | --- | --- |
| Aibar et al. (2015), Spain (1) | Yes | Yes | Yes | Yes | Yes | Yes | Yes | Yes | NA | Include |
| Aikawa et al. (2021), Japan (2) | Yes | Yes | Yes | Yes | Yes | Yes | Yes | Yes | NA | Include |
| Ali et al. (2024), Australia (3) | Yes | Yes | Yes | Yes | Yes | Yes | Yes | Yes | NA | Include |
| Asavaroengchai et al. (2009), Thailand (4) | Yes | Yes | Yes | Yes | Yes | Yes | Yes | Yes | NA | Include |
| Baker et al. (2004), Canada (5) | Yes | Yes | Yes | Yes | Yes | Yes | Yes | Yes | NA | Include |
| Bates et al. (2023), USA (6) | Yes | Yes | Yes | Yes | Yes | Yes | Yes | Yes | NA | Include |
| Bjertnaes et al. (2015), Norway (7) | Yes | Yes | Yes | Yes | Yes | Yes | Yes | Yes | Yes | Include |
| Brennan et al. (2004), USA (8) | Yes | Yes | Yes | Yes | Yes | Yes | Yes | Yes | NA | Include |
| Brösterhaus et al. (2020), Germany (9) | Yes | Yes | Yes | Yes | Yes | Yes | Yes | Yes | NA | Include |
| Brown et al. (2019), USA (10) | Yes | Yes | Yes | Yes | Yes | Yes | Yes | Yes | NA | Include |
| Carnevali et al. (2013), Belgium (11) | Yes | Yes | Yes | Yes | Yes | Yes | Yes | Yes | NA | Include |
| Classen et al. (2008), USA (12) | Yes | Yes | Yes | Yes | Yes | Yes | Yes | Yes | NA | Include |
| Classen et al. (2011), USA (13) | Yes | Yes | Yes | Yes | Yes | Yes | Yes | Yes | NA | Include |
| Cohen et al. (2005), USA (14) | Yes | Yes | Yes | Yes | Yes | Yes | Yes | Yes | NA | Include |
| Connolly et al. (2012), Ireland (15) | Yes | Yes | Yes | Yes | Yes | Yes | Yes | Yes | NA | Include |
| Croft et al. (2016), USA (16) | Yes | Yes | Yes | Yes | Yes | Yes | Yes | Yes | NA | Include |
| Davis et al. (2002), New Zealand (17) | Yes | Yes | Yes | Yes | Yes | Yes | Yes | Yes | NA | Include |
| Deilkas et al. (2015), Norway (18) | Yes | Yes | Yes | Yes | Yes | Yes | Yes | Yes | NA | Include |
| Deilkas et al. (2017),  Norway and Sweden (19) | Yes | Yes | Yes | Yes | Yes | Yes | Yes | Yes | NA | Include |
| Deilkas et al. (2021), Norway and Sweden (20) | Yes | Yes | Yes | Yes | Yes | Yes | Yes | Yes | NA | Include |
| Dolci et al. (2020), Switzerland (21) | Yes | Yes | Yes | Yes | Yes | Yes | Yes | Yes | NA | Include |
| Dotta et al. (2024), Argentina (22) | Yes | Yes | Yes | Yes | Yes | Yes | Yes | Yes | NA | Include |
| El Saghir et al. (2021), Switzerland (23) | Yes | Yes | Yes | Yes | Yes | Yes | Yes | Yes | NA | Include |
| Fajreldines et al. (2022), Argentina (24) | Yes | Yes | Yes | Yes | Yes | Yes | Yes | Yes | NA | Include |
| Franklin et al. (2009), UK (25) | Yes | Yes | Yes | Yes | Yes | Yes | Yes | Yes | NA | Include |
| Franklin et al. (2010), UK (26) | Yes | Yes | Yes | Yes | Yes | Yes | Yes | Yes | NA | Include |
| Garrrett Jr et al. (2013), USA (27) | Yes | Yes | Yes | Yes | Yes | Yes | Yes | Yes | NA | Include |
| Gómez-López et al. (2019), España (28) | NA | NA | NA | Yes | Yes | Yes | Yes | Yes | NA | Include |
| Good et al. (2011), USA (29) | Yes | Yes | Yes | Yes | Yes | Yes | Yes | Yes | NA | Include |
| Griffey et al. (2020), USA (30) | Yes | Yes | Yes | Yes | Yes | Yes | Yes | Yes | NA | Include |
| Griffin and Classen. (2008), USA (31) | Yes | Yes | Yes | Yes | Yes | Yes | Yes | Yes | NA | Include |
| Grossmann et.al. (2019),  Switzerland (32) | Yes | Yes | Yes | Yes | Yes | Yes | Yes | Yes | NA | Include |
| Gunningberg et al. (2019), Sweden (33) | Yes | Yes | Yes | Yes | Yes | Yes | Yes | Yes | NA | Include |
| Gúzman-Ruíz et al. (2015), Spain (34) | Yes | Yes | Yes | Yes | Yes | Yes | Yes | Yes | NA | Include |
| Härkänen et al. (2015), Finland (35) | Yes | Yes | Yes | Yes | Yes | Yes | Yes | Yes | NA | Include |
| Haukland et al. (2017), Norway (36) | Yes | Yes | Yes | Yes | Yes | Yes | Yes | Yes | NA | Include |
| Hommel et al. (2020), Sweden (37) | Yes | Yes | Yes | Yes | Yes | Yes | Yes | Yes | NA | Include |
| Hu et al. (2019), China (38) | Yes | Yes | Yes | Yes | Yes | Yes | Yes | Yes | NA | Include |
| Hug et al. (2010), USA (39) | Yes | Yes | Yes | Yes | Yes | Yes | Yes | Yes | NA | Include |
| Hwang et al. (2014), Korea (40) | Yes | Yes | Yes | Yes | Yes | Yes | Yes | Yes | NA | Include |
| Hwang et al. (2018), Korea (41) | Yes | Yes | Yes | Yes | Yes | Yes | Yes | Yes | NA | Include |
| Kennerly et al. (2013), USA (42) | Yes | Yes | Yes | Yes | Yes | Yes | Yes | Yes | NA | Include |
| Kennerly et al. (2014), USA (43) | Yes | Yes | Yes | Yes | Yes | Yes | Yes | Yes | NA | Include |
| Kobayashi et al. (2008), Japan (44) | Yes | Yes | Yes | Yes | Yes | Yes | Yes | Yes | NA | Include |
| Kurutkan et al. (2015),  Turkey (45) | Yes | Yes | Yes | Yes | Yes | Yes | Yes | Unclear | NA | Include |
| Letaief et al. (2010), Tunisia (46) | Yes | Yes | Yes | Yes | Yes | Yes | Yes | Yes | NA | Include |
| Lima-Junior et al. (2023), Brazil (47) | Yes | Yes | Yes | Yes | Yes | Yes | Yes | Yes | NA | Include |
| Lipitz-Snyderman et al. (2017), USA (48) | Yes | Yes | Yes | Yes | Yes | Yes | Yes | Yes | NA | Include |
| Magnéli et al. (2019), Sweden (49) | Yes | Yes | Yes | Yes | Yes | Yes | Yes | Yes | NA | Include |
| Mattsson et al. (2014), Denmark (50) | Yes | Yes | Yes | Yes | Yes | Yes | Yes | Yes | NA | Include |
| Mayor et al. (2017), Welch (51) | Yes | Yes | Yes | Yes | Yes | Yes | Yes | Yes | NA | Include |
| Menéndez-Fraga et al. (2021), Spain (52) | Yes | Yes | Yes | Yes | Yes | Yes | Yes | Yes | NA | Include |
| Merten et al. (2013), Dutch (53) | Yes | Yes | Yes | Yes | Yes | Yes | Yes | Yes | NA | Include |
| Mevik et al. (2016), Norway (54) | Yes | Yes | Yes | Yes | Yes | Yes | Yes | Yes | NA | Include |
| Mevik et al. (2019), Norway (55) | Yes | Yes | Yes | Yes | Yes | Yes | Yes | Yes | NA | Include |
| Moraes et al. (2021), Brazil (56) | Yes | Yes | Yes | Yes | Yes | Yes | Yes | Yes | NA | Include |
| Mortaro et al. (2021) Italy (57) | Yes | Yes | Yes | Yes | Yes | Yes | Yes | Yes | NA | Include |
| Mull et al. (2015), USA (58) | Yes | Yes | Yes | Yes | Yes | Yes | Yes | Yes | NA | Include |
| Müller et al. (2016), South Africa (59) | Yes | Yes | Yes | Yes | Yes | Yes | Yes | Yes | NA | Include |
| Naessens et al. (2009), USA (60) | Yes | Yes | Yes | Yes | Yes | Yes | Yes | Yes | NA | Include |
| Naessens et al. (2010), USA (61) | Yes | Yes | Yes | Yes | Yes | Yes | Yes | Yes | NA | Include |
| Najjar et al. (2013), Palestin (62) | Yes | Yes | Yes | Yes | Yes | Yes | Yes | Yes | NA | Include |
| Nilsson et al. (2012), Sweden (63) | Yes | Yes | Yes | Yes | Yes | Yes | Yes | Yes | NA | Include |
| Nilsson et al. (2018),  Sweden (64) | Yes | Yes | Yes | Yes | Yes | Yes | Yes | Yes | NA | Include |
| Nilsson et al. (2020), Sweden (65) | Yes | Yes | Yes | Yes | Yes | Yes | Yes | Yes | NA | Include |
| Nwulu et al. (2013), United Kingdom (66) | Yes | Yes | Yes | No | Yes | Yes | Yes | Yes | NA | Include |
| O´Leary et al. (2013), USA (67) | Yes | Yes | Yes | Yes | Yes | Yes | Yes | Yes | NA | Include |
| Ock et al. (2015), Korea (68) | Yes | Yes | Unclear | Unclear | Yes | Yes | Yes | Yes | NA | Include |
| Otero et al. (2021), Spain (69) | Yes | Yes | Yes | Yes | Yes | Yes | Yes | Yes | NA | Include |
| Pandya et al. (2020), India (70) | Yes | Yes | Yes | Yes | Yes | Yes | Yes | Yes | NA | Include |
| Paulander et al. (2024), Sweden (71) | Yes | Yes | Yes | Yes | Yes | Yes | Yes | Yes | NA | Include |
| Pérez-Zapata et al. (2017), Spain (72) | Yes | Yes | Yes | Yes | Yes | Yes | Yes | Yes | NA | Include |
| Pérez-Zapata et al. (2022), Spain (73) | Yes | Yes | Yes | Yes | Yes | Yes | Yes | Yes | NA | Include |
| Pettersson et al. (2020), Sweden (74) | Yes | Yes | Yes | Yes | Yes | Yes | Yes | Yes | NA | Include |
| Pierdevara et al. (2016), Portugal (75) | Yes | Yes | Yes | Yes | Yes | Yes | Yes | Yes | NA | Include |
| Pierdevara et al. (2020), Portugal (76) | Yes | Yes | Yes | Yes | Yes | Yes | Yes | Yes | NA | Include |
| Resar et al. (2006), USA (77) | Yes | Yes | Yes | Yes | Yes | Yes | Yes | Yes | NA | Include |
| Rutberg et al. (2014), Sweden (78) | Yes | Yes | Yes | Yes | Yes | Yes | Yes | Yes | NA | Include |
| Sajith et al. (2021), Singapore (79) | Yes | Yes | Yes | Yes | Yes | Yes | Yes | Yes | NA | Include |
| Samal et al. (2022), USA (80) | Yes | Yes | Yes | Yes | Yes | Yes | Yes | Yes | NA | Include |
| Sari et al. (2015), Iran (81) | Yes | Yes | Yes | Yes | Yes | Yes | Yes | Yes | NA | Include |
| Scarpis et al. (2023), Italy (82) | Yes | Yes | Yes | Yes | Yes | Yes | Yes | Yes | NA | Include |
| Schildmeijer et al. (2012), Sweden (83) | Yes | Yes | Yes | Unclear | Yes | Yes | Yes | Yes | NA | Include |
| Schmied et al. (2024), Austria (84) | Yes | Yes | Yes | Yes | Yes | Yes | Yes | Yes | NA | Include |
| Sekijima et al. (2020), USA (85) | Yes | Yes | Yes | Yes | Yes | Yes | Yes | Yes | NA | Include |
| Sharek et al. (2011), USA (86) | Yes | Yes | Yes | Yes | Yes | Yes | Yes | Yes | NA | Include |
| Sousa et al. (2014), Portugal (87) | Yes | Yes | Yes | Yes | Yes | Yes | Yes | Yes | NA | Include |
| Storesund et al. (2019), Norway (88) | Yes | Yes | Yes | Yes | Yes | Yes | Yes | Yes | NA | Include |
| Suarez et al. (2014), Spain (89) | Yes | Yes | Yes | Yes | Yes | Yes | Yes | Yes | NA | Include |
| Thomas et al. (2000), USA (90) | Yes | Yes | Yes | Yes | Yes | Yes | Yes | Yes | NA | Include |
| Thomas et al. (2002), USA (91) | Yes | Yes | Yes | Yes | Yes | Yes | Yes | Yes | NA | Include |
| Toribio-Vicente et al. (2018), Spain (92) | Yes | Yes | Yes | Yes | Yes | Yes | Yes | Yes | NA | Include |
| Unbeck et al. (2013), Sweden (93) | Yes | Yes | Yes | Yes | Yes | Yes | Yes | Yes | NA | Include |
| Val et al. (2020), Spain (94) | Yes | Yes | Yes | Yes | Yes | Yes | Yes | Yes | NA | Include |
| Valencia-Martín et al. (2022), Spain (95) | Yes | Yes | Yes | Yes | Yes | Yes | Yes | Yes | NA | Include |
| Valkonen et al. (2023), Finland (96) | Yes | Yes | Yes | Yes | Yes | Yes | Yes | Yes | NA | Include |
| Wilson et al. (2012), Egypt, Jordan, Kenya, Morocco, Tunisia, Sudan, South Africa, and Yemen (97) | Yes | Yes | Yes | Yes | Yes | Yes | Yes | Yes | NA | Include |
| Wong et al. (2015), Canada (98) | Yes | Yes | Yes | Yes | Yes | Yes | Yes | Yes | NA | Include |
| Xu et al. (2020), China (99) | Yes | Yes | Yes | Yes | Yes | Yes | Yes | Yes | NA | Include |
| Zadvinskis et al. (2018), USA (100) | Yes | Yes | Yes | Yes | Yes | Yes | Yes | Yes | NA | Include |

**References**

1. Aibar L, Rabanaque MJ, Aibar C, et al. Patient safety and adverse events related with obstetric care. Archives of gynecology and obstetrics. 2015;291(4):825-30.

2. Aikawa G, Sakuramoto H, Ouchi A, et al. Development of the Japanese version of the Intensive Care Unit Trigger Tool to detect adverse events in critically ill patients. Acute Medicine and Surgery. 2021;8(1).

3. Ali S, Peterson GM, Curtain CM, et al. Adverse Drug Event–Related Hospital Admissions among Australian Aged Care Residents: A Cross-Sectional Study. Journal of the American Medical Directors Association. 2024;25(7):N.PAG-N.PAG.

4. Asavaroengchai S, Sriratanaban J, Hiransuthikul N, et al. Identifying adverse events in hospitalized patients using Global Trigger Tool in Thailand. Asian Biomedicine. 2009;3(5):545-50.

5. Baker GR, Norton PG, Flintoft V, et al. The Canadian Adverse Events Study: the incidence of adverse events among hospital patients in Canada. Cmaj. 2004;170(11):1678-86.

6. Bates DW, Levine DM, Salmasian H, et al. The Safety of Inpatient Health Care. New England Journal of Medicine. 2023;388(2):142-53.

7. Bjertnaes O, Deilkås ET, Skudal KE, et al. The association between patient-reported incidents in hospitals and estimated rates of patient harm. International Journal for Quality in Health Care. 2015;27(1):26-30.

8. Brennan TA, Leape LL, Laird NM, et al. Incidence of adverse events and negligence in hospitalized patients: results of the Harvard Medical Practice Study I. 1991. Quality & safety in health care. 2004;13(2):145-51; discussion 51-52.

9. Brosterhaus M, Hammer A, Kalina S, et al. Applying the Global Trigger Tool in German Hospitals: a Pilot in Surgery and Neurosurgery. Journal of patient safety. 2020;16(4):e340‐e51.

10. Brown SK, Peterson J, Schiedel SH, et al. Evaluation of Trigger Tool Methodology Related to Adverse Drug Events in Hospitalized Patients. Patient Safety (2689-0143). 2019;1(2):14-23.

11. Carnevali L, Krug B, Amant F, et al. Performance of the adverse drug event trigger tool and the global trigger tool for identifying adverse drug events: experience in a Belgian hospital. The Annals of pharmacotherapy. 2013;47(11):1414-9.

12. Classen DC, Lloyd RC, Provost L, et al. Development and evaluation of the institute for healthcare improvement global trigger tool. Journal of Patient Safety. 2008;4(3):169-77.

13. Classen DC, Resar R, Griffin F, et al. ‘Global trigger tool’shows that adverse events in hospitals may be ten times greater than previously measured. Health affairs. 2011;30(4):581-9.

14. Cohen MM, Kimmel NL, Benage MK, et al. Medication safety program reduces adverse drug events in a community hospital. Quality & Safety in Health Care. 2005:169-74.

15. Connolly W, Rafter N, Conroy RM, et al. The Irish National Adverse Event Study-2 (INAES-2): longitudinal trends in adverse event rates in the Irish healthcare system. BMJ Quality & Safety. 2021;30(7):547-58.

16. Croft LD, Liquori ME, Ladd J, et al. Frequency of adverse events before, during, and after hospital admission. Southern Medical Journal. 2016;109(10):631-5.

17. Davis P, Lay-Yee R, Briant R, et al. Adverse events in New Zealand public hospitals I: occurrence and impact. The New Zealand Medical Journal (Online). 2002;115(1167).

18. Deilkås ET, Bukholm G, Lindstrøm JC, et al. Monitoring adverse events in Norwegian hospitals from 2010 to 2013. BMJ open. 2015;5(12):e008576.

19. Deilkås ET, Risberg MB, Haugen M, et al. Exploring similarities and differences in hospital adverse event rates between Norway and Sweden using Global Trigger Tool. BMJ Open. 2017;7(3).

20. Deilkås ET, Haugen M, Risberg MB, et al. Longitudinal rates of hospital adverse events that contributed to death in Norway and Sweden from 2013 to 2018. Journal of Patient Safety and Risk Management. 2021;26(4):153-60.

21. Dolci E, Schärer B, Grossmann N, et al. Automated fall detection algorithm with global trigger tool, incident reports, manual chart review, and patient-reported falls: Algorithm development and validation with a retrospective diagnostic accuracy study. Journal of Medical Internet Research. 2020;22(9).

22. Dotta AT, Duarte Sotelo LE, Biaggioni MA, et al. Detección de eventos adversos en pacientes internados en clínica médica utilizando la herramienta Global Trigger Tool. Medicina (Buenos Aires). 2024;84(1):87-95.

23. El Saghir A, Dimitriou G, Scholer M, et al. Development and Implementation of an e-Trigger Tool for Adverse Drug Events in a Swiss University Hospital. Drug, Healthcare and Patient Safety. 2021;13:251-63.

24. Fajreldines A, Pellizzari M, Valerio M, et al. Eventos adversos asociados al cuidado de la salud en adultos internados en dos hospitales de alta complejidad de Argentina. MEDICINA (Buenos Aires). 2022;82(3).

25. Franklin BD, Birch S, Savage I, et al. Methodological variability in detecting prescribing errors and consequences for the evaluation of interventions. Pharmacoepidemiology and drug safety. 2009;18(11):992-9.

26. Franklin BD, Birch S, Schachter M, et al. Testing a trigger tool as a method of detecting harm from medication errors in a UK hospital: A pilot study. International Journal of Pharmacy Practice. 2010;18(5):305-11.

27. Garrett PR, Jr., Sammer C, Nelson A, et al. Developing and implementing a standardized process for global trigger tool application across a large health system. Joint Commission journal on quality and patient safety. 2013;39(7):292-7.

28. Gómez López VE, Muñoz Macías C, Casas Cuesta R, et al. Análisis de las medidas correctoras para la disminución de los eventos adversos en una unidad de hemodiálisis hospitalaria. Enfermería Nefrológica. 2019;22(1):27-33.

29. Good VS, Saldaña M, Gilder R, et al. Large-scale deployment of the Global Trigger Tool across a large hospital system: Refinements for the characterisation of adverse events to support patient safety learning opportunities. BMJ Quality and Safety. 2011;20(1):25-30.

30. Griffey RT, Schneider RM, Todorov AA. The Emergency Department Trigger Tool: A Novel Approach to Screening for Quality and Safety Events. Annals of Emergency Medicine. 2020;76(2):230-40.

31. Griffin FA, Classen DC. Detection of adverse events in surgical patients using the Trigger Tool approach. Quality & Safety in Health Care. 2008;17(4):253-8.

32. Grossmann N, Gratwohl F, Musy SN, et al. Describing adverse events in medical inpatients using the Global Trigger Tool. Swiss medical weekly. 2019;149:w20149.

33. Gunningberg L, Sving E, Hommel A, et al. Tracking pressure injuries as adverse events: National use of the Global Trigger Tool over a 4‐year period. Journal of Evaluation in Clinical Practice. 2019;25(1):21-7.

34. Guzmán-Ruiz O, Ruiz-López P, Gómez-Cámara A, et al. Detección de eventos adversos en pacientes adultos hospitalizados mediante el método Global TriggerTool. Revista de calidad asistencial. 2015;30(4):166-74.

35. Härkänen M, Kervinen M, Ahonen J, et al. Patient-specific risk factors of adverse drug events in adult inpatients - evidence detected using the Global Trigger Tool method. Journal of Clinical Nursing (John Wiley & Sons, Inc). 2015;24(3-4):582-91.

36. Haukland EC, von Plessen C, Nieder C, et al. Adverse events in hospitalised cancer patients: a comparison to a general hospital population. Acta oncologica (Stockholm, Sweden). 2017;56(9):1218-23.

37. Hommel A, Magneli M, Samuelsson B, et al. Exploring the incidence and nature of nursing-sensitive orthopaedic adverse events: a multicenter cohort study using Global Trigger Tool. International journal of nursing studies. 2020;102:103473‐.

38. Hu Q, Wu B, Zhan M, et al. Adverse events identified by the global trigger tool at a university hospital: A retrospective medical record review. Journal of Evidence-Based Medicine. 2019;12(2):91-7.

39. Hug BL, Witkowski DJ, Sox CM, et al. Adverse drug event rates in six community hospitals and the potential impact of computerized physician order entry for prevention. Journal of general internal medicine. 2010;25(1):31-8.

40. Hwang JI, Chin HJ, Chang YS. Characteristics associated with the occurrence of adverse events: a retrospective medical record review using the Global Trigger Tool in a fully digitalized tertiary teaching hospital in K orea. Journal of Evaluation in clinical practice. 2014;20(1):27-35.

41. Hwang J-I, Kim J, Park J-W. Adverse Events in Korean Traditional Medicine Hospitals: A Retrospective Medical Record Review. Journal of patient safety. 2018;14(3):157-63.

42. Kennerly DA, Saldaña M, Kudyakov R, et al. Description and evaluation of adaptations to the global trigger tool to enhance value to adverse event reduction efforts. Journal of patient safety. 2013;9(2):87-95.

43. Kennerly DA, Kudyakov R, da Graca B, et al. Characterization of adverse events detected in a large health care delivery system using an enhanced global trigger tool over a five‐year interval. Health services research. 2014;49(5):1407-25.

44. Kobayashi M, Ikeda S, Kitazawa N, et al. Validity of retrospective review of medical records as a means of identifying adverse events: comparison between medical records and accident reports. Journal of evaluation in clinical practice. 2008;14(1):126-30.

45. Kurutkan MN, Usta E, Orhan F, et al. Application of the IHI Global Trigger Tool in measuring the adverse event rate in a Turkish healthcare setting. International Journal of Risk & Safety in Medicine. 2015;27(1):11-21.

46. Letaief M, El Mhamdi S, El-Asady R, et al. Adverse events in a Tunisian hospital: results of a retrospective cohort study. International journal for quality in health care. 2010;22(5):380-5.

47. Lima Júnior AJd, Zanetti ACB, Dias BM, et al. Occurrence and preventability of adverse events in hospitals: a retrospective study. Revista Brasileira de Enfermagem. 2023;76:e20220025.

48. Lipitz-Snyderman A, Classen D, Pfister D, et al. Performance of a trigger tool for identifying adverse events in oncology. Journal of Oncology Practice. 2017;13(3):e223-e30.

49. Magnéli M, Unbeck M, Rogmark C, et al. Validation of adverse events after hip arthroplasty: a Swedish multi-centre cohort study. BMJ open. 2019;9(3):e023773.

50. Mattsson TO, Knudsen JL, Brixen K, et al. Does adding an appended oncology module to the Global Trigger Tool increase its value? International Journal for Quality in Health Care. 2014;26(5):553-60.

51. Mayor S, Baines E, Vincent C, et al. Measuring harm and informing quality improvement in the Welsh NHS: the longitudinal Welsh national adverse events study. 2017.

52. Menéndez-Fraga M, Alonso J, Cimadevilla B, et al. Does Skilled Nursing Facility Trigger Tool used with Global Trigger Tool increase its value for adverse events evaluation? Journal of healthcare quality research. 2021;36(2):75-80.

53. Merten H, Zegers M, de Bruijne MC, et al. Scale, nature, preventability and causes of adverse events in hospitalised older patients. Age and ageing. 2013;42(1):87-93.

54. Mevik K, Griffin FA, Hansen TE, et al. Does increasing the size of bi-weekly samples of records influence results when using the Global Trigger Tool? An observational study of retrospective record reviews of two different sample sizes. BMJ open. 2016;6(4):e010700.

55. Mevik K, Hansen TE, Deilkås EC, et al. Is a modified Global Trigger Tool method using automatic trigger identification valid when measuring adverse events? International journal for quality in health care : journal of the International Society for Quality in Health Care. 2019;31(7):535-40.

56. Moraes SM, Ferrari TCA, Figueiredo NMP, et al. Assessment of the reliability of the IHI Global Trigger Tool: new perspectives from a Brazilian study. International journal for quality in health care : journal of the international society for quality in health care. 2021;33(1).

57. Mortaro A, Moretti F, Pascu D, et al. Adverse Events Detection Through Global Trigger Tool Methodology: results From a 5-Year Study in an Italian Hospital and Opportunities to Improve Interrater Reliability. Journal of patient safety. 2021;17(6):451‐7.

58. Mull HJ, Brennan CW, Folkes T, et al. Identifying Previously Undetected Harm: Piloting the Institute for Healthcare Improvement's Global Trigger Tool in the Veterans Health Administration. Quality Management in Health Care. 2015;24(3):140-6.

59. Müller MM, Gous A, Schellack N. Measuring adverse events using a trigger tool in a paper based patient information system at a teaching hospital in South Africa. European journal of clinical pharmacy: atención farmacéutica. 2016;18(2):103-12.

60. Naessens JM, Campbell CR, Huddleston JM, et al. A comparison of hospital adverse events identified by three widely used detection methods. International Journal for Quality in Health Care. 2009;21(4):301-7.

61. Naessens JM, O'Byrne TJ, Johnson MG, et al. Measuring hospital adverse events: assessing inter-rater reliability and trigger performance of the Global Trigger Tool. International Journal for Quality in Health Care. 2010;22(4):266-74.

62. Najjar S, Hamdan M, Euwema MC, et al. The Global Trigger Tool shows that one out of seven patients suffers harm in Palestinian hospitals: challenges for launching a strategic safety plan. International journal for quality in health care. 2013;25(6):640-7.

63. Nilsson L, Pihl A, Tågsjö M, et al. Adverse events are common on the intensive care unit: results from a structured record review. Acta anaesthesiologica Scandinavica. 2012;56(8):959-65.

64. Nilsson L, Borgstedt-Risberg M, Soop M, et al. Incidence of adverse events in Sweden during 2013-2016: a cohort study describing the implementation of a national trigger tool. BMJ open. 2018;8(3):e020833.

65. Nilsson L, Borgstedt-Risberg M, Brunner C, et al. Adverse events in psychiatry: A national cohort study in Sweden with a unique psychiatric trigger tool. BMC Psychiatry. 2020;20(1).

66. Nwulu U, Nirantharakumar K, Odesanya R, et al. Improvement in the detection of adverse drug events by the use of electronic health and prescription records: An evaluation of two trigger tools. European Journal of Clinical Pharmacology. 2013;69(2):255-9.

67. O'Leary KJ, Devisetty VK, Patel AR, et al. Comparison of traditional trigger tool to data warehouse based screening for identifying hospital adverse events. BMJ Quality and Safety. 2013;22(2):130-8.

68. Ock M, Lee S-i, Jo M-W, et al. Assessing Reliability of Medical Record Reviews for the Detection of Hospital Adverse Events. Journal of preventive medicine and public health = Yebang Uihakhoe chi. 2015;48(5):239-48.

69. Otero MJ, Toscano Guzmán MD, Galván-Banqueri M, et al. Utility of a trigger tool (TRIGGER-CHRON) to detect adverse events associated with high-alert medications in patients with multimorbidity. European Journal of Hospital Pharmacy. 2021;28(e1):E41-E6.

70. Pandya AD, Patel K, Rana D, et al. Global Trigger Tool: Proficient Adverse Drug Reaction Autodetection Method in Critical Care Patient Units. Indian Journal of Critical Care Medicine. 2020;24(3):172-8.

71. Paulander J, Ahlstrand R, Bartha E, et al. Events preceding death after high-risk surgery analyzed by Global Trigger Tool and reflective-thematic approach. Acta Anaesthesiologica Scandinavica. 2024;68(10):1481-6.

72. Pérez Zapata AI, Gutiérrez Samaniego M, Rodríguez Cuéllar E, et al. Comparison of the "Trigger" tool with the minimum basic data set for detecting adverse events in general surgery. Revista de calidad asistencial : organo de la Sociedad Espanola de Calidad Asistencial. 2017;32(4):209-14.

73. Pérez Zapata AI, Rodríguez Cuéllar E, de la Fuente Bartolomé M, et al. Predictive Power of the" Trigger Tool" for the detection of adverse events in general surgery: a multicenter observational validation study. Patient safety in surgery. 2022;16(1):7.

74. Pettersson PK, Sköldenberg O, Samuelsson B, et al. The identification of adverse events in hip fracture patients using the global trigger tool: A prospective observational cohort study. International Journal of Orthopaedic and Trauma Nursing. 2020;38:100779.

75. Pierdevara L, Ventura IM, Eiras M, et al. An experience with the Global Trigger Tool for the study of adverse events in a medical ward. Revista de Enfermagem Referência. 2016;4(9):97-105.

76. Pierdevara L, Porcel-Gálvez AM, Maria A, et al. Translation, cross-cultural adaptation, and measurement properties of the portuguese version of the global trigger tool for adverse events. Therapeutics and Clinical Risk Management. 2020;16:1175-83.

77. Resar RK, Rozich JD, Simmonds T, et al. Methods, tools, and strategies. A trigger tool to identify adverse events in the intensive care unit. Joint Commission Journal on Quality & Patient Safety. 2006;32(10):585-90.

78. Rutberg H, Risberg MB, Sjödahl R, et al. Characterisations of adverse events detected in a university hospital: a 4-year study using the Global Trigger Tool method. BMJ open. 2014;4(5):e004879.

79. Sajith SG, Fung DSS, Chua HC. The Mental Health Trigger Tool: Development and Testing of a Specialized Trigger Tool for Mental Health Settings. Journal of Patient Safety. 2021;17(4):e360-e6.

80. Samal L, Khasnabish S, Foskett C, et al. Comparison of a Voluntary Safety Reporting System to a Global Trigger Tool for Identifying Adverse Events in an Oncology Population. Journal of Patient Safety. 2022;18(6):611-6.

81. Sari AA, Doshmangir L, Torabi F, et al. The incidence, nature and consequences of adverse events in Iranian hospitals. Archives of Iranian medicine. 2015;18(12):0-.

82. Scarpis E, Cautero P, Tullio A, et al. Are adverse events related to the completeness of clinical records? Results from a retrospective records review using the Global Trigger Tool. International Journal for Quality in Health Care. 2023;35(4):mzad094.

83. Schildmeijer K, Nilsson L, Årestedt K, et al. Assessment of adverse events in medical care: Lack of consistency between experienced teams using the global trigger tool. BMJ Quality and Safety. 2012;21(4):307-14.

84. Schmied M, Buchberger W, Perkhofer D, et al. Detection of Adverse Events With the Austrian Inpatient Quality Indicators. Journal of Patient Safety. 2023:10.1097.

85. Sekijima A, Sunga C, Bann M. Adverse Events Experienced by Patients Hospitalized without Definite Medical Acuity: A Retrospective Cohort Study. Journal of hospital medicine. 2020;15(1):42-5.

86. Sharek PJ, Parry G, Goldmann D, et al. Performance characteristics of a methodology to quantify adverse events over time in hospitalized patients. Health Services Research. 2011;46(2):654-78.

87. Sousa P, Uva AS, Serranheira F, et al. Estimating the incidence of adverse events in Portuguese hospitals: a contribution to improving quality and patient safety. BMC health services research. 2014;14:311.

88. Storesund A, Haugen AS, Hjortås M, et al. Accuracy of surgical complication rate estimation using ICD-10 codes. British Journal of Surgery. 2019;106(3):236-44.

89. Suarez C, Menendez MD, Alonso J, et al. Detection of Adverse Events in an Acute Geriatric Hospital over a 6-Year Period Using the Global Trigger Tool. Journal of the American Geriatrics Society. 2014;62(5):896-900.

90. Thomas EJ, Studdert DM, Burstin HR, et al. Incidence and types of adverse events and negligent care in Utah and Colorado. Medical care. 2000:261-71.

91. Thomas EJ, Lipsitz SR, Studdert DM, et al. The reliability of medical record review for estimating adverse event rates. Annals of internal medicine. 2002;136(11):812-6.

92. Toribio-Vicente MJ, Chalco-Orrego JP, Díaz-Redondo A, et al. Detección de eventos adversos mediante trigger tools en 2 unidades de hospitalización de un hospital terciario en España. Journal of Healthcare Quality Research. 2018;33(4):199-205.

93. Unbeck M, Schildmeijer K, Henriksson P, et al. Is detection of adverse events affected by record review methodology? an evaluation of the “Harvard Medical Practice Study” method and the “Global Trigger Tool”. Patient safety in surgery. 2013;7(1):1-12.

94. Val RK, López PR, Zapata AP, et al. Detección de eventos adversos en la cirugía tiroidea y paratiroidea utilizando la herramienta trigger y el Conjunto Mínimo de Datos Básicos (CMBD). Journal of Healthcare Quality Research. 2020;35(6):348-54.

95. Valencia‐Martín JL, Vicente‐Guijarro J, San Jose‐Saras D, et al. Prevalence, characteristics, and impact of Adverse Events in 34 Madrid hospitals. The ESHMAD study. European Journal of Clinical Investigation. 2022:e13851.

96. Valkonen V, Haatainen K, Saano S, et al. Evaluation of Global trigger tool as a medication safety tool for adverse drug event detection—a cross-sectional study in a tertiary hospital. European Journal of Clinical Pharmacology. 2023;79(5):617-25.

97. Wilson RM, Michel P, Olsen S, et al. Patient safety in developing countries: retrospective estimation of scale and nature of harm to patients in hospital. Bmj. 2012;344.

98. Wong BM, Dyal S, Etchells EE, et al. Application of a trigger tool in near real time to inform quality improvement activities: a prospective study in a general medicine ward. BMJ quality & safety. 2015;24(4):272-81.

99. Xu X-D, Yuan Y-J, Zhao L-M, et al. Adverse Events at Baseline in a Chinese General Hospital: A Pilot Study of the Global Trigger Tool. Journal of Patient Safety. 2020;16(4):269-73.

100. Zadvinskis IM, Salsberry PJ, Chipps EM, et al. An Exploration of Contributing Factors to Patient Safety. Journal of Nursing Care Quality. 2018;33(2):108-15.
